# Low copy numbers of complement *C4* and *C4A* deficiency are risk factors for myositis, its subgroups and autoantibodies

**DOI:** 10.1136/ard-2022-222935

**Published:** 2022-09-28

**Authors:** Danlei Zhou, Emily H King, Simon Rothwell, Olga Krystufkova, Antonella Notarnicola, Samantha Coss, Rabheh Abdul-Aziz, Katherine E Miller, Amanda Dang, G Richard Yu, Joanne Drew, Emeli Lundström, Lauren M Pachman, Gulnara Mamyrova, Rodolfo V Curiel, Boel De Paepe, Jan L De Bleecker, Antony Payton, William Ollier, Terrance P O'Hanlon, Ira N Targoff, Willy A Flegel, Vidya Sivaraman, Edward Oberle, Shoghik Akoghlanian, Kyla Driest, Charles H Spencer, Yee Ling Wu, Haikady N Nagaraja, Stacy P Ardoin, Hector Chinoy, Lisa G Rider, Frederick W Miller, Ingrid E Lundberg, Leonid Padyukov, Jiří Vencovský, Janine A Lamb, Chack-Yung Yu, for MYOGEN Investigators

**Affiliations:** 1 Center for Microbial Pathogenesis, Abigail Wexner Research Institute, Nationwide Children's Hospital, Columbus, Ohio, USA; 2 Division of Rheumatology, Nationwide Children’s Hospital and Department of Pediatrics, The Ohio State University, Columbus, Ohio, USA; 3 National Institute for Health Research Manchester Biomedical Research Centre, Manchester University NHS Foundation Trust, The University of Manchester, Manchester, UK; 4 Centre for Genetics and Genomics Versus Arthritis, Centre for Musculoskeletal Research, Faculty of Biology, Medicine and Health, University of Manchester, Manchester, UK; 5 Institute of Rheumatology and Department of Rheumatology, Charles University, Prague, Czech Republic; 6 Division of Rheumatology, Department of Medicine Solna, Karolinska Institutet, University Hospital Karolinska, Stockholm, Sweden; 7 Division of Allergy/Immunology and Rheumatology, University at Buffalo Jacobs School of Medicine and Biomedical Sciences, Buffalo, NY, USA; 8 Department of Pediatrics, Northwestern University Feinberg School of Medicine, Chicago, Illinois, USA; 9 Division of Rheumatology, Department of Medicine, George Washington University School of Medicine and Health Sciences, Washington, DC, USA; 10 Department of Neurology, Ghent University Hospital, Ghent, Belgium; 11 Division of Informatics, Imaging and Data Sciences, School of Health Sciences, Faculty of Biology, Medicine and Health, University of Manchester, Manchester, UK; 12 Faculty of Science and Engineering, Manchester Metropolitan University, Manchester, UK; 13 Environmental Autoimmunity Group, Clinical Research Branch, National Institute of Environmental Health Sciences (NIEHS), National Institutes of Health, Bethesda, MD, USA; 14 Veteran’s Affairs Medical Center, University of Oklahoma Health Sciences Center, and Oklahoma Medical Research Foundation, Oklahoma City, OK, USA; 15 Department of Transfusion Medicine, NIH Clinical Center, National Institutes of Health, Bethesda, MD, USA; 16 University of Mississippi Medical Center, Jackson, Mississippi, USA; 17 Department of Microbiology and Immunology, Loyola University Chicago, Maywood, IL, USA; 18 Division of Biostatistics, The Ohio State University, Columbus, Ohio, USA; 19 Division of Population Health, Health Services Research and Primary Care, Faculty of Biology, Medicine and Health, University of Manchester, Manchester, UK

**Keywords:** autoantibodies, dermatomyositis, polymyositis

## Abstract

**Background:**

Idiopathic inflammatory myopathies (IIM) are a group of autoimmune diseases characterised by myositis-related autoantibodies plus infiltration of leucocytes into muscles and/or the skin, leading to the destruction of blood vessels and muscle fibres, chronic weakness and fatigue. While complement-mediated destruction of capillary endothelia is implicated in paediatric and adult dermatomyositis, the complex diversity of complement *C4* in IIM pathology was unknown.

**Methods:**

We elucidated the gene copy number (GCN) variations of total *C4*, *C4A* and *C4B, long* and *short genes* in 1644 Caucasian patients with IIM, plus 3526 matched healthy controls using real-time PCR or Southern blot analyses. Plasma complement levels were determined by single radial immunodiffusion.

**Results:**

The large study populations helped establish the distribution patterns of various *C4* GCN groups. Low GCNs of *C4T* (*C4T*=2+3) and *C4A* deficiency (*C4A*=0+1) were strongly correlated with increased risk of IIM with OR equalled to 2.58 (2.28–2.91), p=5.0×10^−53^ for *C4T*, and 2.82 (2.48–3.21), p=7.0×10^−57^ for *C4A* deficiency. Contingency and regression analyses showed that among patients with *C4A* deficiency, the presence of *HLA-DR3* became insignificant as a risk factor in IIM except for inclusion body myositis (IBM), by which 98.2% had *HLA-DR3* with an OR of 11.02 (1.44–84.4). Intragroup analyses of patients with IIM for C4 protein levels and IIM-related autoantibodies showed that those with anti-Jo-1 or with anti-PM/Scl had significantly lower C4 plasma concentrations than those without these autoantibodies.

**Conclusions:**

*C4A* deficiency is relevant in dermatomyositis, HLA-DRB1*03 is important in IBM and both *C4A* deficiency and HLA-DRB1*03 contribute interactively to risk of polymyositis.

**WHAT IS ALREADY KNOWN ON THIS TOPIC**

- Complement activation causes damage to the skin and muscles in myositis through the formation of membrane attack complexes on capillaries and activation products that stimulate inflammation. How genetic diversity of complement contributes to differential susceptibility among human patients with myositis was unknown.

**WHAT THIS STUDY ADDS**

- We deciphered gene copy number (GCN) variations for complement total C4 (*C4T*), acidic *C4A*, basic *C4B*, long genes (*C4L*) and short genes (*C4S*) in >1600 patients with IIM and >3500 healthy subjects of European ancestry. Low GCNs of *C4T*, *C4A* and *C4L* strongly correlated with elevated risk of juvenile dermatomyositis, adult-onset dermatomyositis and polymyositis. The presence of HLA-*DR3* with deficiencies for *C4A* or *C4B* was the predominant genetic factor for inclusion body myositis. Lower plasma protein levels of C4 and C3 were present among patients with IIM with anti-Jo1 and myositis-associated autoantibodies.

**HOW THIS STUDY MIGHT AFFECT RESEARCH, PRACTICE OR POLICY**

- Low GCN of complement *C4* or *C4A* deficiency are strong risk factors for autoimmunity in IIM. In the presence of myositis autoantibodies, low complement levels could be both a cause (as complement genetic deficiency possibly causes autoimmune disease) and an effect of the disease (through immune-complex-mediated complement consumption). Thus, monitoring depressed levels of complement and elevated activation products would be informative about disease activities or flares.

## Introduction

Idiopathic inflammatory myopathies (IIM) are a group of autoimmune diseases characterised by chronic muscle weakness and fatigue.^1–3^ Pathology in IIM includes the generation of myositis-related autoantibodies and infiltration of leucocytes into muscles and/or the skin leading to inflammation with high levels of muscle enzymes in the circulation.^1^ Four major subgroups of IIM include juvenile dermatomyositis (JDM),^4^ adult-onset dermatomyositis (DM), polymyositis (PM) and inclusion body myositis (IBM). Immune-mediated necrotising myositis and anti-synthetase syndrome are recently defined categories.

JDM is the most common form of myositis in children that has a mean age of diagnosis between 7 and 8 years.^4^ Patients with JDM have similar muscle and skin manifestations as in adult-onset DM but do not have the increased risk of interstitial lung disease (ILD) and malignancy that are more common among adult patients. Specific patterns of rash involving the eyelids, face, shoulders and body areas frequently exposed to sunlight are prevalent among JDM and DM. Muscle weakness is symmetric and proximal to the body axis. In pathognomonic muscle biopsies, there is remarkable complement-mediated destruction of perivascular endothelium leading to perifascicular ischaemia and degeneration of muscle fibres.^5–8^ However, triggers for complement activation and whether complement genetic diversity is engaged in the breakdown of immune tolerance have not been investigated. PM is more common in women over the age of 30. Patients with PM mainly have muscle weakness and may develop ILD but skin manifestations are infrequent. For IBM, the disease starts insidiously at elderly age and weakness may involve both proximal and distal muscles. PM and IBM both seem to involve primarily cell-mediated autoimmunity.^1^

The aetiology of IIM is likely multifactorial. Inflamed muscle cells in patients with IIM express human leucocyte antigen (HLA) class I and sometimes class II proteins that present antigens to T cells and provide activation signals. Many patients with IIM have myositis-specific autoantibodies (MSA) and/or myositis-associated autoantibodies (MAA),^9 10^ which together are termed myositis-related autoantibodies. MAA are also present in other connective tissue diseases. Intriguingly, patients with IIM with the same autoantibodies may present with similar disease patterns and profiles.^9 11^

Among subjects of European ancestry, the presence of HLA-DRB1*03:01 or *HLA-DR3* tends to strongly associate with complement *C4A* deficiency, the presence of a single short *C4B* gene and HLA-B*08:01, which is therefore named the ancestral haplotype AH8.1.^12–18^ Rothwell and colleagues showed that HLA-DRB1*03:01 was one of the strongest risk factors for IIM.^19 20^

**Figure 1:**
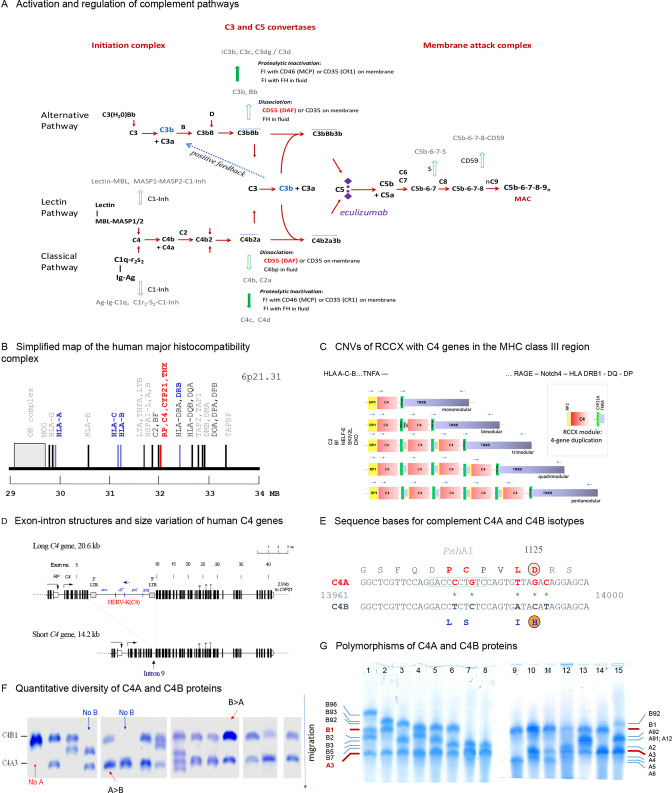
The complement system with emphasis on the genotypic and phenotypic diversities of C4A and C4B. (A) Activation and regulation of the human complement system. Activation of zymogens and progression of pathways are shown in red; regulations of activated products in green. A positive feedback of amplification is common for all three activation pathways. (B). Genetic locations for constituents of the C3 convertases for classical and alternative pathways. (C) Segmental duplications with one to five modules of the *RP-C4-CYP21-TNX* (RCCX) in haplotypes at the class III region of the human leucocyte antigen (HLA). (D) Dichotomy of human *C4* gene size with the long gene containing endogenous retrovirus HERV-K(C4) in the ninth intron and the short gene without the endogenous retrovirus. (E) Specific polymorphisms leading the isotypic changes for C4A and C4B proteins. (F) Immunofixation experiments showing the quantitative and qualitative diversities of C4A and C4B protein allotypes including deficiencies. (G) The range of polymorphic variants for C4B (left panel) and for C4A (right panel). CNVs, copy number variations.

Complement C4 plays essential roles as an anchor protein in the activation of the classical and the mannan-binding lectin pathways for the humoral immunity (figure 1) to defend against infection.^21 22^ There are four layers of genetic complexity for human C4, which include (1) multiallelic gene copy number (GCN) variations with 2–10 copies of *C4* genes present in a diploid genome among different individuals^23–25^; (2) gene size dichotomy with a long gene and a short gene depending on the integration of the 6.4 kb endogenous retrovirus HERV-K(C4) into intron 9 of long genes^26 27^; (3) each *C4* gene either codes for an acidic C4A or a basic C4B protein, which differ by four specific amino acid residues between positions 1120 and 1125 coded by exon 26: PCPVLD for C4A and LSPVIH for C4B^28 29^ and (4) both C4A and C4B proteins are polymorphic with differential electrophoretic, serological and functional reactivities (figure 1).^25 28 30^ Isotype deficiency of C4A has been shown to be strongly associated with increased susceptibility of lupus in multiple racial groups^23 24 31^ and in an animal model.^32^

The role of C4 isotype deficiencies in myositis is understudied. The continuous GCN variations and associated polymorphisms for *C4A* and *C4B* pose challenges for accurate data interpretation through whole-exome or whole-genome sequencing and analyses by Immunochip techniques. In a study of 95 white patients with JDM, we showed that *C4A* deficiency was a strong risk factor for JDM.^33^ How complement *C4* genetic diversity contributes to disease predisposition in different forms of IIM, the development of MSA and/or MAA and the relative roles of HLA-DRB1*03 and *C4A* deficiency in IIM have yet to be assessed, however.

We leveraged a robust collection of biospecimens and clinical data for patients with IIM recruited by Investigators of the Myositis Genetics Consortium (MYOGEN) from the UK, Sweden, the Czech Republic, Belgium and the USA, plus geographically matched healthy controls to investigate the GCN variations of total *C4* (*C4T*), *C4A*, *C4B*, long *C4* genes (*C4L*) and short *C4* genes (*C4S*) in disease susceptibility for IIM and its four major subtypes. The relative roles of HLA-DRB1*03 and *C4A* deficiency on genetic risk of IIM, and how the *C4* GCN variations and complement protein levels correlated with the presence of myositis-related autoantibodies were also examined.

## Patients and methods

### Study populations

Our study population included 1644 patients with IIM and 3526 healthy controls (table 1). Patients with IIM fulfilled Bohan and Peter classification criteria for DM, JDM and PM,^34 35^ and Griggs or European Neuromuscular Center or the UK Medical Research Council criteria for IBM.^36–38^ Study subjects were recruited with informed consent from northern and central Europe and the USA through the MYOGEN or at the Nationwide Children’s Hospital, were self-reported European ancestry or based on principal component analysis.^19^ Patients with non-European ancestries were studied but not included in specific genetic analyses. Healthy control subjects did not report to have an autoimmune disease.

**Table 1:** Demographics of study populations

	Patients with IIM	Controls		
N	1644	3526		
Sex, F: M	0.668 : 0.332	0.730 : 0.270		
Age at disease diagnosis or recruitment (controls), years old	50.5±16.3	62.5±6.2		
Sources	n	n	n (total)	
Belgium	36	–	36	
Czech Republic	329	96	425	
UK	760	1451	2211	
USA	138	945	1083	
Sweden	381	1034	1415	
Total	1644	3526	5170	
Subgroups of patients with IIM, n (%)*				
	F	M	Total	F/M
DM	391 (69.3)	173 (30.7)	564	2.26
PM	473 (71.2)	191 (28.8)	664	2.48
IBM	75 (41.7)	105 (58.3)	180	0.71
JDM	103 (67.3)	50 (32.7)	153	2.06
Overall	1042	519	1561*	(2.01)
	Age at diagnosis (years old)	MSA**+**, %	MAA**+**, %	
DM	49.2±15.0	65.4	28.7	
PM	50.4±14.2	42.7	38.6	
IBM	61.2±9.3	2.4	19.3	
JDM	7.9±4.3	42.9	22.7	

*The patient population with IIM also included 28 patients diagnosed with immune-mediated necrotising myositis, 24 patients with anti-synthetase syndrome, 19 patients with unspecified subcategory of disease, plus 12 patients with gender unknown.

DM, dermatomyositis; IBM, inclusion body myositis; IIM, idiopathic inflammatory myopathies; JDM, juvenile dermatomyositis; MAA, myositis-associated autoantibodies; MSA, myositis-specific autoantibodies; PM, polymyositis.

### Isolation of genomic DNA, EDTA-plasma and Southern blot analyses

For subjects recruited in Ohio, preparation of genomic DNA from peripheral blood samples, performance of *Taq*I, *Psh*AI-*Pvu*II restriction fragment length polymorphisms and *Pme*I pulsed-field gel electrophoresis to elucidate *RP-C4-CYP21-TNX* (RCCX) modular structures were as described.^39^

### Copy numbers and sizes of *C4A* and *C4B* genes by real-time PCR

When quantities of genomic DNA were limiting, copy numbers of *C4* genes were determined by TaqMan-based quantitative real-time PCR with internal control using cosmid DNA with both test and control amplicons. Five independent test amplicons specific for total *C4* (*C4T*), *C4A*, *C4B*, long genes and short genes were performed. Verification was achieved when GCN of *C4T*=GCNs of *C4A+C4B* and/or GCNs of *C4L+C4S*.^14^

### Protein concentrations and polymorphic variants

Complement C4 and C3 protein concentrations were measured by single radial immunodiffusion (RID) using EDTA-plasma and an RID kit from the Binding Site (UK). C4A and C4B protein allotypes in plasma samples were resolved by high-voltage agarose gel electrophoresis, followed by immunofixation using antiserum against human C4.^40^

### Genotyping of *HLA-DRB1*

Genotyping for *HLA-DRB1* alleles for samples from the USA and Sweden was performed at low resolution using the sequence-specific primer-PCR methods (eg, DR low-resolution kit: Olerup SSP, Saltsjobaden, Sweden).^41 42^ The *HLA-DRB1* genotypes for samples from the UK were deduced from single-nucleotide polymorphisms data using SNP2HLA software.^20 43 44^ High concordance of imputed data from DNA sequencing and conventional HLA typing techniques was obtained.^20^

### Statistical analyses

This was a cross-sectional, case–control study. Statistical analyses were performed using JMP16 software from SAS. Continuous data between patients and controls were compared by t-tests. The distributions of *C4T*, *C4A*, *C4B*, *C4L* and *C4S* GCN groups in patients with IIM or in each IIM subgroup and controls were analysed by χ^2^ analyses. The GCN groups for each type of *C4* genes were segregated dichotomously into low GCN and medium to high GCN groups, and their frequencies compared between case and controls with χ^2^ analyses to compute ORs and 95% CIs. The low GCN groups were defined as follows: *C4T*=2+3, *C4A*=0+1, *C4B*=0+1, *C4L*=0+1+2 and *C4S*=0. A Bonferroni’s correction for a *C4* genotype with p<0.01 was considered significant to account for five structural variants being investigated for IIM genetic risk individually. For intragroup comparisons of a specific phenotype with a genotype, a p-value <0.05 was viewed as significant.

## Results

### Comparisons of GCN variations of complement *C4* between IIM and controls

#### Total *C4*

**Figure 2:**
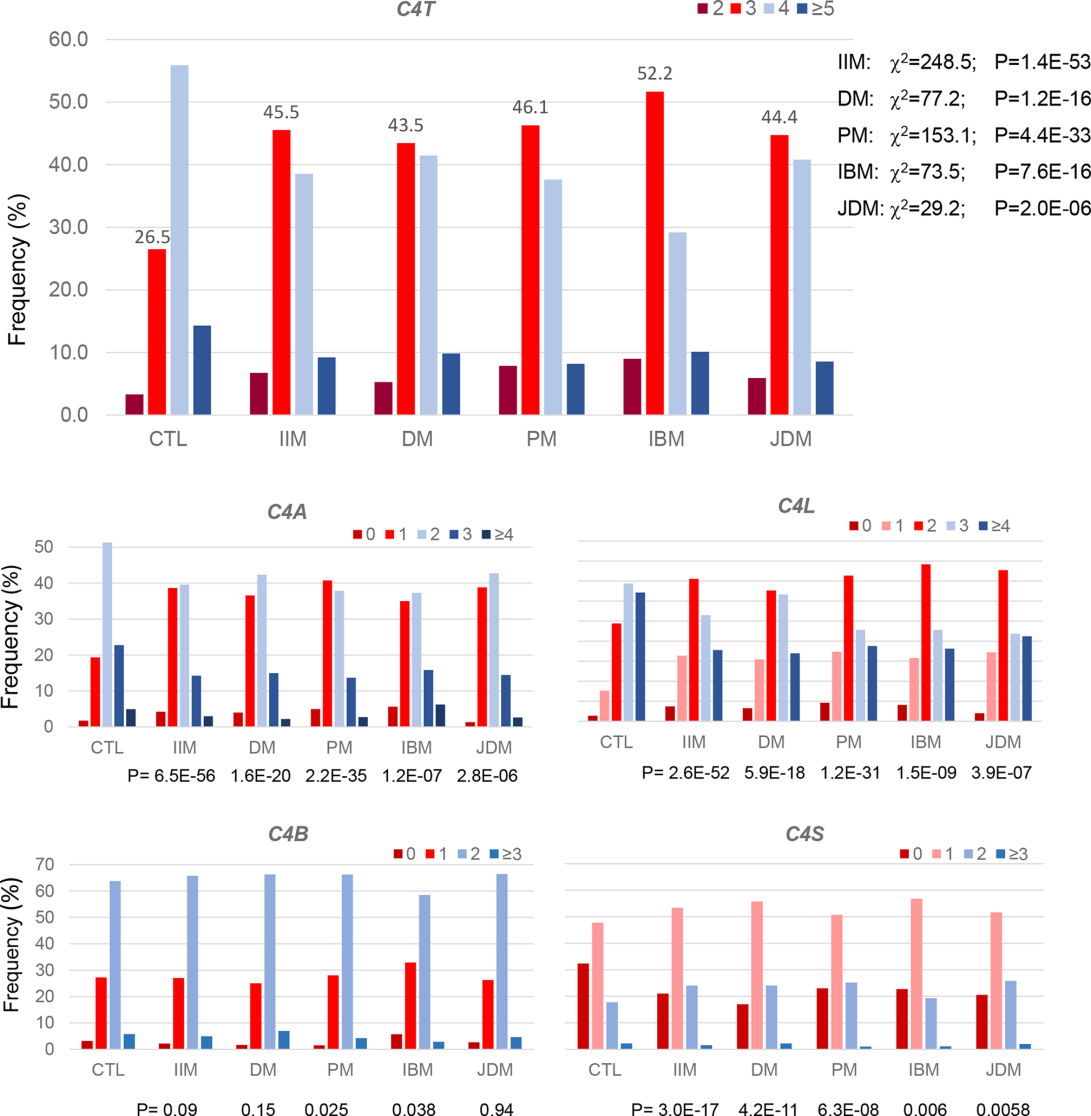
Comparisons in patterns of distributions for complement *C4* gene copy number groups for total *C4*, *C4A*, *C4B*, *C4L* and *C4S* among healthy controls (CTL) and patients with idiopathic inflammatory myopathies (IIM) including adult dermatomyositis (DM), polymyositis (PM), inclusion body myositis (IBM) and juvenile dermatomyositis (JDM). Frequencies with three copies of total C4 (*C4T*) were labelled to highlight the difference between patients and CTL. *C4A*, acidic isotype of complement C4; *C4B*, basic isotype of complement C4; *C4L*, long form of *C4* gene with human endogenous retrovirus HERV-K(C4); *C4S*, short form of *C4* gene without integration of the retrovirus HERV-K(C4); *C4T*, total copy number of *C4* genes.

**Figure 3:**
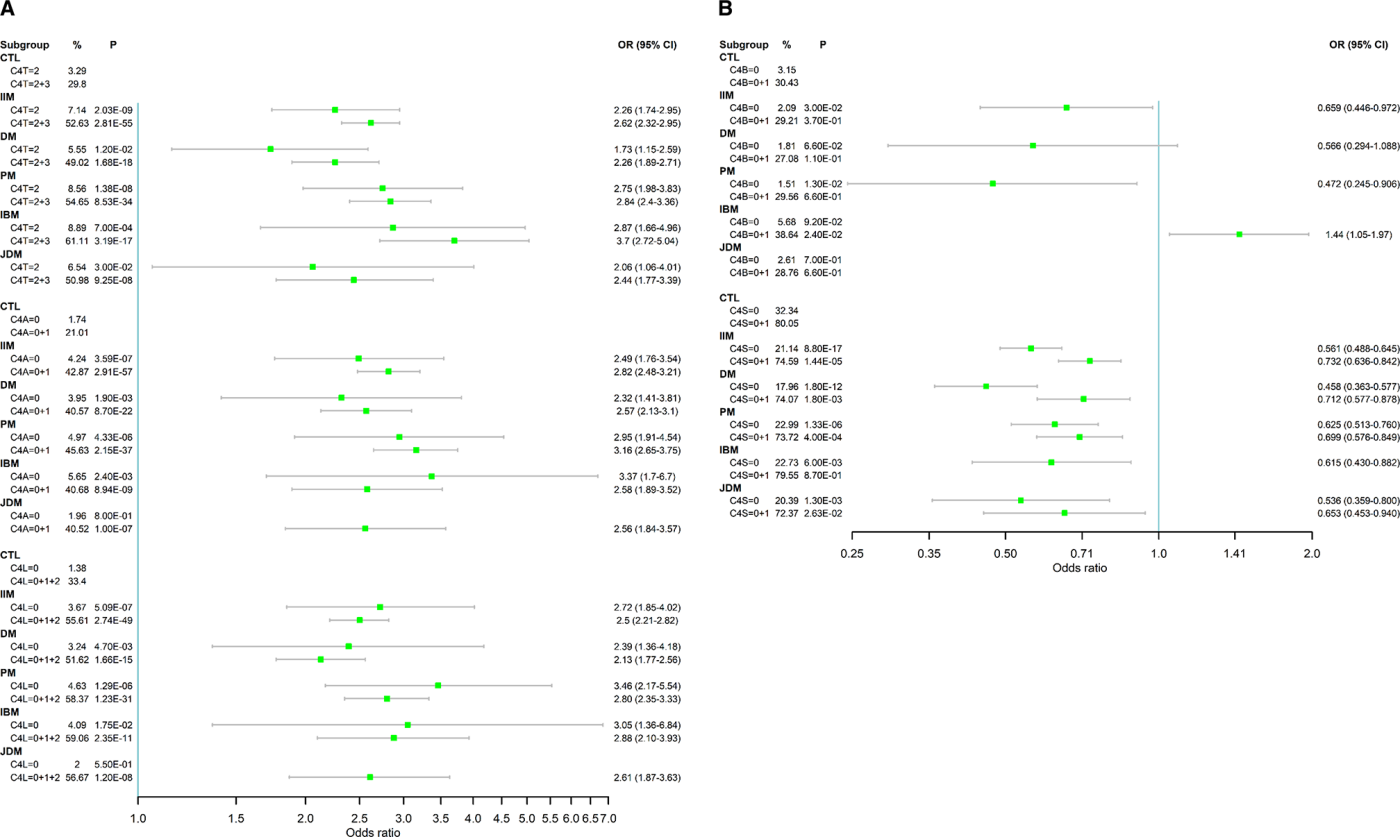
Forest plots of ORs for low copy number groups for *C4T*, *C4A*, long genes (*C4L*) as risk factors (A), and for *C4B* and short genes (*C4S*) as protective factors (B) in IIM and subgroups. A single exception was that low copy number *C4B* was also a risk factor of IBM. Notice the partial dominance of low GCNs of total *C4* (*C4T*=2 and C4T=2+3) and *C4A* deficiencies (*C4A*=0 and *C4A*=0+1) on conferring risk of IIM and its subgroups DM, PM, IBM and JDM. The ORs in panel A are shown in log-scale. *C4A*, acidic isotype of complement C4; *C4B*, basic isotype of complement C4; *C4L*, long form of *C4* gene with human endogenous retrovirus HERV-K(C4); *C4S*, short form of *C4* gene without integration of the retrovirus HERV-K(C4); *C4T*, total copy number of *C4* genes; DM, dermatomyositis; GCNs, gene copy numbers; IBM, inclusion body myositis; IIM, idiopathic inflammatory myopathies; JDM, juvenile dermatomyositis; PM, polymyositis.

The mean GCN and SD of *C4T* among patients with IIM was 3.50±0.78, compared with 3.83±0.76 in healthy controls (δ=−0.333, p=1.4×10^−46^, t-test) (table 2). The most prevalent GCN group for *C4T* in IIM was 3 copies with a frequency of 45.5%, followed by a GCN group of 4 copies with a frequency of 38.2% (figure 2). Patients with 2 copies of *C4T* comprised 7.1%, and those with 5, 6, 7 and 8 copies comprised a total of 9.2% of all IIM. Categorically, the distributions of *C4T* GCNs in IIM were substantially different from those in healthy controls, with a p-value of 1.4×10^−53^ (χ^2^ analysis). The OR and 95% CI for IIM subjects with two copies of *C4T* was 2.26 (1.74 to 2.95), p=2.0×10^−9^, and those with two or three copies (*C4T*=2+3) had an OR=2.62 (2.32 to 2.95), p=2.8×10^−55^ (figure 3A; see also online supplemental figure S2, supplementary results). Thus, low GCNs of *C4T*, that is, *C4T*=2 and *C4T*=2+3, had similar magnitude of effects on the genetic risk of IIM.

10.1136/ard-2022-222935.supp1 Supplementary data

**Table 2:** Comparisons of mean complement *C4* gene copy numbers (GCN) among patients with IIM

	n*	GCN, mean±SD	Difference with control	P value†
*C4T*				
Controls	3500	3.83±0.76		
IIM	1637	3.50±0.78	−0.34	1.4E-46
DM	559	3.56±0.76	−0.28	1.7E-15
PM	665	3.46±0.79	−0.38	1.8E-30
IBM	180	3.40±0.79	−0.43	1.3E-13
JDM	153	3.52±0.76	−0.32	4.9E-07
*C4A*				
Controls	3499	2.10±0.84		
IIM	1628	1.74±0.88	−0.37	6.0E-46
DM	557	1.75±0.85	−0.35	6.8E-20
PM	664	1.70±0.91	−0.41	1.8E-29
IBM	177	1.82±0.98	−0.28	1.3E-05
JDM	153	1.78±0.84	−0.33	2.6E-06
*C4B*				
Controls	3497	1.73±0.62		
IIM	1623	1.74±0.59	0.01	0.45
DM	554	1.78±0.60	0.05	0.040
PM	663	1.73±0.56	0.008	0.76
IBM	176	1.59±0.65	−0.14	0.0039
JDM	153	1.74±0.59	0.014	0.79
*C4L*				
Controls	3475	2.94±1.08		
IIM	1551	2.41±1.13	−0.53	1.7E-54
DM	525	2.47±1.11	−0.47	2.5E-20
PM	626	2.37±1.17	−0.57	1.5E-32
IBM	171	2.36±1.10	−0.58	1.1E-11
JDM	150	2.43±1.11	−0.51	2.1E-08
*C4S*				
Controls	3478	0.90±0.77		
IIM	1595	1.06±0.72	0.16	3.0E-12
DM	540	1.10±0.71	0.20	8.5E-09
PM	647	1.05±0.74	0.15	8.6E-06
IBM	176	0.99±0.68	0.09	0.14
JDM	152	1.09±0.73	0.19	0.0026

*n represents the number of subjects by which the *C4* GCN in each category was successfully determined. DM, PM, IBM and JDM are subgroups of IIM.

†P-values obtained by comparing with controls.

*C4A*, acidic isotype of complement C4; *C4B*, basic isotype of complement C4; *C4L*, long form of *C4* gene with human endogenous retrovirus HERV-K(C4); *C4S*, short form of *C4* gene without integration of the retrovirus HERV-K(C4); *C4T*, total copy number of *C4* genes; DM, dermatomyositis; IBM, inclusion body myositis; IIM, idiopathic inflammatory myopathies; JDM, juvenile dermatomyositis; PM, polymyositis.

#### *C4A* in IIM

GCN of *C4A* varied from 0 to 6 among patients with IIM with a mean of 1.74±0.88, compared with 2.10±0.84 in healthy controls (δ=−0.37, p=6.0×10^−46^). There were remarkable increases in the frequencies of *C4A* low GCN groups and decreases in medium and high GCN groups in IIM (p=6.5×10^−56^). While 40.1% of patients with IIM had two copies of *C4A* genes, those with 0 and 1 copy constituted 4.2% and 38.6% of patients, respectively (figure 2). Patients with 3–6 copies (high GCN) of *C4A* together had a combined frequency of 17.1%. The OR was 2.49 (1.76–3.54, p=3.6×10^−7^) for *C4A*=0 and 2.82 (2.48–3.21, p=2.9×10^−57^) for *C4A*=0+1 (figure 3A). The magnitudes of the effects of low *C4A* GCNs on IIM were similar to that observed in *C4T*=2 and *C4T*=2+3.

#### *C4B* in IIM

Unlike *C4T* and *C4A*, *C4B* copy number group distribution in IIM was almost identical to that observed in healthy controls, which ranged between 0 and 5. Close to two-thirds of the patients with IIM (65.8%) had two copies of *C4B*, while 2.1% and 27.1% had 0 and 1 copy, respectively. Patients with 3, 4 and 5 copies of *C4B* constituted a total frequency of 4.9%.

#### Long genes (*C4L*) in IIM

The copy number of *C4L* varied from 0 to 8 in patients with IIM. The mean *C4L* GCN in IIM was 2.41±1.13, which was significantly lower than that in healthy controls (2.94±1.08, p=1.7×10^−54^). The distribution of GCN groups for *C4L* was different from that of controls (p=2.6×10^−52^). The combined frequency for low GCN of long genes (*C4L*=0+1+2) in IIM was 55.6%, compared with 33.4% in healthy controls (OR=2.50 (2.21–2.82), p=2.7×10^−49^) (figure 3A). Decreasing GCNs of *C4L* elevated the ORs for IIM: 2.55 (2.15–3.03, p=9.4×10^−27^) for *C4L*=0+1 and 2.72 (1.85–4.02, p=5.1×10^−7^) for *C4L*=0. The frequency of long genes among total *C4* decreased from 74.6% in controls to 63.2% in IIM (*C4L*/*C4T*, p=2.1×10^−53^).

#### Short genes (*C4S*) in IIM

The copy number of *C4S* in IIM varied from 0 to 5. The mean copy number was 1.06±0.72, which was *higher* than that in healthy controls (0.90±0.77, p=3.0×10^−12^). More than half of the patients with IIM had a single copy of *C4S* (53.5%). The frequency of subjects lacking *C4S* (*C4S*=0) was significantly reduced from 32.3% in controls to 21.1% in IIM (OR=0.56 (0.49–0.65), p=8.8×10^−17^).

### *C4* GCN variations among subgroups of IIM

Compared with controls, the four IIM subgroups had lower mean GCNs of *C4A* in the range of 1.70 to 1.82 but they were not distinguishable among themselves (table 2 and figure 2). Patients with IBM were unusual for having lower GCNs of *C4B* (1.59±0.65) than other IIM subgroups. In the other three subgroups, lower *C4T* GCN was primarily attributable to the decreased GCN of *C4A*.

As shown in figure 3, the effect sizes of *C4T*=2+3, *C4A*=0+1 and *C4L*=0+1+2 on IIM subgroups were similar, with ORs ranging between 2.1 and 3.7. Low *C4T* GCN had the greatest impact on IBM with OR=3.70 (2.72–5.04). Low *C4A* GCN had the largest impact on PM with OR=3.16 (2.65–3.75). Low *C4L* GCN had largest effects on PM and IBM, with ORs of 2.80 and 2.88, respectively.

### *C4* GCN variations among patients with IIM with and without MSA or MAA

We compared the mean age at diagnosis, sex and *C4* GCN variations between patients with IIM with and without various myositis-related autoantibodies (table 3). Patients with anti-Jo1, anti-PM/Scl and MAA in general had younger age of disease diagnosis between 43 and 49 years old. Patients with IIM who tested positive for MSA or MAA were more likely to be women (70%–75%). Patients with anti-Jo1 and anti-PM/Scl consistently had the lowest mean GCNs of *C4T*, *C4A* and *C4L*.

**Table 3:** Intragroup comparisons of demographics and complement *C4* genes in patients with IIM with and without MSA or MAA

Continuous data	Ab-negative	Ab-positive	P value	
Age of diagnosis, mean±SD (years old)				
MSA	51.8±14.6	51.3±14.2	0.66	
MAA	52.9±14.0	47.6±15.0	2.2E-05	
MSA-Jo1	52.2±14.6	48.9±13.1	0.02	
MAA-PM/Scl	52.3±14.1	43.6±15.1	8.5E-06	
MAA-Ro	51.5±14.5	52.5±13.3	0.63	
*C4T*, GCN, mean±SD				
MSA	3.46±0.78	3.51±0.78	0.29	
MAA	3.55±0.79	3.39±0.75	0.0004	
MSA-Jo1	3.55±0.78	3.25±0.75	2.4E-08	
MAA-PM/Scl	3.53±0.79	3.13±0.67	2.4E-06	
MAA-Ro	3.46±0.78	3.40±0.72	0.38	
*C4A* GCN, mean±SD				
MSA	1.79±0.92	1.68±0.86	0.02	
MAA	1.80±0.89	1.59±0.85	3.9E-05	
MSA-Jo1	1.79±0.87	1.43±0.86	2.0E-09	
MAA-PM/Scl	1.78±0.90	1.34±0.72	5.3E-06	
MAA-Ro	1.69±0.88	1.52±0.77	0.028	
*C4B* GCN, mean±SD				
MSA	1.68±0.59	1.79±0.56	0.0005	
MAA	1.73±0.59	1.77±0.54	0.143	
MSA-Jo1	1.73±0.59	1.80±0.52	0.087	
MAA-PM/Scl	1.73±0.59	1.79±0.46	0.329	
MAA-Ro	1.74±0.60	1.83±0.51	0.072	
*C4L* GCN, mean±SD				
MSA	2.35±1.11	2.40±1.14	0.454	
MAA	2.48±1.14	2.27±1.12	0.0017	
MSA-Jo1	2.50±1.13	2.02±1.11	2.1E-09	
MAA-PM/Scl	2.47±1.14	1.80±1.00	1.7E-07	
MAA-Ro	2.36±1.17	2.35±1.10	0.91	
*C4S* GCN, mean±SD				
MSA	1.04±0.70	1.10±0.74	0.093	
MAA	1.03±0.72	1.11±0.72	0.055	
MSA-Jo1	1.02±0.71	1.26±0.72	1.2E-06	
MAA-PM/Scl	1.03±0.71	1.35±0.61	6.8E-05	
MAA-Ro	1.08±0.72	1.13±0.69	0.50	

Categorical data	Ab-negative	Ab-positive	P value	OR
Sex, female, %				
MSA	62.1	70.7	0.001	1.47 (1.17–1.86)
MAA	64.3	72.8	0.0012	1.49 (1.17–1.90)
MSA-Jo1	66.3	69.4	0.33	ns
MAA-PM/Scl	65.2	74.7	0.059	1.58 (0.968–2.57)
MAA-Ro	65.6	73.9	0.051	1.49 (0.983–2.24)
*C4T*=2+3, frequency, %				
MSA	56.0	51.7	0.119	
MAA	49.1	61.4	1.2E-05	1.65 (1.32–2.07)
MSA-Jo1	49.5	69.8	1.3E-09	2.36 (1.77–3.14)
MAA-PM/Scl	49.9	80.2	7.5E-09	4.08 (2.40–6.92)
MAA-Ro	54.1	59.1	0.27	
*C4A*=0+1, frequency, %				
MSA	40.7	45.6	0.077	1.22 (0.98–1.52)
MAA	39.0	52.0	3.6E-06	1.69 (1.36–2.12)
MSA-Jo1	38.7	63.6	1.7E-13	2.77 (2.10–3.66)
MAA-PM/Scl	39.8	67.0	4.6E-07	3.08 (1.96–4.85)
MAA-Ro	45.0	54.0	0.05	1.44 (0.999–2.07)
*C4B*=0+1, frequency, %				
MSA	32.9	26.0	0.0065	0.72 (0.56–0.91)
MAA	31.0	25.0	0.018	0.74 (0.58–0.95)
MSA-Jo1	30.7	24.3	0.057	0.74 (0.55–1.01)
MAA-PM/Scl	30.7	22.2	0.085	0.65 (0.39–1.08)
MAA-Ro	30.1	21.0	0.026	0.62 (0.40–0.96)
*C4L*=0+1+2, frequency, %				
MSA	58.0	55.1	0.30	
MAA	52.4	62.0	0.0009	1.48 (1.17–1.87)
MSA-Jo1	51.6	73.5	1.1E-10	2.60 (1.92–3.52)
MAA-PM/Scl	53.1	82.1	6.6E-08	4.07 (2.30–7.21)
MAA-Ro	57.9	55.8	0.653	
*C4S*=0, frequency, %				
MSA	21.6	19.8	0.43	
MAA	22.4	18.6	0.11	
MSA-Jo1	23.1	12.4	6.2E-05	0.47 (0.32–0.70)
MAA-PM/Scl	22.6	6.0	4.9E-05	0.22 (0.09–0.54)
MAA-Ro	20.8	16.5	0.253	

*C4A*, acidic isotype of complement C4; *C4B*, basic isotype of complement C4; *C4L*, long form of *C4* gene with human endogenous retrovirus HERV-K(C4); *C4S*, short form of *C4* gene without integration of the retrovirus HERV-K(C4); GCN, gene copy number; IIM, idiopathic inflammatory myopathies; MAA, myositis-associated autoantibodies; MSA, myositis-specific autoantibodies.

**Figure 4:**
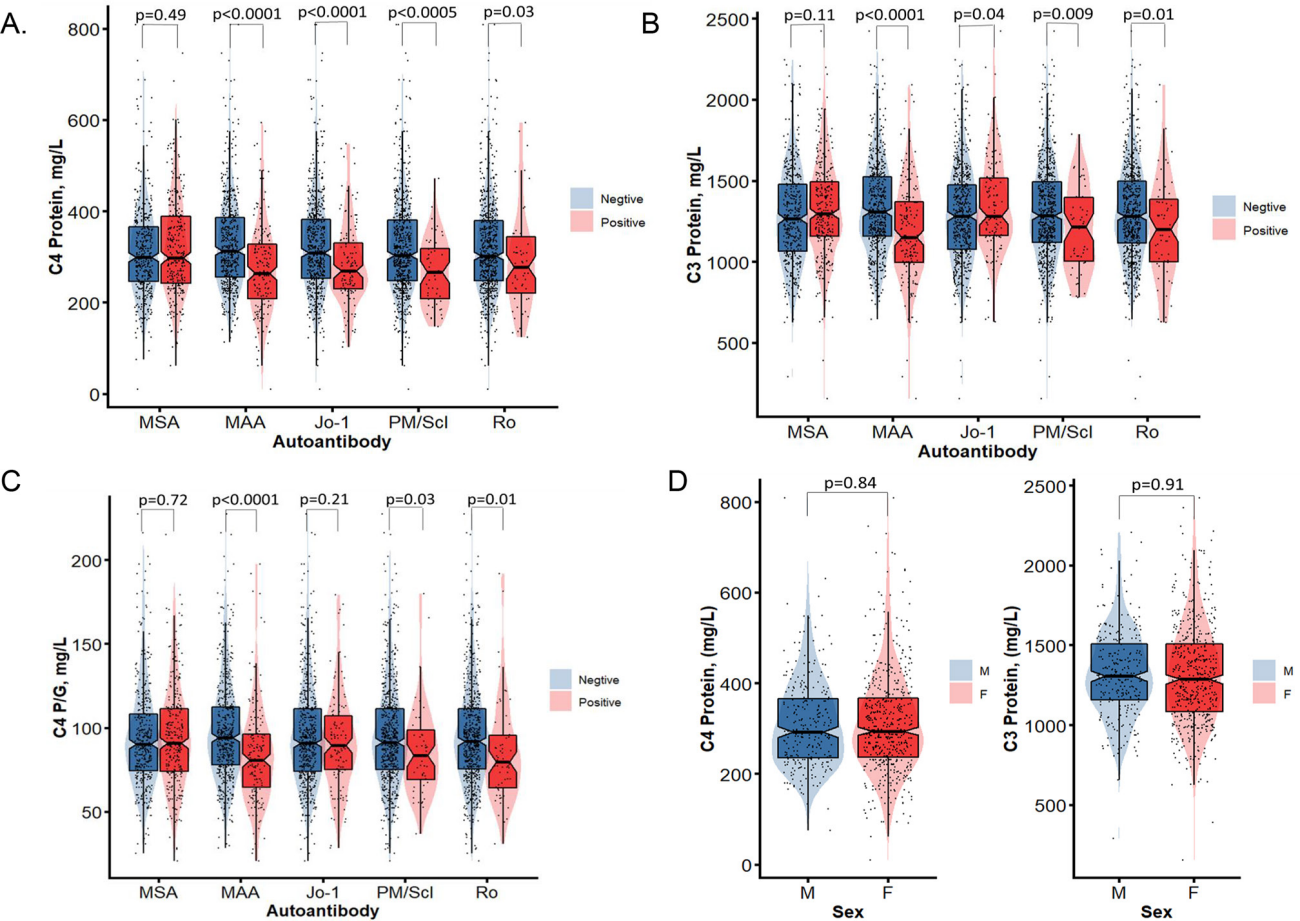
Comparisons of plasma protein levels for complement C4, C3 and C4 protein yield per *C4* gene copies among patients with IIM with (+) and without (–) myositis-related autoantibodies including MSA or MAA in general, and anti-Jo1, anti-PM/Scl and anti-Ro (A, B and C). (D and E) Comparisons of C4 protein levels and C4/G between male and female patients with IIM. Violin plots are shown with median, 25th and 75th percentage range marked as boxes; red colour shades represent positive and blue colour shades represent negative with the specific autoantibodies of women and men, respectively, with p-values shown above. IIM, idiopathic inflammatory myopathies; MAA, myositis-associated autoantibodies; MSA, myositis-specific autoantibodies.

Except for anti-Jo1, patients with MSA presented with similar C4 or C3 plasma protein concentrations than those without. In contrast, patients with MAA had significantly lower levels of C4 and C3 than those without MAA (C4: 275.1±100.0 vs 330.9±105.4 mg/L, p=2.1×10^−9^; C3: 1188.0±309.8 vs 1335.2±283.9 mg/L, p=1.2×10^−8^). With regards to specific autoantibodies, patients with anti-PM/Scl and anti-Ro each had significantly lower C4 and C3 protein levels than those without these autoantibodies (figure 4A, B). Patients with MAA (83.6±30.6 vs 98.0±31.6 mg/L, p=1.1×10^−7^), anti-PM/Scl (86.1±26.7 vs 95.2±31.1 mg/L, p=0.03) and anti-Ro (85.0±30.1 vs 95.4±30.8 mg/L, p=0.014) had significantly lower C4 protein yield per gene (C4P/G) than those without these autoantibodies. No significant differences were observed on plasma protein levels of C4 and C4P/G between women and men (figure 4D, E).

### Logistic regression analyses of HLA-DRB1*03 and *C4A* deficiency in genetic risk of IIM and IIM-related autoantibodies

Among healthy control subjects, 26.1% were HLA-DRB1*03 positive, compared with 56.1% in patients with IIM, which translated into an OR of 3.68 (2.94–4.60, p=2.6×10^−32^) in IIM (table 4). The distribution of HLA-DRB1*03 was uneven among subgroups of IIM, which varied from 75.4% in patients with IBM with an OR of 8.71 (5.48–13.8) to 59.5% in patients with PM with an OR of 4.16 (3.15–5.48), 47.6% in patients with DM with an OR of 2.57 (1.90–3.49) and 45.5% in patients with JDM with an OR of 2.36 (1.56–3.79).

**Table 4:** Logistic regression models for genetic predictors in IIM, subgroups and autoantibodies

	Predictors	χ^2^	OR (95% CI)	P value
**A. Genetic predictors for IIM and subgroups**				
IIM	n=1417, R^2^=0.088, χ^2^=170.3, AUC=0.685, p=1.1E-36			
	HLA-DRB1*03	27.3	2.23 (1.65 to 3.02)	1.82E-07
	*C4L*=0+1+2	7.47	1.54 (1.13 to 2.11)	0.0062
	*C4A*=0+1	7.12	1.60 (1.13 to 2.26)	0.0076
JDM	n=3619, R^2^=0.031, χ^2^=38.1, AUC=0.632, p=5.4E-09			
	*C4L*=0+1+2	7.77	1.90 (1.22 to 2.95)	0.0053
	*C4A*=0+1	5.45	1.69 (1.08 to 2.63)	0.02
DM	n=4010, R^2^=0.033, χ^2^=105.0, AUC=0.682, p=1.6E-23			
	*C4A*=0+1	55.3	2.16 (1.77 to 2.64)	1.02E-13
	*C4S*=0	19.7	0.59 (0.46 to 0.75)	8.94E-06
PM	n=980, R^2^=0.095, χ^2^=121.5, AUC=0.632, p=3.7E-26			
	*C4A*=0+1	13.3	2.11 (1.42 to 3.14)	0.0003
	HLA-DRB1*03	19.4	2.46 (1.65 to 3.66)	1.05E-05
	HLA-DRB1*03**C4A=0+1	4.05		0.044
IBM	n=739, R^2^=0.1643, χ^2^=104.4, AUC=0.760, p=2.1E-23			
	HLA-DRB1*03	46.9	6.36 (3.69 to 11.0)	3.06E-11
	*C4T* GCN	4.49		0.034
**B. Predictors for myositis-related autoantibodies in patients with IIM**				
	**Predictors**	**χ** ^**2**^	**P value**	**OR (95% CI)**
MSA	n=684, χ^2^=21.6; R^2^=0.0236; AUC=0.597; p=7.8E-05			
	HLA-DRB1*03	11.4	0.0008	1.98 (1.33 to 2.95)
	*C4B* GCN	8.75	0.0036	
	*C4L* GCN	6.78	0.0099	
MSA-Jo1	n=688, χ^2^=76.9; R^2^=0.121; AUC=0.746; p=7.8E-16			
	*C4A*=0+1	24.3	8.30E-07	3.61 (2.11 to 6.19)
	C4P/G	23.2	1.50E-06	
	C3 protein	16	6.40E-05	
	HLA-DRB1*03	8.3	0.004	2.32 (1.29 to 4.18)
MAA	n=698, χ^2^=77.9; R^2^=0.101; AUC=0.714; p=2.3E-15			
	HLA-DRB1*15	12.2	0.0005	2.25 (1.44 to 3.53)
	HLA-DRB1*03	8.83	0.003	1.84 (1.22 to 2.76)
	C4 protein	9.31	0.0023	
	C3 protein	10.7	0.0011	
	C3**C4 protein	8.69	0.0032	
PM/Scl	n=692, χ^2^=53.2; R^2^=0.142; AUC=0.780; p=1.64E-11			
	HLA-DRB1*03	39	4.20E-10	21.7 (6.05 to 77.6)
	*C4A* GCN	7.39	0.0066	
	C4 protein	9.5	0.0021	
Ro	n=696, χ^2^=24.1, R^2^=0.0590; AUC=0.677; p=4.77E-05			
	HLA-DRB1*03	12	0.0011	2.83 (1.52 to 5.28)
	C4P/G	8.29	0.004	
	HLA-DRB1*15	8.27	0.0051	2.38 (1.30 to 4.38)

Double asterisks (**) between two predictors indicated interactions.

AUC, area under the curve; *C4A*, acidic isotype of complement C4; *C4B*, basic isotype of complement C4; *C4L*, long form of *C4* gene with human endogenous retrovirus HERV-K(C4); C4P/G, C4 protein per gene copy; *C4S*, short form of *C4* gene without integration of the retrovirus HERV-K(C4); *C4T*, total copy number of *C4* genes; DM, dermatomyositis; GCN, gene copy number; HLA, human leucocyte antigen; IBM, inclusion body myositis; IIM, idiopathic inflammatory myopathies; JDM, juvenile dermatomyositis; MAA, myositis-associated autoantibodies; MSA, myositis-specific autoantibodies; PM, polymyositis.

We performed logistic regression to investigate the relative roles of *C4A* deficiency and HLA-DRB1*03 as independent risk factors for IIM and subgroups. The results are shown in table 4. It was found that (1) *C4A* deficiency and *C4* gene size variation were independent risk predictors of JDM and DM and (2) HLA-DRB1*03 and *C4A* deficiency and GCN of *C4T* were independent risk factors for PM and IBM. Moreover, HLA-DRB1*03 and *C4A* deficiency interacted to increase the risk of PM. We also performed intragroup logistic regression analyses to identify independent predictors of IIM-related autoantibodies. Complement C4 or C3 protein or C4P/G, HLA-DRB1*03 and/or HLA-DRB1*15, *C4A* deficiency or *C4A* GCN range of variations were risk factors for various myositis-related autoantibodies except for MSA in general. For patients with MSA, genetic factors such as HLA-DRB1*03, GCNs of *C4B* and *C4L* were independent predictors.

## Discussion

Here we investigated complement *C4* genetic diversity in patients with IIM of European descent and matched healthy controls. Our data consistently showed that low copy numbers of *C4T* and *C4L*, and *C4A* deficiency are highly significant risk factors for IIM and its major subgroups, with medium to large effect sizes^45^ or ORs between 1.7 and 3.7. Compared with healthy controls, patients with IIM had 0.28 to 0.58 fewer mean gene copies of *C4T*, *C4A* or *C4L*. The *C4T*=2 group yielded similar risks as the *C4T*=2+3 group, and the *C4A*=0 group had similar risk as the *C4A*=0+1 group. The similar magnitudes of ORs suggested that there were ‘dominant’ effects for low GCN of total *C4* (ie, *C4T*=2 and *C4T*=2+3) and *C4A* deficiency (*C4A*=0 and *C4A*=0+1) on the risk of IIM, which is analogous to when homozygous and heterozygous mutants exhibit the same phenotype in Mendelian genetics. Such phenomena are in stark contrast to those observed in the genetics of human systemic lupus erythematosus (SLE), in which low GCNs of *C4T* or homozygous *C4A* deficiency (*C4T*=2, OR=6.51; *C4A*=0, OR=5.27) exerted substantially greater risks than those with *C4T*=3 (OR=1.32) or heterozygous *C4A* deficiency (*C4A*=1, OR=1.61).^23 24 46^ Parallel analyses of *C4* structural variants between cases and controls recruited from each geographic location yielded similar results as presented for the entire IIM cohort, which are analogous to replication studies (online supplemental table S1).

Complement-mediated destruction leading to vasculopathy in dermatomyositis has been well-established,^1 6 47 48^ and we and others have demonstrated *C4A* genetic deficiency or low GCN of *C4T* in JDM.^33 48^ Demonstration of low *C4T* or *C4L* GCNs and *C4A* deficiency as genetic risk factors for DM, PM and IBM are novel findings of this work. These findings are relevant, as PM and IBM have been presumed to be disorders of cell-mediated immunity caused by target tissue cytotoxicity or destruction.^1^ The prevalence of low GCNs of *C4T* and *C4L, C4A* deficiency, and the presence of myositis-related autoantibodies in these diseases suggests that additional *humoral* immune effectors play a role in the pathophysiology of PM and IBM. IBM is unique as it has low GCNs in *C4T*, *C4A* and *C4B*. In a study of anti-Ro/anti-La patients with autoimmune diseases including myositis, Lundtoft and colleagues observed low GCNs of *C4A* in Scandinavian patients.^49^

It is worthy pointing out that the effects of GCN variation for *C4S* and *C4B* were opposite to those of *C4L* or *C4A* in JDM, DM and PM, which suggests different functions of *C4S* and *C4B* compared with *C4L* and *C4A*. Indeed, short *C4* genes associate with higher C4 protein production^50 51^ and activated C4B protein generates faster activation of complement pathways^52^; long *C4* genes associate with attenuated C4 protein production but possibly engage in antisense defence against viral infections.^26 27 50^ Moreover, activated C4A has greater efficiency to bind to immune complexes for clearance and protection against autoimmunity.^26 27 50–53^ We postulate that activated C4A and C4B proteins interfere and balance each other’s effects physiologically to achieve optimum defence against infections and autoimmunity and mitigate collateral damages due to complement-mediated injuries of self-tissue. *C4A* deficiency, which is also indicated by low copy numbers of *C4T* or *C4L,* and higher proportion of *C4B* among total *C4* (*C4B/C4T*), would disturb such dynamic equilibrium and skew the immune response towards inflammation and autoimmunity with generation of autoantibodies.^25 32^

While IIM typically does *not* feature dramatic longitudinal fluctuations of plasma C3 and C4 protein levels with disease activity as is the case in SLE,^54^ intragroup analyses revealed that patients with IIM with anti-Jo-1, MAA in general, anti-PM/Scl or anti-Ro had significantly lower mean complement protein levels than those without these autoantibodies. Immune complexes formed by autoantibodies and self-antigens in IIM could activate and consume complement, leading to ‘depressed’ C4 and C3 plasma protein levels that were seen here and by others.^55^ Lower GCNs of *C4T*, *C4L* or *C4A* in IIM would be among the causes for lower C4 protein levels. Data on C4P/G yield, elevated levels of activation products in the plasma such as C4a, C3a and C5a, or cell-bound complement inactivation products such as erythrocyte-C4d and erythrocyte-C3d would help distinguish whether lower protein levels are due to genetic insufficiency or protein turnover.^33 56^ It is of interest to note that except for Jo-1, most MSA were not associated with lower complement levels in circulation, although MAA did. Moreover, patients with JDM have MSA such as anti-TIF1γ and anti-NXP2^4 57^ and their relationship with complement activation is yet to be investigated.

In a study of complement in schizophrenia, SLE and Sjogren’s syndrome, it was suggested that C4 exhibited a sex-biased expression differences including in cerebrospinal fluid.^58^ We did not detect differential expression of C4 protein in EDTA-plasma between men and women among patients with IIM in this work or in previous studies.^23 42 50 51 54^ We did not detect differences in *C4* GCN variations between female and male patients for DM, PM and JDM (online supplemental table S2). However, IBM is a male-dominant disease and we observed slightly higher frequencies of low GCNs for *C4L*, lower proportions of *C4A* or *C4L* among *C4T* in men compared with women.

The relative roles of HLA class II variants including DRB1*03, DQA1*05, DQB1*02 and *C4A* deficiency on genetic predisposition to autoimmune diseases such as IIM are an unsolved enigma.^17 47 59^ Multivariate logistic regression analyses revealed that *C4A* deficiency was an independent risk factor for DM and JDM and that HLA-DRB1*03 was a prominent risk factor for IBM, while *C4A* deficiency and HLA-DRB1*03 contributed independently and interactively to an increased risk of PM. Further analyses of *DRB1*, *DQA1*, *DQB1* variants and GCNs of *C4* revealed the presence of both risk and protective factors in each gene on the predisposition of IIM subgroups and autoantibodies (online supplemental figure S3 and online supplemental table S4).

In summary, our results demonstrated that low GCNs for *C4T*, *C4A* and *C4L* played significant roles in increasing the risk of IIM. The relationship between *C4A* deficiency and HLA-DRB1*03, which are closely linked, is complex and intriguing. It will be important going forward to carefully interrogate the mechanisms by which HLA-DRB1*03 and *C4A* deficiency contribute to autoimmunity and IIM. Finally, intragroup analyses showed that patients with IIM with certain autoantibodies presented with lower protein levels of complement C3 and C4. This effect was more notable for MAA than for MSA, which is worthy of investigations. Our findings have broad implications in the assessment and treatment of IIM and other autoimmune diseases.

## Data Availability

Data are available on reasonable request.
