# Supplementary material for: Low copy numbers of complement C4 and C4A deficiency are risk factors for myositis, its subgroups and autoantibodies
Source: Ann Rheum Dis. 2022 Sep 28;82(2):235–45. doi: 10.1136/ard-2022-222935 (PMC9887400; doi:10.1136/ard-2022-222935)
Supplement: Supplementary data [file ard-2022-222935supp001.pdf]

**SUPPLEMENTARY MATERIALS**

# Low Copy Numbers of Complement *C4* and *C4A* Deficiency Are Risk Factors for Myositis, Its Subgroups and Autoantibodies

Danlei Zhou,[1,2] Emily H. King,[1,2] Simon Rothwell,[3,4] Olga Kryštůfková,[5] Antonella Notarnicola,[6] Samantha Coss,[1,2] Rabheh Abdul-Aziz,[2,7] Katherine E. Miller,[1,2] Amanda Dang,[1] G. Richard Yu,[1] Joanne Drew,[2] Emeli Lundstrom,[6] Lauren M. Pachman,[8] Gulnara Mamyrova,[9] Rodolfo V. Curiel,[9] Boel De Paepe,[10] Jan L. De Bleecker,[10] Antony Payton,[11] William Ollier,[12] Terrance P. O'Hanlon,[13] Ira N. Targoff,[14] Willy A. Flegel,[15] Vidya Sivaraman,[2] Edward Oberle,[2] Shoghik Akoghlanian,[2] Kyla Driest,[2] Charles H. Spencer,[2] Yee Ling Wu,[2,16] Haikady N. Nagaraja,[17] Stacy P. Ardoin,[2] Hector Chinoy,[3,4] Lisa G. Rider,[13] Frederick W. Miller,[13] Ingrid E. Lundberg,[6] Leonid Padyukov,[6] Jiří Vencovský,[5] Janine A. Lamb[18] and Chack-Yung Yu[1,2] for MYOGEN Investigators

**Supplementary Data / Results**

**Table S1**. Comparisons of *C4* gene copy number groups between IIM and its subgroups DM, PM and IBM and matched controls from different geographic locations:

A. All sites
B. United Kingdom
C. Sweden
D. Central Europe

**Table S2**. Comparisons of structural variants for complement *C4* GCNs between female and male patients of IIM and controls (CTL)

**Table S3**. Contingency analyses of *HLA-DRB1*03* and complement *C4* gene copy number variations on myositis and subtypes

**Table S4**. Relationship between ten common HLA class II alleles and low gene copy number variants or deficiencies of complement C4 isoforms.

Limitations of this study

**Supplementary Figures**

1. **Fig. S1**. Copy number variations of total *C4*, *C4A*, *C4B*, long genes (*C4L*) and short genes (*C4S*) in healthy controls.
2. **Fig. S2**. Comparisons of GCN variations for total C4 (panel A) and C4A (panel B) between IIM patients (red) and matched controls (blue). Arrows in panel A indicate the directions of changes.
3. **Fig. S3.** Logistic regression models for myositis subgroups and myositis related autoantibodies: DM, JDM, PM, IBM, MSA, MSA-Jo-1, MAA, MAA-PM/Scl and MAA-Ro.

**Table S1.** Comparisons of *C4* gene copy number groups between IIM (and its major subgroups) and matched controls in different geographic locations[Δ]

A. <u>IIM patients and controls from all sites.</u>

| **All Sites, N** | ***C4T* GCN** | **2** | **3** | **4** | **≥5** | | | **$\chi^2$** | **p** |
|---|---|---|---|---|---|---|---|---|---|
| 3500 | CTL (%) | 3.29 | 26.51 | 55.89 | 14.31 | | | | |
| 1638 | IIM (%) | 7.14 | 45.48 | 38.22 | 9.16 | | | 248.5 | 1.37E-53 |
| 559 | DM (%) | 5.55 | 43.47 | 41.14 | 9.84 | | | 77.24 | 1.20E-16 |
| 666 | PM (%) | 8.56 | 46.10 | 37.24 | 8.11 | | | 153.1 | 5.61E-33 |
| 180 | IBM (%) | 8.89 | 52.22 | 28.89 | 10.00 | | | 73.49 | 7.64E-16 |
| | | | | | | | | | |
| | ***C4A* GCN** | **0** | **1** | **2** | **3** | **≥4** | | **$\chi^2$** | **p** |
| | CTL (%) | 1.74 | 19.26 | 51.30 | 22.75 | 4.94 | | | |
| | IIM (%) | 4.24 | 38.64 | 40.05 | 14.13 | 2.95 | | 263.90 | 6.51E-56 |
| | DM (%) | 3.95 | 36.62 | 42.37 | 14.72 | 2.33 | | 98.97 | 1.39E-09 |
| | PM (%) | 4.97 | 40.66 | 37.95 | 13.70 | 2.71 | | 168.5 | 2.17E-35 |
| | IBM (%) | 5.65 | 35.03 | 37.29 | 15.82 | 6.21 | | 37.89 | 1.76E-07 |
| | | | | | | | | | |
| | ***C4B* GCN** | **0** | **1** | **2** | **≥3** | | | **$\chi^2$** | **p** |
| | CTL (%) | 3.15 | 27.28 | 63.80 | 5.78 | | | | |
| | IIM (%) | 2.09 | 27.11 | 65.80 | 5.53 | | | 9.06 | 0.06 |
| | DM (%) | 1.81 | 25.27 | 66.06 | 6.86 | | | 5.32 | 0.15 |
| | PM (%) | 1.51 | 28.05 | 66.21 | 4.22 | | | 9.32 | 0.025 |
| | IBM (%) | 5.68 | 32.95 | 58.52 | 2.84 | | | 8.44 | 0.038 |
| | | | | | | | | | |
| | ***C4L* GCN** | **0** | **1** | **2** | **3** | **4** | **≥5** | **$\chi^2$** | **p** |
| | CTL (%) | 1.38 | 7.62 | 24.42 | 34.43 | 27.26 | 4.89 | | |
| | IIM (%) | 3.67 | 16.49 | 35.44 | 26.42 | 15.27 | 2.71 | 251.5 | 2.61E-52 |
| | DM (%) | 3.24 | 15.43 | 32.95 | 31.43 | 13.52 | 3.43 | 90.26 | 5.93E-18 |
| | PM (%) | 4.63 | 17.38 | 36.26 | 22.81 | 15.79 | 3.03 | 154.9 | 1.23E-31 |
| | IBM (%) | 4.09 | 15.79 | 39.18 | 22.81 | 16.96 | 1.17 | 49.87 | 1.48E-09 |
| | | | | | | | | | |
| | ***C4S* GCN** | **0** | **1** | **2** | **≥3** | | | **$\chi^2$** | **p** |
| | CTL (%) | 32.34 | 47.71 | 17.73 | 2.21 | | | | |
| | IIM (%) | 21.13 | 53.48 | 23.82 | 1.57 | | | 80.04 | 3.01E-17 |
| | DM (%) | 17.96 | 56.11 | 23.70 | 2.22 | | | 51.31 | 4.21E-11 |
| | PM (%) | 23.03 | 50.70 | 25.19 | 1.08 | | | 36.35 | 6.32E-08 |
| | IBM (%) | 22.73 | 56.82 | 19.32 | 1.14 | | | 9.41 | 0.024 |

[Δ] Note that JDM patients and controls were predominantly recruited in the US and therefore not segregated for analyses here. CTL, controls.

B. IIM patients and controls from the United Kingdom

| UK / Manchester, N | *C4T* GCN | 2 | 3 | 4 | ≥5 | | | $\chi^2$ | p |
|---|---|---|---|---|---|---|---|---|---|
| 1444** | CTL | 4.22 | 28.39 | 57.27 | 10.11 | | | | |
| 756 | IIM | 8.73 | 47.75 | 35.98 | 7.54 | | | 118.5 | 1.63E-25 |
| 256 | DM | 7.03 | 43.75 | 41.02 | 8.20 | | | 30.53 | 1.07E-06 |
| 361 | PM | 9.14 | 47.65 | 36.57 | 6.65 | | | 71.1 | 2.43E-15 |
| 115 | IBM | 13.04 | 54.78 | 24.35 | 7.83 | | | 58.9 | 9.97E-13 |
| | | | | | | | | | |
| | *C4A* GCN | 0 | 1 | 2 | 3 | ≥4 | | $\chi^2$ | p |
| | CTL | 2.01 | 21.65 | 51.77 | 20.61 | 3.96 | | | |
| | IIM | 5.20 | 41.07 | 36.80 | 14.93 | 2.00 | | 117.20 | 2.14E-24 |
| | DM | 5.14 | 39.13 | 37.94 | 16.60 | 1.19 | | 47.19 | 1.39E-09 |
| | PM | 4.74 | 41.23 | 37.33 | 14.76 | 1.95 | | 67.5 | 7.77E-14 |
| | IBM | 7.89 | 41.23 | 34.21 | 12.28 | 4.39 | | 35.78 | 3.21E-07 |
| | | | | | | | | | |
| | *C4B* GCN | 0 | 1 | 2 | ≥3 | | | $\chi^2$ | p |
| | CTL | 3.06 | 27.38 | 64.63 | 4.93 | | | | |
| | IIM | 2.00 | 27.43 | 64.95 | 5.59 | | | 2.58 | 0.460 |
| | DM | 1.58 | 25.30 | 64.43 | 8.70 | | | 7.13 | 0.068 |
| | PM | 1.67 | 29.17 | 64.72 | 4.44 | | | 2.74 | 0.43 |
| | IBM | 4.39 | 31.58 | 61.40 | 2.63 | | | 2.74 | 0.43 |
| | | | | | | | | | |
| | *C4L* GCN | 0 | 1 | 2 | 3 | 4 | ≥5 | $\chi^2$ | p |
| | CTL | 1.59 | 9.21 | 26.94 | 35.73 | 24.10 | 2.42 | | |
| | IIM | 3.99 | 21.11 | 37.38 | 22.40 | 13.12 | 2.00 | 133.4 | 4.72E-27 |
| | DM | 3.81 | 19.92 | 36.86 | 28.39 | 9.32 | 1.69 | 57.55 | 3.9E-11 |
| | PM | 3.90 | 22.22 | 37.24 | 18.62 | 15.32 | 2.70 | 86.58 | 3.51E-17 |
| | IBM | 5.50 | 19.27 | 39.45 | 20.18 | 14.68 | 0.92 | 32.90 | 3.97E-06 |
| | | | | | | | | | |
| | *C4S* GCN | 0 | 1 | 2 | ≥3 | | | $\chi^2$ | p |
| | CTL | 28.23 | 50.28 | 19.61 | 1.88 | | | | |
| | IIM | 18.96 | 52.66 | 27.01 | 1.36 | | | 30.41 | 1.13E-06 |
| | DM | 15.26 | 55.42 | 26.51 | 2.81 | | | 22.07 | 6.30E-05 |
| | PM | 22.13 | 47.70 | 29.31 | 0.86 | | | 17.77 | 0.0005 |
| | IBM | 18.75 | 60.71 | 20.54 | 0 | | | 9.99 | 0.019 |

** The UK controls include 150 historic healthy subjects from London.

C. IIM patients and controls from Sweden

| Sweden, N | *C4T* GCN | 2 | 3 | 4 | ≥5 | | | $\chi^2$ | p |
|---|---|---|---|---|---|---|---|---|---|
| 1017 | CTL | 3.35 | 24.43 | 50.34 | 21.87 | | | | |
| 381 | IIM | 5.26 | 45.00 | 37.37 | 12.37 | | | 62.93 | 1.39E-13 |
| 133 | DM | 5.30 | 46.21 | 39.39 | 9.09 | | | 33.05 | 3.14E-07 |
| 167 | PM | 6.59 | 45.51 | 35.33 | 12.57 | | | 37.52 | 3.58E-08 |
| 53 | IBM | 1.89 | 47.17 | 35.85 | 15.09 | | | 12.17 | 0.0068 |
| | | | | | | | | | |
| | *C4A* GCN* | 0 | 1 | 2 | 3 | ≥4 | | $\chi^2$ | p |
| | CTL | 2.26 | 17.01 | 45.62 | 27.73 | 7.37 | | | |
| | IIM | 2.96 | 30.97 | 44.09 | 16.27 | 5.77 | | 41.67 | 1.95E-08 |
| | DM | 2.26 | 32.33 | 45.11 | 17.29 | 3.01 | | 21.89 | 0.0002 |
| | PM | 3.59 | 34.13 | 41.32 | 14.97 | 5.99 | | 30.64 | 3.62E-06 |
| | IBM | 1.89 | 22.64 | 41.51 | 22.64 | 11.32 | | 2.48 | 0.65 |
| | | | | | | | | | |
| | *C4B* GCN* | 0 | 1 | 2 | ≥3 | | | $\chi^2$ | p |
| | CTL | 3.44 | 27.34 | 63.42 | 5.8 | | | | |
| | IIM | 2.65 | 28.84 | 65.87 | 2.65 | | | 7.35 | 0.062 |
| | DM | 2.29 | 26.72 | 67.94 | 3.05 | | | 2.76 | 0.43 |
| | PM | 2.40 | 28.14 | 66.47 | 2.99 | | | 3.19 | 0.36 |
| | IBM | 5.77 | 40.38 | 53.85 | 0 | | | 9.94 | 0.019 |
| | | | | | | | | | |
| | *C4L* GCN | 0 | 1 | 2 | 3 | 4 | ≥5 | $\chi^2$ | p |
| | CTL | 1.90 | 6.70 | 20.30 | 29.30 | 32.50 | 9.30 | | |
| | IIM | 4.04 | 12.13 | 38.01 | 29.65 | 15.36 | 0.83 | 116.7 | 1.60E-23 |
| | DM | 3.13 | 15.63 | 37.50 | 31.25 | 11.72 | 0.78 | 59.39 | 1.63E-11 |
| | PM | 5.52 | 11.66 | 36.81 | 29.45 | 15.95 | 0.61 | 60.57 | 9.29E-12 |
| | IBM | 1.92 | 9.62 | 36.54 | 28.85 | 21.15 | 1.92 | 12.29 | 0.031 |
| | | | | | | | | | |
| | *C4S* GCN | 0 | 1 | 2 | ≥3 | | | $\chi^2$ | p |
| | CTL | 38.02 | 44.81 | 15.57 | 1.60 | | | | |
| | IIM | 22.40 | 55.47 | 20.00 | 2.13 | | | 31.27 | 7.45E-07 |
| | DM | 16.41 | 59.38 | 21.88 | 2.34 | | | 25.91 | 1.00E-05 |
| | PM | 24.10 | 56.02 | 18.07 | 1.81 | | | 12.76 | 0.0052 |
| | IBM | 30.19 | 50.94 | 16.98 | 1.89 | | | 1.37 | 0.71 |

D. IIM patients and controls from Central Europe

| **Central Europe, N** [¶] | ***C4T* GCN** | **2** | **3** | **4** | **≥5** | | | **$\chi^2$** | **p** |
|---|---|---|---|---|---|---|---|---|---|
| 221 | CTL | 1.81 | 21.27 | 64.25 | 12.67 | | | | |
| 365 | IIM | 5.79 | 41.05 | 43.80 | 9.37 | | | 35.1 | 1.15E-07 |
| 165 | DM | 3.05 | 39.02 | 45.12 | 12.8 | | | 17.03 | 0.0007 |
| 134 | PM | 10.45 | 44.78 | 38.06 | 6.72 | | | 40.82 | 7.13E-09 |
| 12 | IBM | 0 | 50.00 | 41.67 | 8.33 | | | 4.79 | 0.19 |
| | | | | | | | | | |
| | ***C4A*** | **0** | **1** | **2** | **3** | **≥4** | | **$\chi^2$** | **p** |
| | CTL | 1.36 | 19.46 | 57.92 | 18.55 | 2.71 | | | |
| | IIM | 4.47 | 41.34 | 42.46 | 10.06 | 1.68 | | 40.48 | 3.45E-08 |
| | DM | 3.66 | 35.37 | 47.56 | 10.37 | 3.05 | | 17.37 | 0.0016 |
| | PM | 7.46 | 47.76 | 34.33 | 9.7 | 0.75 | | 46.02 | 2.44E-09 |
| | IBM | 0 | 30.00 | 50.00 | 20.00 | 0 | | 1.38 | 0.85 |
| | | | | | | | | | |
| | *C4B* | 0 | 1 | 2 | ≥3 | | | **$\chi^2$** | **p** |
| | CTL | 0.45 | 21.72 | 70.14 | 7.69 | | | | |
| | IIM | 1.13 | 24.23 | 68.45 | 6.2 | | | 1.65 | 0.65 |
| | DM | 1.23 | 23.31 | 68.1 | 7.36 | | | 0.889 | 0.83 |
| | PM | 0 | 25.76 | 71.21 | 3.03 | | | 4.86 | 0.18 |
| | IBM | 20.00 | 10.00 | 50.00 | 20.00 | | | 11.49 | 0.0094 |
| | | | | | | | | | |
| | C4L | 0 | 1 | 2 | 3 | 4 | ≥5 | **$\chi^2$** | **p** |
| | CTL | 0.91 | 8.64 | 17.73 | 33.18 | 33.18 | 3.36 | | |
| | IIM | 3.13 | 10.51 | 27.56 | 32.39 | 19.6 | 6.82 | 19.35 | 0.0016 |
| | DM | 2.52 | 8.18 | 22.01 | 37.11 | 22.01 | 8.18 | 7.35 | 0.20 |
| | PM | 5.34 | 12.98 | 32.82 | 25.19 | 17.56 | 6.11 | 24.93 | 0.0001 |
| | IBM | 0 | 10.00 | 50.00 | 20.00 | 20.00 | 0 | 6.28 | 0.28 |
| | | | | | | | | | |
| | C4S | 0 | 1 | 2 | ≥3 | | | **$\chi^2$** | **p** |
| | CTL | 35.62 | 42.47 | 18.72 | 3.2 | | | | |
| | IIM | 25.57 | 52.59 | 20.69 | 1.15 | | | 10.45 | |
| | DM | 23.72 | 53.21 | 21.79 | 1.28 | | | 8.47 | 0.037 |
| | PM | 24.03 | 52.71 | 22.48 | 0.78 | | | 8.43 | 0.038 |
| | IBM | 27.27 | 45.45 | 18.18 | 9.09 | | | 0.99 | 0.80 |

[¶] The Central Europe study population include 329 IIM patients and 96 controls from Czech Republic, 36 IIM patients from Belgium, and 125 historic healthy controls from Hungary (ref. 40) for these analyses.

**Table S2**. Comparisons of complement C4 GCNs between females and males of IIM patients and controls (CTL)

| A. Numeric data | | | | | | | | | | | |
|---|---|---|---|---|---|---|---|---|---|---|---|
| | | **Females** | | | | **Males** | | | | δ | remarks (males) |
| | | **N** | **Mean** | **SD** | **P** | **N** | **Mean** | **SD** | **P** | | |
| ***C4T*** | CTL | 2370 | 3.84 | 0.77 | | 883 | 3.83 | 0.76 | | 0.004 | |
| | IBM | 75 | 3.47 | 0.68 | 4.55E-05 | 105 | 3.35 | 0.86 | 2.38E-09 | 0.115 | lower C4T, p=0.34 |
| | IIM- | 1004 | 3.49 | 0.76 | 2.62E-33 | 433 | 3.55 | 0.79 | 9.24E-10 | -0.069 | |
| ***C4A*** | CTL | 2367 | 2.11 | 0.84 | | 885 | 2.11 | 0.84 | | 0.001 | |
| | IBM | 74 | 1.92 | 0.86 | 0.056 | 103 | 1.75 | 1.06 | 6.83E-05 | 0.171 | lower C4A, p=0.25 |
| | IIM- | 1001 | 1.71 | 0.85 | 1.74E-34 | 430 | 1.74 | 0.91 | 6.78E-13 | -0.026 | |
| ***C4B*** | CTL | 2365 | 1.72 | 0.63 | | 885 | 1.72 | 0.63 | | 0.000 | |
| | IBM | 74 | 1.55 | 0.71 | 0.023 | 102 | 1.61 | 0.60 | 0.081 | -0.054 | |
| | IIM- | 999 | 1.74 | 0.56 | 0.47 | 428 | 1.79 | 0.61 | 0.060 | -0.052 | |
| ***C4L*** | CTL | 2355 | 2.93 | 1.08 | | 880 | 2.94 | 1.08 | | -0.007 | |
| | IBM | 71 | 2.55 | 0.92 | 0.0031 | 100 | 2.23 | 1.20 | 1.04E-09 | 0.319 | lower C4L, p=0.061 |
| | IIM- | 949 | 2.40 | 1.13 | 4.40E-36 | 411 | 2.47 | 1.14 | 1.15E-12 | -0.072 | |
| ***C4S*** | CTL | 2351 | 0.90 | 0.76 | | 880 | 0.89 | 0.79 | | 0.009 | |
| | IBM | 72 | 0.93 | 0.66 | 0.74 | 104 | 1.03 | 0.70 | 0.087 | -0.098 | |
| | IIM- | 980 | 1.06 | 0.72 | 8.38E-09 | 419 | 1.08 | 0.74 | 4.80E-05 | -0.012 | |
| ***C4L/T*** | CTL | 2375 | 0.74 | 0.23 | | 886 | 0.75 | 0.23 | | -0.007 | |
| | IBM | 75 | 0.69 | 0.25 | 0.041 | 105 | 0.60 | 0.29 | 2.11E-09 | 0.087 | lower C4L/C4T, p=0.041 |
| | IIM- | 1002 | 0.63 | 0.29 | 9.28E-35 | 433 | 0.64 | 0.28 | 1.70E-14 | -0.010 | |
| ***C4A/T*** | CTL | 2367 | 0.54 | 0.18 | | 886 | 0.54 | 0.18 | | -0.003 | |
| | IBM | 74 | 0.55 | 0.22 | 0.75 | 103 | 0.49 | 0.23 | 0.0066 | 0.056 | lower C4A/C4T, p=0.103 |
| | IIM- | 1000 | 0.48 | 0.19 | 2.96E-19 | 430 | 0.47 | 0.20 | 3.59E-10 | 0.004 | |
| **B. Categorical data** | | | | | | | | | | | |
| | | **%** | **OR** | **95%CI** | **P** | **%** | **OR** | **95%CI** | **P** | δ | |
| ***C4T*=2+3** | CTL | 30.1 | | | | 29.5 | | | | 0.630 | |
| | IBM | 56.0 | 2.96 | 1.86-4.71 | 4.85E-06 | 64.8 | 4.40 | 2.88-6.74 | 2.23E-12 | -8.760 | higher OR C4T |
| | IIM- | 52.6 | 2.58 | 2.21-3.00 | 1.28E-34 | 50.4 | 2.43 | 1.92-3.08 | 2.00E-13 | 2.290 | |
| ***C4A*=0+1** | CTL | 21.0 | | | | 20.7 | | | | 0.270 | |
| | IBM | 32.4 | 1.81 | 1.10-2.98 | 0.024 | 46.6 | 3.35 | 2.20-5.10 | 3.88E-08 | -14.170 | higher C4A def, OR; p=0.057 |
| | IIM- | 43.3 | 2.86 | 2.44-3.36 | 4.57E-39 | 43.7 | 2.99 | 2.33-3.85 | 8.12E-18 | -0.460 | |
| ***C4B*=0+1** | CTL | 30.6 | | | | 30.4 | | | | 0.210 | |
| | IBM | 40.5 | 1.55 | 0.96-2.48 | 0.075 | 37.3 | 1.36 | 0.89-2.08 | 0.162 | 3.290 | |
| | IIM- | 29.1 | 0.93 | 0.79-1.10 | 0.40 | 25.9 | 0.80 | 0.62-1.04 | 0.092 | 3.200 | |
| ***C4L*=0+1+2** | CTL | 33.6 | | | | 33.1 | | | | 0.41 | |
| | IBM | 50.7 | 2.04 | 1.27-3.27 | 0.0034 | 65.0 | 3.75 | 2.43-5.78 | 8.43E-10 | -14.3 | higher C4L=0+1+2 and OR; p=0.061 |
| | IIM- | 55.6 | 2.47 | 2.12-2.88 | 3.81E-31 | 54.3 | 2.39 | 1.88-3.04 | 6.63E-13 | 1.32 | |
| ***C4S*=0** | CTL | 32.0 | | | | 34.1 | | | | -2.08 | |
| | IBM | 23.6 | 0.66 | 0.38-1.14 | 0.123 | 22.1 | 0.55 | 0.34-0.89 | 0.011 | 1.49 | |
| | IIM- | 20.8 | 0.56 | 0.47-0.67 | 4.43E-11 | 21.0 | 0.52 | 0.39-0.68 | 9.52E-07 | -0.16 | |

| | | | | | | | | | | | | |
|---|---|---|---|---|---|---|---|---|---|---|---|---|
| **HLA-*DRB1*03*** | CTL | 25.7 | | | | | 27.9 | | | | -2.22 | |
| | IBM | 76.0 | 9.18 | 4.66-18.1 | 1.86E-12 | | 75.0 | 7.77 | 3.89-15.5 | 4.49E-10 | 1 | |
| | IIM- | 55.6 | 3.62 | 2.78-4.73 | 1.79E-22 | | 49.8 | 2.57 | 1.60-4.11 | 5.29E-05 | 5.77 | |

* IIM- : IIM with IBM excluded, i.e., DM, PM and JDM only; OR, odds ratio; p, p-values when compared with controls (CTL). Except for IBM, no significant differences in C4-GCNs were observed between male and female patients with IIM (DM, PM and JDM)

**Table S3**. Contingency analyses of *HLA-DRB1*03* and complement *C4* gene copy number variations on myositis and subtypes

| | | odds ratio (95% CI) | p |
|---|---|---|---|
| | ***HLA-DRB1*03*, %** | | |
| CTL | 26.1 | | |
| IIM | 56.1 | 3.68 (2.94-4.60) | 2.6E-32 |
| JDM | 45.5 | 2.36 (1.56-3.79) | 6.5E-05 |
| DM | 47.6 | 2.57 (1.90-3.49) | 1.1E-05 |
| PM | 59.5 | 4.16 (3.15-5.48) | 3.9E-25 |
| IBM | 75.4 | 8.71 (5.48-13.8) | 1.6E-23 |
| | | | |
| | ***C4A*=0+1, %** | | |
| CTL | 21.0 | | |
| IIM | 42.9 | 2.82 (2.48-3.21) | 3.5E-57 |
| JDM | 40.4 | 2.54 (1.82-3.56) | 1.4E-07 |
| DM | 40.6 | 2.57 (2.13-3.10) | 8.7E-22 |
| PM | 45.6 | 3.15 (2.65-3.75) | 2.2E-37 |
| IBM | 40.7 | 2.57 (1.89-3.52) | 8.9E-09 |
| | | | |
| | ***C4L*=0+1+2, %** | | |
| CTL | 33.4 | | |
| IIM | 55.6 | 2.49 (2.20-2.82) | 5.2E-49 |
| JDM | 56.1 | 2.54 (1.83-3.54) | 3.3E-08 |
| DM | 51.6 | 2.13 (1.77-2.56) | 1.7E-15 |
| PM | 58.4 | 2.79 (2.35-3.32) | 1.2E-31 |
| IBM | 59.1 | 2.87 (2.10-3.93) | 2.4E-11 |
| | | | |
| *Contingency analyses* | | | |
| | | | |
| ***C4A*=0+1, Yes** | ***HLA-DRB1*03*, %** | | |
| CTL | 83.1 | | |
| IIM | 88.6 | 1.59 (0.89-2.81) | 0.12 |
| JDM | 90.9 | 2.04 (0.66-6.35) | 0.19 |
| DM | 82.0 | 0.93 (0.47-1.84) | 0.83 |
| PM | 88.9 | 1.63 (0.82-3.24) | 0.16 |
| IBM | 98.2 | 11.02 (1.44-84.4) | 0.0012 |
| ***C4A*=0+1, No** | ***HLA-DRB1*03*, %** | | |
| CTL | 12.8 | | |
| IIM | 30.6 | 3.00 (2.16-4.15) | 1.1E-11 |
| JDM | 15.9 | 1.28 (0.62-2.65) | 0.51 |
| DM | 20.4 | 1.75 (1.07-2.85) | 0.03 |
| PM | 35.2 | 3.70 (2.50-5.48) | 9.5E-11 |
| IBM | 55.2 | 8.37 (4.69-14.94) | 1.5E-12 |
| | | | |
| ***C4L*=0+1+2, Yes** | ***HLA-DRB1*03*, %** | | |
| CTL | 61.5 | | |

| | | | |
|---|---|---|---|
| IIM | 81.5 | 2.77 (1.94-3.98) | 3.4E-08 |
| JDM | 73.4 | 1.72 (0.93-3.21) | 0.077 |
| DM | 77.1 | 2.11 (1.30-3.42) | 0.002 |
| PM | 81.3 | 2.71 (1.73-4.23) | 7.4E-06 |
| IBM | 97.1 | 20.94 (4.99-87.8) | 1.3E-10 |
| ***C4L*=0+1+2, No** | ***HLA-DRB1*03*, %** | | |
| CTL | 8.4 | | |
| IIM | 17.5 | 2.31 (1.47-3.65) | 0.0003 |
| JDM | 7.0 | 0.82 (0.24-2.78) | 0.74 |
| DM | 7.7 | 0.91 (0.39-2.12) | 0.82 |
| PM | 21.5 | 2.99 (1.71-5.20) | 0.0002 |
| IBM | 35.9 | 6.11 (2.92-12.81) | 8.6E-06 |
| | | | |
| ***HLA-DRB1*03*, Yes** | ***C4A*=0+1, %** | | |
| CTL | 60.1 | | |
| IIM | 70.8 | 1.61 (1.11-2.33) | 0.012 |
| JDM | 80.0 | 2.65 (1.24-5.68) | 0.0078 |
| DM | 76.5 | 2.15 (1.27-3.65) | 0.0035 |
| PM | 67.9 | 1.40 (0.91-2.15) | 0.12 |
| IBM | 65.8 | 1.12 (0.65-1.92) | 0.68 |
| ***HLA-DRB1*03*, No** | ***C4A*=0+1, %** | | |
| CTL | 4.3 | | |
| IIM | 12.1 | 3.04 (1.75-5.26) | 3.9E-05 |
| JDM | 7.0 | 1.67 (0.55-5.06) | 0.39 |
| DM | 15.5 | 4.05 (2.11-7.80) | 4.5E-05 |
| PM | 12.6 | 3.18 (1.63-6.20) | 0.0009 |
| IBM | 3.7 | 0.85 (0.11-6.58) | 0.87 |
| | | | |
| ***HLA-DRB1*03*,Yes** | ***C4L*=0+1+2, %** | | |
| CTL | 78.5 | | |
| IIM | 88.4 | 2.08 (1.30-3.33) | 0.0028 |
| JDM | 94.0 | 4.28 (1.25-15.6) | 0.0061 |
| DM | 93.9 | 4.21 (1.80-9.88) | 0.0002 |
| PM | 86.7 | 1.78 (1.02-3.10) | 0.042 |
| IBM | 82.7 | 1.31 (0.66-2.60) | 0.44 |
| ***HLA-DRB1*03*, No** | ***C4L*=0+1+2, %** | | |
| CTL | 17.3 | | |
| IIM | 26.7 | 1.74 (1.24-2.44) | 0.0014 |
| JDM | 29.8 | 2.03 (1.10-3.76) | 0.03 |
| DM | 27.6 | 1.82 (1.13-2.92) | 0.016 |
| PM | 29.1 | 1.96 1.25-3.06) | 0.0036 |
| IBM | 7.4 | 0.38 (0.09-1.65) | 0.14 |
| | | | |
| ***HLA-DRB1*03*, Yes** | ***C4B*=0+1, %** | | |
| CTL | 30.7 | | |
| IIM | 23.8 | 0.71 (0.48-1.05) | 0.086 |

| | | | |
|---|---|---|---|
| JDM | 16.0 | 0.43 (0.19-0.98) | **0.034** |
| DM | 21.0 | 0.60 (0.35-1.04) | 0.067 |
| PM | 25.9 | 0.79 (0.50-1.25) | 0.31 |
| IBM | 31.4 | 1.03 (0.59-1.82) | 0.91 |
| ***HLA-DRB1*03*, No** | ***C4B*=0+1, %** | | |
| CTL | 31.4 | | |
| IIM | 37.0 | 1.28 (0.96-1.71) | 0.091 |
| JDM | 40.4 | 1.47 (0.84-2.60) | 0.18 |
| DM | 32.6 | 1.05 (0.69-1.60) | 0.80 |
| PM | 38.2 | 1.35 (0.92-1.99) | 0.13 |
| IBM | 51.9 | 2.35 (1.08-5.14) | 0.033 |

***Table S3. Contingency analyses: HLA-DRB1*03* and complement *C4* GCNs in IIM and healthy controls**.

Effects of *DRB1*03*-positivity under the presence and absence of *C4A* deficiency, or low copy number of long *C4* genes. We dissected the relative roles of *HLA-DRB1*03*, *C4A* deficiency (*C4A*=0+1), low GCN of *C4L (C4L=0+1+2)*, and *C4B* deficiency (*C4B*=0+1) on conferring genetic risk of IIM and its subgroups by contingency analyses.

First, IIM patients were segregated into groups based on *C4A* deficiency and the effects of *HLA-DRB1*03* was investigated. In the *presence* of *C4A* deficiency, *DRB1*03* did *not* have a significant impact on increasing the risks of JDM, DM and PM but the influence of *DRB1*03* on IBM was substantial (Table 4). Strikingly, 98.2 % of IBM patients with *C4A* deficiency had *HLA-DRB1*03* with an OR of 11.0 (1.44-84.4; p=0.0012). In the *absence* of *C4A* deficiency, *DRB1*03*-positivity became relevant for DM [OR=1.75 (1.07-2.85)] and PM [OR=3.70 (2.50-5.48)], but not for JDM. The greatest effect of *DRB1*03* was again found in patients with IBM [55.2% vs 12.8%; OR=8.37 (4.69-14.94); p=$1.5x10^{-12}$].

Second, we asked if *DRB1*03* was a significant risk factor when IIM patients were segregated based on *C4* gene length. Among patients with *C4L*=0+1+2, *DRB1*03* was a prominent risk factor for IBM as 97.1% had *DRB1*03* with an OR of 20.9 (4.99-87.8; p=$1.3x10^{-10}$). Under the same conditions, *DRB1*03*-positivity was a moderate risk factor for DM and PM with ORs of 2.11 (1.30-3.42) and 2.71 (1.73-4.23), respectively. The impact of *DRB1*03* on JDM was marginal [OR=1.72 (0.93-3.21); p, not significant]. In the absence of low *C4L*, *DRB1*03* was not significantly associated with increased risk of DM and JDM, though it was moderate risk factor for PM [OR=2.99 (1.71-5.20), p=0.0002] and a large effect size risk factor for IBM [OR=6.11 (2.92-12.81), p=$8.6x10^{-6}$].

Effects of *C4A* deficiency and low GCNs of *C4L* under the presence and absence of *HLA-DRB1*03*. IIM patients were then segregated based on *HLA-DRB1*03* status and we asked if *C4A* deficiency or low GCN of *C4L* were significant on increasing the risk of IIM and its subgroups. In the presence of *DRB1*03*, *C4A* deficiency was a significant risk factor for JDM and DM with OR of 2.65 (1.24-5.68) and 2.15 (1.27-3.65), respectively. The same phenomenon did not hold for PM and IBM. In the absence of *DRB1*03*, *C4A* deficiency was a highly significant risk factor for DM (p=$4.5x10^{-5}$) and PM (p=0.0009) with ORs of 4.05 (2.11-7.80) and 3.18 (1.63-6.20), respectively. However, *C4A* deficiency did not contribute significantly to JDM and IBM risk.

Similar contingency analyses were performed for IIM patients with and without low *C4L* GCN in the setting of defined *DRB1*03* backgrounds. The results were similar to those observed for *C4A* deficiency and

details are shown in Table 3. Both *C4A* deficiency and low copy number of *C4L* were not significant risk factors for IBM, irrespective of the status of *DRB1*03*.

Parallel contingency analyses revealed that when *DRB1*03* was present, *C4B* deficiency was a *protective* factor for JDM [OR=0.43 (0.19-0.98)]. When *DRB1*03* was absent, *C4B* deficiency was a *risk* factor for IBM, which had an OR=2.35 (1.08-5.14) (Table S3).

This is an important advance for the alleles of complement *C4* and *HLA-DRB1* on the risk of an autoimmune disease.

We determined complement C4 and C3 plasma protein levels using EDTA-plasma from IIM patients. Each additional *C4* gene copy increased mean C4 plasma protein levels by 66.9 mg/L. The regression formula was C4 protein (mg/L) = 59.1 + 66.9*GCN of *C4T*. The *net* yield of plasma C4 protein per gene copy (C4P/G) was slightly reduced for *C4T* or long genes with increases of GCN [regression formulae: C4P/G (mg/L) =104.6 - 5.6*GCN of *C4T*; or C4P/G = 108.4 - 8.9*GCN of *C4L*]. It is worthy to point out that *C4* GCN variations are integral determining factors for plasma C4 proteins, and C3 is downstream of C4 in two complement activation pathways.

**Table S4. Relationship between ten common HLA class II alleles and low gene copy number variants or deficiencies of complement C4 isoforms.**

A. HLA with total C4 (C4T)

| | | C4T=2+3 % (No., Y/N) | OR* | P | |
|---|---|---|---|---|---|
| DR1 | | | | | |
| CTL | N | 32.6 (459/950) | | | |
| | Y | 29.4 (123/296) | 0.86 (.68-1.09) | 0.21 | |
| IIM | N | 58.5 (385/273) | | | |
| | Y | 46.3 (93/108) | 0.61 (.44-.84) | 0.0023 | |
| DR2/15 | | | | | |
| CTL | N | 34.4 (458/872) | | | |
| | Y | 25.0 (124/373) | 0.63 (.50-.80) | 8.50E-05 | |
| IIM | N | 57.3 (403/300) | | | |
| | Y | 47.4 (74/82) | 0.67 (.47-.95) | 0.025 | |
| DR3 | | | | | |
| CTL | N | 12.9 (172/1163) | | | |
| | Y | 93.2 (410/83) | 33.4 (25.1-44.4) | 3.50E-179 | |
| IIM | N | 18.9 (75/302) | | | |
| | Y | 83.4 (403/80) | 20.3 (14.3-28.7) | 8.20E-83 | |
| DR4 | | | | | |
| CTL | N | 36.2 (440/777) | | | |
| | Y | 23.2 (142/469) | 0.53 (.43-.67) | 1.40E-08 | |
| IIM | N | 59.9 (376/252) | | | |
| | Y | 43.9 (101/129) | 0.52 (.39-.71) | 3.20E-05 | |
| DR7 | | | | | |
| CTL | N | 36.2 (484/855) | | | |
| | Y | 20.1 (98/390) | 0.44 (.34-.57) | 2.00E-11 | |
| IIM | N | 59.1 (413/286) | | | |
| | Y | 40.9 (63/91) | 0.48 (.34-.68) | 4.10E-05 | |
| DR13 | | | | | |
| CTL | N | 35.4 (516/941) | | | |
| | Y | 17.8 (66/305) | 0.40 (.30-.53) | 1.20E-11 | |
| IIM | N | 57.2 (414/310) | | | |
| | Y | 46.7 (63/72) | 0.66 (.45-.94) | 0.024 | |
| DQA1*05 | | | | | |
| CTL | N | 13.1 (93/616) | | | |
| | Y | 61.7 (305/189) | 10.7 (8.05-14.2) | 2.50E-71 | |
| IIM | N | 21.2 (57/212) | | | |
| | Y | 73.6 (395/142) | 10.3 (7.29-14.7) | 5.70E-47 | |
| DQA1*02 | | | | | |
| CTL | N | 37.0 (323/549) | | | |
| | Y | 22.7 (75/256) | 0.50 (.37-.67) | 1.30E-06 | |
| IIM | N | 59.9 (395/264) | | | |
| | Y | 38.8 (57/90) | 0.42 (.29-.61) | 3.10E-06 | |

| | | C4T=2+3<br>% (No., Y/N) | OR | P | |
|---|---|---|---|---|---|
| DQB1*02 | | | | | |
| CTL | N | 13.9 (93/574) | | | |
| | Y | 56.9 (305/231) | 8.15 (6.17-10.8) | 1.60E-57 | |
| IIM | N | 21.2 (58/216) | | | |
| | Y | 74.1 (392/137) | 10.7 (7.52-15.1) | 2.50E-48 | |
| DQB1*03 | | | | | |
| CTL | N | 45.6 (249/297) | | | |
| | Y | 22.7 (149/508) | 0.35 (.27-.45) | 3.40E-17 | |
| IIM | N | 66.3 (295/150) | | | |
| | Y | 43.2 (155/204) | 0.39 (.29-.51) | 4.60E-11 | |
| | | | | | |

B. HLA with deficiencies of C4A or C4B

| | | C4A=0+1<br>% (no., Y/N) | OR | P | | C4B=0+1<br>% (no., Y/N) | OR | P |
|---|---|---|---|---|---|---|---|---|
| DR1 | | | | | | | | |
| CTL | N | 25.1 (353/1056) | | | | 26.8 (377/1032) | | |
| | Y | 13.6 (57/362) | 0.47 (.35-.64) | 2.70E-07 | | 43.0 (180/239) | 2.06 (1.64-2.59) | 5.70E-10 |
| IIM | N | 49.8 (325/328) | | | | 25.1 (164/489) | | |
| | Y | 30.5 (61/139) | 0.44 (.32-.62) | 1.20E-06 | | 43.8 (88/113) | 2.32 (1.67-3.23) | 7.20E-07 |
| DR2/15 | | | | | | | | |
| CTL | N | 24.1 (320/1010) | | | | 32.9 (438/892) | | |
| | Y | 18.1 (90/407) | 0.70 (.54-.91) | 0.0058 | | 23.7 (118/379) | 0.63 (.50-.80) | 0.0001 |
| IIM | N | 46.1 (322/377) | | | | 30.6 (214/485) | | |
| | Y | 40.9 (63/91) | 0.81 (.57-1.15) | 0.24 | | 25.2 (39/116) | 0.76 (.51-1.13) | 0.17 |
| DR3 | | | | | | | | |
| CTL | N | 5.32 (71/1264) | | | | 32.1 (429/906) | | |
| | Y | 68.8 (339/154) | 39.2 (28.9-53.2) | 2.0E-171 | | 26.0 (128/365) | 0.74 (.59-.93) | 0.010 |
| IIM | N | 12.3 (46/327) | | | | 37.4 (140/234) | | |
| | Y | 70.7 (340/141) | 17.1 (11.9-24.7) | 1.40E-70 | | 23.5 (113/368) | 0.51 (.38-.69) | 9.80E-06 |
| DR4 | | | | | | | | |
| CTL | N | 27.5 (335/882) | | | | 24.2 (295/922) | | |
| | Y | 12.3 (75/536) | 0.37 (.28-.48) | 1.80E-14 | | 42.9 (262/349) | 2.35 (1.91-2.89) | 7.20E-16 |
| IIM | N | 49.5 (309/315) | | | | 26.2 (164/461) | | |
| | Y | 33.3 (76/152) | 0.51 (.37-.70) | 2.20E-05 | | 38.6 (88/140) | 1.77 (1.28-2.43) | 0.0006 |
| DR7 | | | | | | | | |
| CTL | N | 24.4 (327/1012) | | | | 34.4 461/878) | | |
| | Y | 17.0 (83/405) | 0.63 (.49-.83) | 0.0006 | | 19.5 (95/393) | 0.46 (.36-.59) | 2.50E-10 |
| IIM | N | 48.0 (333/361) | | | | 30.9 (215/480) | | |
| | Y | 33.3 (51/102) | 0.54 (.38-.78) | 0.0009 | | 24.2 (37/116) | 0.71 (.48-1.07) | 0.093 |
| DR13 | | | | | | | | |
| CTL | N | 24.9 (363/1094) | | | | 30.4 (443/1014) | | |
| | Y | 12.7 (47/324) | 0.44 (.31-.61) | 1.10E-07 | | 30.7 (114/257) | 1.01 (0.79-1.30) | 0.90 |
| IIM | N | 46.2 (332/387) | | | | 29.7 (214/506) | | |
| | Y | 39.6 (53/81) | 0.76 (.52-1.11) | 0.16 | | 29.1 (39/95) | 0.97 (.64-1.46) | 0.89 |
| DQA1*05 | | | | | | | | |
| CTL | N | 5.64 (40/669) | | | | 34.3 (243/466) | | |
| | Y | 51.0 (252/242) | 17.4 (12.1-25.1) | 3.70E-76 | | 24.1 (119/375) | 0.61 (.47-.79) | 0.0001 |
| IIM | N | 14.7 (39/226) | | | | 36.8 (98/168) | | |
| | Y | 60.6 (324/211) | 8.90 (6.08-13.0) | 2.30E-37 | | 26.4 (141/394) | 0.61 (.45-.84) | 0.0025 |
| DQA1*02 | | | | | | | | |
| CTL | N | 26.3 (229/643) | | | | 34.2 (298/574) | | |
| | Y | 19.0 (63/268) | 0.66 (.48-.90) | 0.0079 | | 19.3 (64/267) | 0.46 (.34-.63) | 2.60E-07 |
| IIM | N | 48.5 (317/337) | | | | 31.1 (204/451) | | |
| | Y | 31.5 (46/100) | 0.49 (.33-.72) | 0.0002 | | 24.0 (35/111) | 0.70 (.46-1.06) | 0.082 |

| | | C4A=0+1<br>% (no., Y/N) | OR | P | | C4B=0+1<br>% (no., Y/N) | OR | P |
|---|---|---|---|---|---|---|---|---|
| DQB1*02 | | | | | | | | |
| CTL | N | 5.85 (39/628) | | | | 35.7 (238/429) | | |
| | Y | 47.2 (253/283) | 14.4 (10.0-20.7) | 4.30E-66 | | 23.1 (124/412) | 0.54 (.42-.70) | 2.00E-06 |
| IIM | N | 13.3 (36/235) | | | | 37.9 (103/169) | | |
| | Y | 61.8 (325/201) | 10.6 (7.1-15.6) | 2.60E-42 | | 25.5 (134/392) | 0.56 (.41-.77) | 0.0003 |
| DQB1*03 | | | | | | | | |
| CTL | N | 35.5 (194/352) | | | | 26.0 (142/404) | | |
| | Y | 14.9 (98/559) | 0.32 (.24-.42) | 8.30E-17 | | 33.5 (220/437) | 1.43 (1.11-1.84) | 0.0047 |
| IIM | N | 55.2 (244/198) | | | | 27.8 (123/320) | | |
| | Y | 32.9 (117/239) | 0.40 (.30-.53) | 2.30E-10 | | 32.6 (116/240) | 1.26 (.93-1.70) | 0.14 |
| | | | | | | | | |

C. HLA with low GCNs of C4L or C4S

| | | C4L=0+1+2<br>% (no., Y/N) | OR | P | | C4S=0<br>% (no., Y/N) | OR | P |
|---|---|---|---|---|---|---|---|---|
| DR1 | | | | | | | | |
| CTL | N | 38.1 (537/871) | | | | 27.9 (392/1013) | | |
| | Y | 29.1 (122/297) | 0.67 (.53-.84) | 0.0006 | | 34.4 (144/275) | 1.35 (1.07-1.71) | 0.012 |
| IIM | N | 65.2 (401/214) | | | | 17.6 (112/526) | | |
| | Y | 51.9 (98/91) | 0.57 (.41-.80) | 0.001 | | 22.7 (45/153) | 1.38 (.93-2.04) | 0.11 |
| DR15 | | | | | | | | |
| CTL | N | 41.2 (548/781) | | | | 23.44 (311/1016) | | |
| | Y | 22.3 (111/386) | 0.41 (.32-.52) | 1.60E-14 | | 45.2 (224/272) | 2.69 (2.16-3.34) | 7.50E-19 |
| IIM | N | 63.9 (422/238) | | | | 16.4 (112/570) | | |
| | Y | 52.8 (76/68) | 0.63 (.44-.91) | 0.013 | | 29.2 (45/109) | 2.10 (1.41-3.14) | 0.0004 |
| DR3 | | | | | | | | |
| CTL | N | 18.6 (248/1087) | | | | 37.0 (493/840) | | |
| | Y | 86.5 (411/81) | 22.2 (16.9-29.3) | 3.90E-147 | | 8.76 (43/448) | 0.16 (.12-.23) | 6.80E-37 |
| IIM | N | 27.4 (96/254) | | | | 37.1 (137/232) | | |
| | Y | 88.6 (403/52) | 20.5 (14.1-29.8) | 9.50E-75 | | 4.27 (20/448) | 0.076 (.046-.12) | 8.60E-36 |
| DR4 | | | | | | | | |
| CTL | N | 42.7 (519/697) | | | | 26.1 (317/896) | | |
| | Y | 22.9 (140/471) | 0.40 (.30-.50) | 2.40E-17 | | 35.8 (219/392) | 1.58 (1.28-1.95) | 2.00E-05 |
| IIM | N | 66.4 (391/198) | | | | 15.7 (96/516) | | |
| | Y | 50.0 (107/107) | 0.51 (.37-.70) | 2.80E-05 | | 27.4 (61/162) | 2.02 (1.40-2.92) | 0.0002 |
| DR7 | | | | | | | | |
| CTL | N | 33.6 (450/888) | | | | 37.8 (505/832) | | |
| | Y | 42.8 (209/279) | 1.48 (1.20-1.83) | 0.0003 | | 6.17 (30/456) | 0.11 (.07-.16) | 2.50E-47 |
| IIM | N | 62.4 (406/245) | | | | 22.1 (151/533) | | |
| | Y | 59.5 (88/60) | 0.89 (.61-1.27) | 0.51 | | 4.08 (6/141) | 0.15 (.065-.35) | 8.10E-09 |
| DR13 | | | | | | | | |
| CTL | N | 37.6 (548/908) | | | | 30.4 (442/1012) | | |
| | Y | 29.9 (111/260) | 0.71 (.55-.90) | 0.0052 | | 25.4 (94/276) | 0.78 (.60-1.01) | 0.057 |
| IIM | N | 62.8 (428/254) | | | | 19.0 (134/572) | | |
| | Y | 57.4 (70/52) | 0.80 (.54-1.18) | 0.26 | | 17.7 (23/107) | 0.92 (.56-1.50) | 0.73 |
| DQA1*05 | | | | | | | | |
| CTL | N | 20.6 (146/563) | | | | 34.0 (240/467) | | |
| | Y | 61.9 (305/188) | 6.26 (4.84-8.09) | 1.70E-48 | | 19.5 (96/396) | 0.47 (.36-.62) | 2.80E-08 |
| IIM | N | 30.2 (75/173) | | | | 34.2 (90/173) | | |
| | Y | 77.3 (389/114) | 7.87 (5.59-11.1) | 6.30E-36 | | 11.5 (60/460) | 0.25 (.17-.36) | 1.20E-13 |
| DQA1*02 | | | | | | | | |
| CTL | N | 34.8 (303-568) | | | | 36.6 (318/552) | | |
| | Y | 44.7 (148-183) | 1.52-1.96) | 0.0016 | | 5.47 (18/311) | 0.10 (.061-.16) | 2.10E-32 |
| IIM | N | 62.7 (383/228) | | | | 22.4 (144/499) | | |
| | Y | 57.9 (81/59) | 0.82 (.5-1.19) | 0.29 | | 4.29 (6/134) | 0.16 (.067-.36) | 2.00E-08 |

| | | C4L=0+1+2 % (no., Y/N) | OR | P | | C4S=0 % (no., Y/N) | OR | P |
|---|---|---|---|---|---|---|---|---|
| DQB1*02 | | | | | | | | |
| CTL | N | 13.6 (91/576) | | | | 44.4 (296/370) | | |
| | Y | 67.3 (360/175) | 13.0 (9.79-17.3) | 3.10E-85 | | 7.50 (40/493) | 0.10 (.07-.14) | 1.70E-50 |
| IIM | N | 22.6 (57/195) | | | | 46.0 (125/147) | | |
| | Y | 81.7 (405/91) | 15.2 (10.5-22.1) | 6.10E-57 | | 4.92 (25/483) | 0.061 (.038-.097) | 5.10E-43 |
| DQB1*03 | | | | | | | | |
| CTL | N | 51.0 (278/267) | | | | 22.8 (124/419) | | |
| | Y | 26.3 (173/484) | 0.34 (.27-.44) | 1.10E-18 | | 32.3 (212/444) | 1.61 (1.25-2.09) | 0.0003 |
| IIM | N | 71.3 (296/119) | | | | 12.9 (56/377) | | |
| | Y | 49.7 (166/168) | 0.40 (.29-.54) | 1.40E-09 | | 27.0 (94/254) | 2.49 (1.73-3.60) | 7.10E-07 |
| | | | | | | | | |

*OR, odds ratio between "Y" and "N" of individual alleles in CTL (controls) or IIM (idiopathic inflammatory myopathies). N, no or absence; Y, yes or presence. no., number.

**Descriptions for Table S4.**

Specific alleles of HLA genes are known to have strong linkage disequilibrium (LD) to form ancestral haplotypes (AH) with reduced frequencies of recombination between generations. A genetic recombination between DRB1*03, the presence of short C4B1 genes and C4A-deficiency would be implicated by multi-generation genetic studies that leads to dissociation of those alleles in haplotypes.

As this is a cross-sectional, case/control study, multi-generational data of HLA gene alleles and C4 variants were not available. Thus, we sought alternative approaches to show the association of specific alleles of HLA-class II genes and complement C4 copy number variants through deciphering the association frequencies of ten common alleles of HLA class II genes (HLA-DRB1 or DR1, 2, 3, 4, 7 and 13, DQA1*05 and DQA1*02, and DQB1*02 and DQB1*03) and complement C4 copy number variants (C4T=2+3, C4A=0+1, C4B=0+1, C4L=0+1+2 and C4S=0), with detailed data presented in Supplementary Table S4.

Notably, among healthy controls, the presence of HLA-DR3 was associated with 93.2% of subjects with C4T=2+3 (OR=33.4, $p=10^{-179}$), 68.8% of C4A=0+1 (OR=39.2, $p=10^{-171}$) and 26.0% of C4B=0+1 (OR=0.74, p=0.01), and 86.5% of C4L=0+1+2 (OR=22.2, $p=10^{-147}$), plus 8.8% of C4S=0 (OR=0.16, $p=10^{-37}$).
Among IIM patients, the presence of HLA-DR3 was associated with 83.4% of subjects with C4T=2+3 (OR=20.3, $p=10^{-83}$), 70.7% of subjects with C4A=0+1 (OR=17.1, $p=10^{-70}$) and 23.5% of C4B=0+1 (OR=0.51, $p=10^{-6}$), and 88.6% of C4L=0+1+2 (OR=20.5, $p=10^{-75}$) plus 4.3% of C4S=0 (OR=0.076, $p=10^{-36}$).

Similarly, among healthy subjects, HLA-DQA1*05 alleles were associated with 61.7% of subjects with C4T=2+3 (OR=10.7, $p=10^{-71}$), 51.0% of subjects with C4A=0+1 (OR=17.4, $p=10^{-76}$), 24.1 % with C4B=0+1 (OR=0.61, p=0.0001), 61.9% with C4L=0+1+2 (OR=6.26, $p=10^{-48}$), and 19.5% with C4S=0 (OR0.47, $p=10^{-8}$).
Among IIM patients, the presence of HLA-DQA1*05 alleles were associated with 73.6% of subjects with C4T=2+3 (OR=10.3, $p=10^{-47}$), 60.6% of subjects with C4A=0+1 (OR=8.9, $p=10^{-37}$), 26.4% with C4B=0+1 (OR=0.61, p=0.0025), 77.3% with C4L=0+1+2 (OR=7.87, $p=10^{-36}$), and 11.5% with C4S=0 (OR=0.25, $p=10^{-13}$).

On HLA-DQB1*02, among healthy subjects, 56.9% had C4T=2+3 (OR=8.15, $p=10^{-57}$), 47.2% with C4A=0+1 (OR=14.4, $p=10^{-66}$) and 23.1% had C4B=0+1 (OR=0.54, $p=10^{-6}$), and 67.3% had C4L=0+1+2 (OR=13.0, $p=10^{-85}$), plus 7.5% had C4S=0 (OR=0.10, $p=10^{-50}$).
Among IIM patients, 74.1% had C4T=2+3 (OR=10.7, $p=10^{-48}$), 61.8% with C4A=0+1 (OR=10.6, $p=10^{-42}$) and 25.5% had C4B=0+1 (OR=0.56, p=0.0003), and 81.7% had C4L=0+1+2 (OR=15.2, $p=10^{-57}$), plus 4.9% had C4S=0 (OR=0.06, $p=10^{-43}$).

It is worthy to point out that HLA-DRB1*03 (DR3), C4A-deficiency (C4A=0+1), C4 gene without endogenous retrovirus HERV-K(C4) (C4L=0+1+2), C4B≥2 with the presence of C4S (C4S ≠ 0), HLA-DQA1*05 and HLA-DQB1*02 together form the ancestral haplotype (AH) 8.1. Our C4 data revealed extremely tight association remained among these alleles in the populations of healthy controls and among IIM patients, with considerable diversity or dissociations among them. Those dissociations were likely the cumulative results of historic genetic recombinations. Nevertheless, the degrees of association among specific alleles of the HLA and complement C4 gene copy number variants remain extremely striking.

**Supplementary Figures**

1. **Fig. S1**. Copy number variations of total *C4*, *C4A*, *C4B*, long genes (*C4L*) and short genes (*C4S*) in healthy controls.
2. **Fig. S2**. Comparisons of GCN variations for total C4 (panel A) and C4A (panel B) between IIM patients (red) and matched controls (blue). Arrows in panel A indicate the directions of changes.
3. **Fig**. **S3.** Logistic regression models for myositis subgroups and myositis related autoantibodies – DM, JDM, PM, IBM, MSA, MSA-Jo1, MAA, MAA-PM/Scl and MAA-Ro.

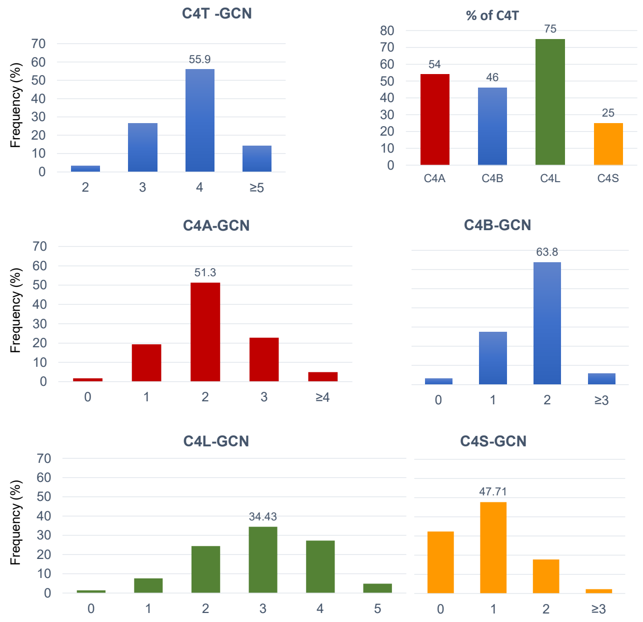


**Figure S1**. Gene copy number (GCN) variations of human complement *C4* with inherent diversities in GCNs for *C4A* and *C4B*, and for long (*C4L*) and short (*C4S*) genes among healthy subjects of European descent from the US and European countries were studied experimentally to decipher the variations and frequencies of gene copy number groups by Southern blot analyses and/or by TaqMan-based real-time PCR.

**Copy number variations of total *C4*, *C4A*, *C4B*, long genes and short genes in healthy controls.**

Gene copy numbers (GCN) for total C4 (*C4T*), *C4A*, *C4B*, long genes (*C4L*) and short genes (*C4S*) were determined in 3526 healthy control subjects (Fig. S1). There was a continuous variation in the copy number of *C4T* genes from 2 to 8 among control subjects studied. The mean GCN and standard deviation (± SD) was 3.833± 0.762. Categorically, the most common GCN group of *C4T* was 4, at a frequency of 55.9%. Healthy subjects had 3 and 2 copies of *C4T* at frequencies of 26.5% and 3.29%, respectively. Combined, 29.8% of healthy subjects had *low* copy numbers (*C4T*=2+3).  Control subjects with 5, 6, 7 and 8 copies of *C4T* occurred at frequencies of 12.4%, 1.74%, 0.14%, and 0.03%, respectively. Together, *high* copy numbers (*C4T*=5 to 8) constituted 14.3% of the healthy subjects. The low (*C4T*=2+3), medium (*C4T*=4), and high (*C4T*=5 to 8) total *C4* GCN groups had a distribution of 0.30, 0.56, and 0.14, respectively.  There was an inherent bias towards low *C4T* copy number variants and a concomitant decrease in the frequency of high copy number variants within the white population.

<u>*C4A* and *C4B*</u>.  Among our white healthy subjects, the copy number of *C4A* varied from 0 to 6. Just over half (51.3%) of the population had two copies of *C4A* genes in a diploid genome (*C4A*=2). The distributions of low and high copy number groups were relatively balanced: 1.7% for *C4A*=0 and 19.3% for *C4A*=1; 22.7% for *C4A*=3, 4.57% for *C4A*=4, 0.3% and 0.029% for *C4A*=5 and *C4A*=6. In brief, 21.0% of healthy subjects had *C4A*=0+1, 51.3% had *C4A*=2, and 27.7% had *C4A*≥3 (Fig. 2).

The copy numbers of *C4B* varied from 0 to 5. Close to two-thirds (63.8%) of the healthy subjects had 2 copies *C4B* genes. An *unbalanced* distribution of the low and high copy number groups of *C4B* was observed: 3.15% had *C4B*=0 and 27.3% had *C4B*=1, 5.52% had *C4B*=3, 0.23% had *C4B*=4, and 0.029% had *C4B*=5. Low copy number groups of *C4B* constituted 30.4%, and high copy number group just 5.8% among our healthy controls.

Among total *C4* genes, approximately 54% coded for C4A and 46% coded for C4B (Fig. 2). There was very strong and positive relationship in the variation of GCN between *C4T* and *C4A* with an $R^2$ of 0.499 ($p=4.2x10^{-322}$). While highly significant, the relationship between the GCN of *C4T* and *C4B* was *negative* with $R^2=0.061$, $p=3.93x10^{-72}$. Similarly, the copy numbers of *C4A* and *C4B* were negatively correlated, with an $R^2$ of 0.222, $p=3.5x10^{-281}$.

Long genes and short genes. The copy number of *C4L* varied between 0 and 8, with three copies being the most prevalent with a frequency of 34.4% among our healthy subjects. The frequencies for the low and high copy number groups of *C4L* were quite evenly distributed, with a total of 33.4% for *C4L*=0, 1, or 2 and 32.2% for *C4L*=4, 5, 6, 7, and 8.

The copy number of *C4S* varied from 0 to 4 but skewed heavily towards the low end among our healthy subjects: 32.3% had zero copies and 47.7% had a single copy of *C4S*. Those with 2, 3, and 4 copies of short genes constituted 17.7%, 2.01%, and 0.20% of all controls, respectively.

Overall, three quarters (75.4%) of *C4T* were *C4L* and 24.6% *C4S*. There were direct, linear, and very strong relationships between the copy numbers of long genes with *C4T* or *C4A*, whose $R^2$ were 0.485 and 0.502, respectively ($p=4.2x10^{-322}$). The correlation between copy numbers of long genes and *C4B* was loose ($R^2 = 0.0115$) with a tendency of being inversely correlated ($p=2.3x10^{-10}$).

Unlike *C4L*, the copy number of *C4S* did not correlate with *C4T* among our white control subjects (p=0.42). The *C4S* GCN was inversely correlated with *C4A* GCN ($R^2=0.0964$, $p=1.8x10^{-78}$), and positively correlated with *C4B* GCN ($R^2=0.18$, $p=1.5x10^{-152}$).

In brief, our data on the relatively large population of healthy control subjects strengthens our previous observations[24 30] on the continuous gene copy number variation of total *C4*, *C4A,* and *C4B*, as well as the size dichotomy between long and short *C4* genes. Among our control subjects, the mean gene copy numbers were 3.83 for *C4T*, 2.10 for *C4A* and 1.73 for *C4B*. The distributions of *C4T* and *C4B* were skewed towards lower GCN, but for *C4A* towards slightly higher copy number. Three quarters of total *C4* were long genes and the remainder were short. There were strong and positive correlations between *C4T* GCN and both *C4A* and *C4L* with $R^2$ values between 0.48 and 0.52. In contrast, the copy numbers of *C4B* and *C4S* were both inversely correlated with *C4A* and their relationships with *C4T* were unremarkable. While plasma C4 protein levels increased with *C4* GCN, the net C4 protein yield per copy of *C4* gene decreased with increasing GCNs of *C4T* or *C4L*. Such phenomenon may be due to less efficient transcription of the longer genes, or due to the promoter activity of the 3’ LTR from the endogenous retrovirus HERV-K(C4) inserted into *C4L*, which might generate antisense transcripts to modulate C4 biosynthesis as proposed previously.[26 27]

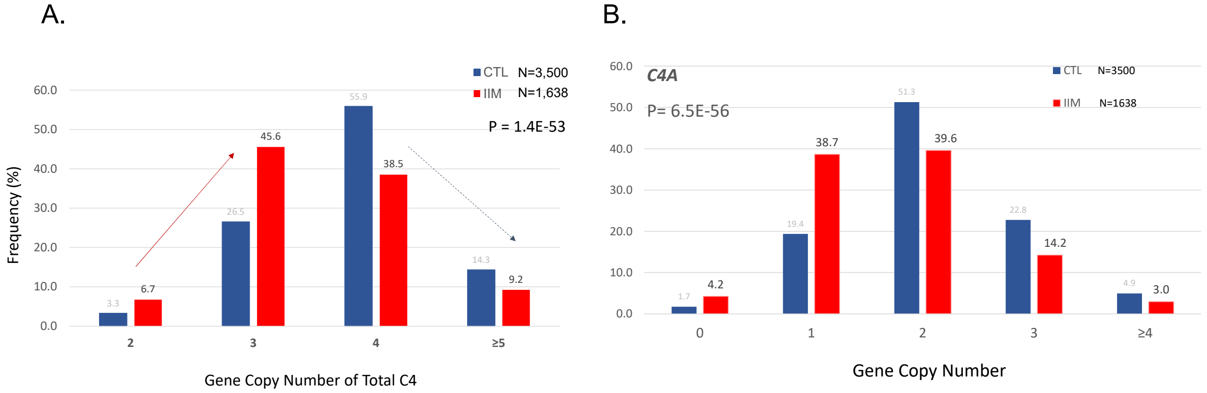


**Figure S2**. Comparisons of GCN variations for total C4 (*C4T*, panel A) and *C4A* (panel B) between IIM patients (red) and matched controls (blue). Arrows in panel A indicate the directions of changes. It is noted that more than half (52.3%) of IIM had GCN of *C4T*= 2 or 3 (panel A); *C4A* deficiency (C4A=0 or 1) constituted 43% of the IIM patients (panel B).

**Supplementary Materials: Figure S3**

**LOGISTIC REGRESSION ANALYSES OF HLA CLASS II GENES AND COMPLEMENT C4 GENETIC FACTORS FOR IDIOPATHIC INFLAMMATORY MYOPATHIES (IIM).**

Logistic regression analyses were performed for myositis subgroups with alleles of HLA class II genes DRB1, DQA1, DQB1 and complement C4 gene copy numbers for adult-onset dermatomyositis (DM), juvenile onset dermatomyositis (JDM), polymyositis (PM), inclusion-body myositis (IBM) using case-control data. Intragroup analyses of IIM patients were performed patients with and without myositis-specific autoantibodies (MSA), MSA-Jo1 autoantibodies, myositis-associated autoantibodies (MAA), MAA-PM/Scl autoantibodies, and MAA-Ro autoantibodies.

While gene copy number variants were elucidated for almost all of the study population (>3500 controls and >1500 IIM patients), four-digits data for *HLA-DQA1*, *DQB1* and *DRB1* data were available to us for 1204 British healthy controls and 810 IIM patients (750 from UK, 60 from NIH). Moreover, two-digits DRB1 data were generated from an additional 625 healthy controls in the US.

**METHODS.**

For each subgroup, significant risk factors were identified by fit “Y-by-X” analyses using ANOVA for continuous data, $\chi^2$ analyses for categorical data with the JMP16 software.

For each *HLA-DQB1*, *DQA1*, and *DQB1* genetic variant, the absence was coded 0, heterozygous and homozygous presence were coded 1 and 2, respectively. A separate column was created with "N" for the absence, and "Y" for the presence of a variant or for those coded with "1" and "2".

For gene copy number variants of complement C4, continuous GCNs of total C4 (*C4T*), *C4A*, *C4B*, long genes (*C4L*) and short genes (*C4S*) were analyzed as numeric parameters using ANOVA, and by bivariate analyses categorizing GCNs into Y/N for *C4T*=2+3, *C4A*=0+1, *C4B*=0+1, *C4L*=0+1+2, and *C4S*=0. The frequencies *C4* variants were calculated as GCNs of *C4A* / GCNs of *C4T* (*C4A/C4T*), *C4B/C4T*, *C4L/C4T* and *C4S/C4T* and compared as continuous/numeric data.

<u>Inclusion and exclusion criteria for logistic regression models</u>: In each subgroup, parameters with p-value <0.05 were eligible to enter into initial regression models. For each variant, numeric parameters (0,1,2) for *HLA*, GCNs or frequencies for *C4*, or dichotomous parameters (Y/N) were selected (or entered into the initial regression models) for the ones with smaller p-values. For the HLA variants, those with two-digits were analyzed both numerically (0,1,2) and categorically (Y/N). In some cases, four-digit variants among each two-digit group yielded *opposite* results with p-values <0.05 and they both entered into the initial regression analyses.

After the initial regression analyses for each subgroup, parameters yielding the largest p-values or with p>0.05 were removed (or excluded) one at a time, until every parameters remained in the model had a p-value <0.05.

**RESULTS.**

<u>Case-Control studies</u>

**DM:** $\chi^2$=67.7, $R^2$= 0.055, degree of freedom (df)=8, N=1424, AUC=0.663; p=$1.4\times10^{-11}$

Risk factors (increased frequencies in disease): short *C4* genes among total *C4* (*C4S/C4T*, p=0.010), gene copy numbers (GCN) for *C4B* (p=0.016), and GCN for *C4S* (p=0.021), and *DQB1*02* (p=0.011).

Protective factors (with reduced frequencies in disease): long *C4* genes among total *C4* (*C4L/C4T*, p=0.0076), GCNs of *C4L* (p=0.012), and *C4A* (p=0.021), and *DRB1*13* (p=0.0499).

**JDM:** $\chi^2$=48.8, $R^2$= 0.101, df=5, N=1262, AUC=0.739; p=$2.5\times10^{-9}$

Risk factors (with increased frequencies): *DRB1*01* (*DR1*) (p=0.0027), *DQA1*04* (p=0.0092), *C4L*=0+1+2 (p=0.026)

Protective factors (with reduced frequencies): *DRB1*15* (i.e., *DR15* or *DR2*, p=0.0002), *DQB1*0202* (p=0.0021)

**PM:** $\chi^2$=200.2, $R^2$= 0.128, df=12, N=1519; AUC=0.738; p=3.0x10$^{-36}$

Risk factors (increased frequencies): *DQB1*0201* (1.4x10$^{-7}$), *DRB1*03* (*DR3*) (p=1.3x10$^{-8}$), *C4S* among total *C4* (*C4S/C4T*: 0.0064), increased copy numbers of *C4S* (p=0.015)

Protective factors (reduced frequencies): *DQA1*01* (p=5.4x10$^{-14}$), *DQB1*0202* (p=1.7x10$^{-11}$), *DQB1*0303* (p=0.0002), *DQB1*03* (p=0.00002), *DRB1*04* (*DR4*, p=0.011), long *C4* genes among total *C4* (*C4L/C4T*: p=0.011), copy numbers of *C4T* (p=0.017) and copy numbers of *C4L* (p=0.019)

**IBM**: $\chi^2$=218.6, $R^2$= 0.285, df=10, N=1315; AUC=0.870; p=2.1x10$^{-41}$

Risk factors (increased frequencies): *DR3* (p=7.4x10$^{-10}$), *DQA1*0101* (p=0.0003), *DQB1*0201* (p=5.2x10$^{-6}$); *DQB1*0501* (p=0.0019); *C4S/C4T* (p=0.0071)

Protective factors (reduced frequencies): *DR7* (P=1.7x10$^{-12}$), *DR4* (p=0.0002), *DQA1*0102* (p=2.9x10$^{-9}$); *DQB1*03* (p=0.0013), *C4L/C4T* (p=0.0011)

<u>Intragroup analyses among IIM patients</u>

**MSA:** $\chi^2$=37.2, $R^2$= 0.0375, df=5, N=738, AUC=0.615; p=5.6x10$^{-7}$

Risk factors (increased frequencies): GCN of *C4B* (p=0.002), *DQB1*0303* (P=0.018)

Protective factors (reduced frequencies): *DQB1*0201* (p=0.0051), *DQA1*0103* (P=0.0052), *DR1* (P=0.024)

**MSA-Jo-1:** $\chi^2$=66.8, $R^2$ = 0.112, df=7, N=640, AUC=0.734; p=6.5x10$^{-12}$

Risk factors (increased frequencies): *DR3* (P=0.0023), *C4T*=2+3 (P=0.0027), *C4A*=0+1 (P=0.004), *C4L*=0+1+2 (P=0.0056), *DQB1*0201* (P=0.043)

Protective factors (reduced frequencies or levels): C4 protein levels (P=0.008), *DQB1*0501* (P=0.021).

**MAA:** $\chi^2$=69.9, $R^2$= 0.0997, df=8, N=639, AUC=0.715; p=5.2x10$^{-12}$

Risk factors (increased frequencies): *DQA1*05* (p=0.00038), *DR2* (P=0.0006), *DQB1*0604* (P=0.017), *C4B* among *C4T* (*C4B/C4T*, p=0.022), and *C4S* among *C4T* (*C4S/C4T*, p=0.043)

Protective factors (reduced frequencies or levels): C4 protein levels (p=1.2x10$^{-7}$), GCN of *C4A* (p=0.0096), and GCN of *C4L* (p=0.043)

**MAA-PM/Scl:** $\chi^2$=65.4, $R^2$= 0.175, df=8, N=688, AUC=0.806; p=4.1x$10^{-11}$

Risk factors (increased frequencies): *DQA1*05* (P=1.2X$10^{-6}$), *DQB1*0601* (P=0.024), *DQB1*0604* (P=0.042)

Protective factors (reduced frequencies or levels): C4 protein levels (p=0.00011), GCN of *C4A* (P=0.0054), *DQA1*01* (P=0.014), *DQB1*0603* (P=0.020), *C4L/C4T* (P=0.021)

**MAA-Ro**: $\chi^2$=46.5, $R^2$= 0.119, df=7, N=672, AUC=0.762; p=7.0x$10^{-8}$

Risk factors (increased frequencies or levels): GCN of *C4S* (p=0.0041), *C4S/C4T* (p=0.0065)

Protective factors (reduced frequencies or levels): *DQA1*03* (P=0.00001), *DQA1*0101* (P=0.00043), *DQA1*02* (p=0.0013), C4 protein per C4 gene (p=0.017), GCN of total *C4* (*C4T*, p=0.031).

**SUMMARY/CONCLUSIONS**:

Genetic variants of *HLA-DRB1*, *DQA1* and *DQB1*, and complement gene copy number variations for *C4T* (total *C4*), *C4A*, *C4B*, *C4L* (long genes), *C4S* (short genes) and their relative compositions *C4A/C4T*, *C4B/C4T*, *C4L/C4T* and *C4S/C4T* existed as independent risk factors or protective factors in different subgroups of IIM (DM, JDM, PM and IBM).

Among myositis patients, complement C4 protein in addition to the genetic variants described above also played a role to modulate by immune-complex mediated consumption - some of the myositis-related autoantibodies could form immune complex with host antigens to activate complement.

Some variants for HLA were genetic risk factors for IIM in case/control studies, but had complex roles as they would be protective factors in intragroup analyses for presence and absence of myositis autoantibodies (e.g., *DR2* was a protective factor for genetic risk, but a risk factor associated with MAA. Similarly, *DQB1*0201* was a strong genetic risk factor for PM, but a moderate protective factor for MSA.

# Nominal Logistic Fit for Diagnosis-2-DM

## Effect Summary

| Source | LogWorth | PValue |
|---|---|---|
| C4L/C4T | 2.121 | 0.00757 |
| C4S/C4T | 1.983 | 0.01040 |
| DQB1-02, Y/N.x | 1.944 | 0.01137 |
| C4L.x | 1.911 | 0.01228 |
| C4B.x | 1.784 | 0.01644 |
| C4S.x | 1.675 | 0.02116 |
| C4A.x | 1.673 | 0.02124 |
| DR13, Y/N.x | 1.302 | 0.04987 |

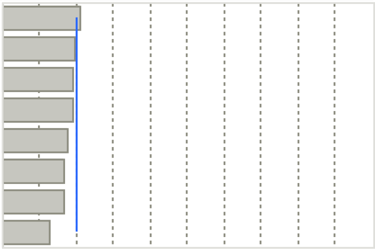

Converged in Gradient, 6 iterations

## Whole Model Test

| Model | -LogLikelihood | DF | ChiSquare | Prob>ChiSq |
|---|---|---|---|---|
| Difference | 33.84831 | 8 | 67.69663 | <.0001* |
| Full | 587.51108 | | | 1.41e-11 |
| Reduced | 621.35940 | | | |

| | |
|---|---|
| RSquare (U) | 0.0545 |
| AICc | 1193.15 |
| BIC | 1240.37 |
| Observations (or Sum Wgts) | 1424 |

## Lack Of Fit

| Source | DF | -LogLikelihood | ChiSquare |
|---|---|---|---|
| Lack Of Fit | 113 | 63.49359 | 126.9872 |
| Saturated | 121 | 524.01749 | **Prob>ChiSq** |
| Fitted | 8 | 587.51108 | 0.1740 |

## Parameter Estimates

| Term | Estimate | Std Error | ChiSquare | Prob>ChiSq | Lower 95% | Upper 95% |
|---|---|---|---|---|---|---|
| Intercept | -24.449074 | 16.122925 | 2.30 | 0.1294 | -56.049426 | 7.15127788 |
| C4A.x | 6.63750853 | 4.364652 | 2.31 | 0.1283 | -1.9170523 | 15.1920693 |
| C4B.x | 6.81986167 | 4.3656272 | 2.44 | 0.1182 | -1.7366103 | 15.3763337 |
| C4L.x | -7.0629099 | 4.3784731 | 2.60 | 0.1067 | -15.64456 | 1.51873972 |
| C4S.x | -6.6512769 | 4.3583967 | 2.33 | 0.1270 | -15.193578 | 1.89102369 |
| C4S/C4T | 22.9676812 | 16.111745 | 2.03 | 0.1540 | -8.6107593 | 54.5461217 |
| C4L/C4T | 23.5197082 | 16.141264 | 2.12 | 0.1451 | -8.1165871 | 55.1560035 |
| DR13, Y/N.x[N] | 0.22264328 | 0.1177165 | 3.58 | 0.0586 | -0.0080768 | 0.45336341 |
| DQB1-02, Y/N.x[N] | -0.2431118 | 0.096561 | 6.34 | 0.0118* | -0.4323678 | -0.0538558 |

Confidence limits are Wald-based.
For log odds of DM/CTL

## Effect Wald Tests

| Source | Nparm | DF | Wald ChiSquare | Prob>ChiSq |
|---|---|---|---|---|
| C4A.x | 1 | 1 | 2.31265543 | 0.1283 |
| C4B.x | 1 | 1 | 2.44038218 | 0.1182 |
| C4L.x | 1 | 1 | 2.60208786 | 0.1067 |
| C4S.x | 1 | 1 | 2.32893057 | 0.1270 |
| C4S/C4T | 1 | 1 | 2.03211896 | 0.1540 |
| C4L/C4T | 1 | 1 | 2.1231897 | 0.1451 |

# Nominal Logistic Fit for Diagnosis-2-DM

## Effect Wald Tests

| Source | Nparm | DF | Wald ChiSquare | Prob>ChiSq |
|---|---|---|---|---|
| DR13, Y/N.x | 1 | 1 | 3.57720984 | 0.0586 |
| DQB1-02, Y/N.x | 1 | 1 | 6.33882689 | 0.0118* |

## Effect Likelihood Ratio Tests

| Source | Nparm | DF | L-R ChiSquare | Prob>ChiSq |
|---|---|---|---|---|
| C4A.x | 1 | 1 | 5.30700853 | 0.0212* |
| C4B.x | 1 | 1 | 5.75562541 | 0.0164* |
| C4L.x | 1 | 1 | 6.27069575 | 0.0123* |
| C4S.x | 1 | 1 | 5.31375739 | 0.0212* |
| C4S/C4T | 1 | 1 | 6.56491717 | 0.0104* |
| C4L/C4T | 1 | 1 | 7.13351963 | 0.0076* |
| DR13, Y/N.x | 1 | 1 | 3.84576043 | 0.0499* |
| DQB1-02, Y/N.x | 1 | 1 | 6.40682217 | 0.0114* |

## Odds Ratios

For Diagnosis-2-DM odds of DM versus CTL

### Unit Odds Ratios

Per unit change in regressor

| Term | Odds Ratio | Lower 95% | Upper 95% | Reciprocal |
|---|---|---|---|---|
| C4A.x | 763.1912 | 0.14704 | 3961247 | 0.0013103 |
| C4B.x | 915.8583 | 0.176116 | 4762740 | 0.0010919 |
| C4L.x | 0.000856 | 1.606e-7 | 4.566467 | 1167.8385 |
| C4S.x | 0.001292 | 2.521e-7 | 6.626148 | 773.77176 |
| C4S/C4T | 9.435e+9 | 0.000182 | 4.89e+23 | 1.06e-10 |
| C4L/C4T | 1.64e+10 | 0.000299 | 8.99e+23 | 6.103e-11 |

### Range Odds Ratios

Per change in regressor over entire range

| Term | Odds Ratio | Lower 95% | Upper 95% | Reciprocal |
|---|---|---|---|---|
| C4A.x | 2.59e+14 | 6.873e-5 | 9.75e+32 | 3.862e-15 |
| C4B.x | 7.04e+11 | 0.000962 | 5.15e+26 | 1.421e-12 |
| C4L.x | 3.94e-19 | 1.71e-41 | 9067.377 | 2.537e+18 |
| C4S.x | 2.79e-12 | 4.04e-27 | 1927.723 | 3.585e+11 |
| C4S/C4T | 9.435e+9 | 0.000182 | 4.89e+23 | 1.06e-10 |
| C4L/C4T | 1.64e+10 | 0.000299 | 8.99e+23 | 6.103e-11 |

### Odds Ratios for DR13, Y/N.x

| Level1 | /Level2 ^ | Odds Ratio | Prob>Chisq | Lower 95% | Upper 95% |
|---|---|---|---|---|---|
| Y | N | 0.6406407 | 0.0586 | 0.4038439 | 1.0162849 |
| N | Y | 1.5609374 | 0.0586 | 0.9839761 | 2.4762042 |

### Odds Ratios for DQB1-02, Y/N.x

| Level1 | /Level2 ^ | Odds Ratio | Prob>Chisq | Lower 95% | Upper 95% |
|---|---|---|---|---|---|
| Y | N | 1.6261636 | 0.0118* | 1.1137265 | 2.3743783 |
| N | Y | 0.6149443 | 0.0118* | 0.4211629 | 0.8978865 |

Normal approximations used for ratio confidence limits effects:
DR13, Y/N.x DQB1-02, Y/N.x
Tests and confidence intervals on odds ratios are Wald based.

## Nominal Logistic Fit for Diagnosis-2-DM

### Receiver Operating Characteristic

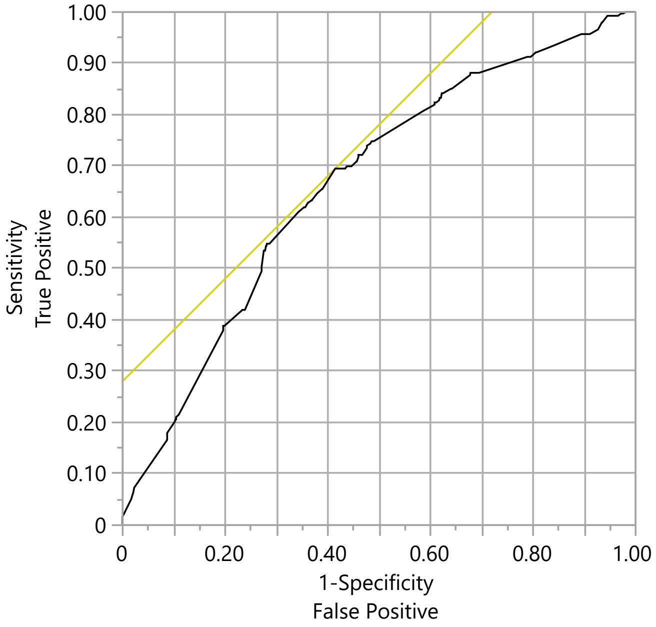


Using Diagnosis-2-DM='DM' to be the positive level

| AUC |
|---|
| 0.66267 |

BMJ Publishing Group Limited (BMJ) disclaims all liability and responsibility arising from any reliance placed on this supplemental material which has been supplied by the author(s)




# Nominal Logistic Fit for DIAGNOSIS-1-JDM

## Effect Summary

| Source | LogWorth | PValue |
|---|---|---|
| DR15, 012.x | 4.759 | 0.00002 |
| DQB1-0202.x | 3.672 | 0.00021 |
| DR1, Y/N.x | 2.570 | 0.00269 |
| DQA1-04, 012.x | 2.036 | 0.00920 |
| C4L=0+1+2.x | 1.591 | 0.02563 |

Converged in Gradient, 7 iterations

## Whole Model Test

| Model | -LogLikelihood | DF | ChiSquare | Prob>ChiSq |
|---|---|---|---|---|
| Difference | 24.39216 | 5 | 48.78432 | <.0001* |
| Full | 216.92488 | | | 2.46e-9 |
| Reduced | 241.31704 | | | |

| | |
|---|---|
| RSquare (U) | 0.1011 |
| AICc | 445.917 |
| BIC | 476.692 |
| Observations (or Sum Wgts) | 1262 |

## Lack Of Fit

| Source | DF | -LogLikelihood | ChiSquare |
|---|---|---|---|
| Lack Of Fit | 21 | 4.99972 | 9.999444 |
| Saturated | 26 | 211.92516 | **Prob>ChiSq** |
| Fitted | 5 | 216.92488 | 0.9789 |

## Parameter Estimates

| Term | Estimate | Std Error | ChiSquare | Prob>ChiSq | Lower 95% | Upper 95% |
|---|---|---|---|---|---|---|
| Intercept | -2.9506761 | 0.2170528 | 184.80 | <.0001* | -3.4194897 | -2.5587844 |
| DR15, 012.x | -1.7145435 | 0.5171033 | 10.99 | 0.0009* | -2.9082174 | -0.8274418 |
| DR1, Y/N.x[N] | 0.55316572 | 0.2072867 | 7.12 | 0.0076* | 0.17874966 | 1.00341791 |
| DQA1-04, 012.x | 1.22045727 | 0.4231453 | 8.32 | 0.0039* | 0.32825661 | 2.00711804 |
| DQB1-0202.x | -1.4236468 | 0.4693187 | 9.20 | 0.0024* | -2.4816982 | -0.6037689 |
| C4L=0+1+2.x[N] | -0.3062204 | 0.1377471 | 4.94 | 0.0262* | -0.5796994 | -0.0373308 |

Confidence limits are likelihood-based.
For log odds of JDM/CTL

## Effect Wald Tests

| Source | Nparm | DF | Wald ChiSquare | Prob>ChiSq |
|---|---|---|---|---|
| DR15, 012.x | 1 | 1 | 10.9936606 | 0.0009* |
| DR1, Y/N.x | 1 | 1 | 7.12143812 | 0.0076* |
| DQA1-04, 012.x | 1 | 1 | 8.31890508 | 0.0039* |
| DQB1-0202.x | 1 | 1 | 9.20171531 | 0.0024* |
| C4L=0+1+2.x | 1 | 1 | 4.94200617 | 0.0262* |

# Nominal Logistic Fit for DIAGNOSIS-1-JDM

## Effect Likelihood Ratio Tests

| Source | Nparm | DF | L-R ChiSquare | Prob>ChiSq |
|---|---|---|---|---|
| DR15, 012.x | 1 | 1 | 18.4540363 | <.0001* |
| DR1, Y/N.x | 1 | 1 | 9.00637931 | 0.0027* |
| DQA1-04, 012.x | 1 | 1 | 6.78385276 | 0.0092* |
| DQB1-0202.x | 1 | 1 | 13.7164282 | 0.0002* |
| C4L=0+1+2.x | 1 | 1 | 4.98082084 | 0.0256* |

## Odds Ratios

For DIAGNOSIS-1-JDM odds of JDM versus CTL

### Unit Odds Ratios

Per unit change in regressor

| Term | Odds Ratio | Lower 95% | Upper 95% | Reciprocal |
|---|---|---|---|---|
| DR15, 012.x | 0.180046 | 0.054573 | 0.437166 | 5.5541392 |
| DQA1-04, 012.x | 3.388737 | 1.388545 | 7.441839 | 0.2950952 |
| DQB1-0202.x | 0.240834 | 0.083601 | 0.546747 | 4.1522353 |

### Range Odds Ratios

Per change in regressor over entire range

| Term | Odds Ratio | Lower 95% | Upper 95% | Reciprocal |
|---|---|---|---|---|
| DR15, 012.x | 0.032417 | 0.002978 | 0.191114 | 30.848463 |
| DQA1-04, 012.x | 3.388737 | 1.388545 | 7.441839 | 0.2950952 |
| DQB1-0202.x | 0.058001 | 0.006989 | 0.298932 | 17.241058 |

### Odds Ratios for DR1, Y/N.x

| Level1 | /Level2 ^ | Odds Ratio | Prob>Chisq | Lower 95% | Upper 95% |
|---|---|---|---|---|---|
| Y | N | 0.3307702 | 0.0076* | 0.1467712 | 0.7454386 |
| N | Y | 3.0232471 | 0.0076* | 1.3414922 | 6.8133254 |

### Odds Ratios for C4L=0+1+2.x

| Level1 | /Level2 ^ | Odds Ratio | Prob>Chisq | Lower 95% | Upper 95% |
|---|---|---|---|---|---|
| Y | N | 1.8449289 | 0.0262* | 1.0751735 | 3.1657797 |
| N | Y | 0.5420263 | 0.0262* | 0.3158779 | 0.9300824 |

Normal approximations used for ratio confidence limits effects:
DR1, Y/N.x C4L=0+1+2.x
Tests and confidence intervals on odds ratios are Wald based.

## Nominal Logistic Fit for DIAGNOSIS-1-JDM

### Receiver Operating Characteristic

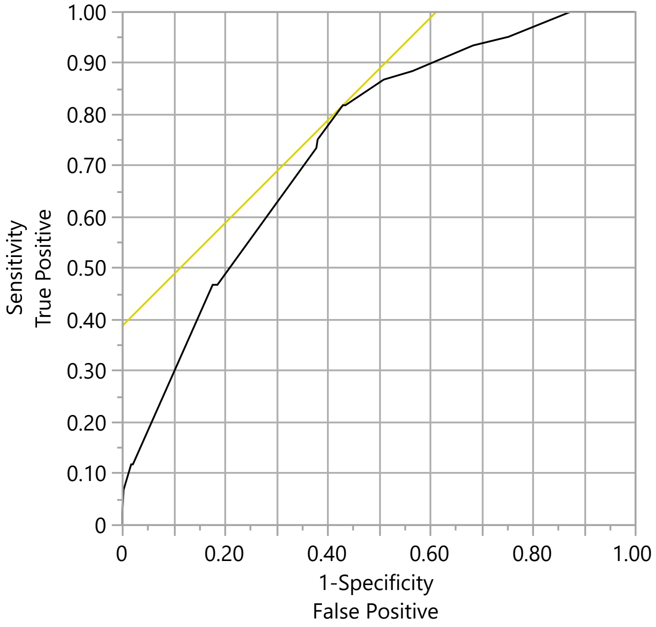


Using DIAGNOSIS-1-JDM='JDM' to be the positive level

| AUC |
|---|
| 0.73898 |

## Nominal Logistic Fit for DIAGNOSIS-3-PM

### Effect Summary

| Source | LogWorth | PValue |
|---|---|---|
| DQA1-01, 012.x | 13.311 | 0.00000 |
| DQB1-0202.x | 11.749 | 0.00000 |
| DR3, Y/N.x | 8.588 | 0.00000 |
| DQB1-0201.x | 7.523 | 0.00000 |
| DQB1-03, Y/N.x | 4.631 | 0.00002 |
| DQB1-0303.x | 3.388 | 0.00041 |
| C4S/C4T | 2.193 | 0.00641 |
| DR4, Y/N.x | 1.953 | 0.01115 |
| C4L/C4T | 1.952 | 0.01117 |
| C4S.x | 1.832 | 0.01472 |
| C4T.x | 1.761 | 0.01733 |
| C4L.x | 1.717 | 0.01920 |

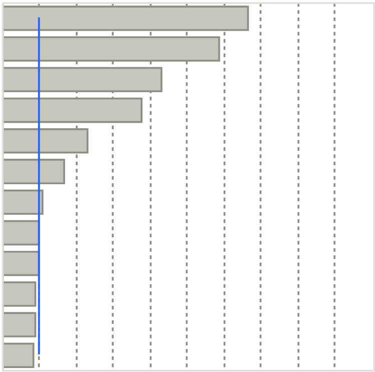

Converged in Gradient, 5 iterations

### Whole Model Test

| Model | -LogLikelihood | DF | ChiSquare | Prob>ChiSq |
|---|---|---|---|---|
| Difference | 100.09828 | 12 | 200.1966 | <.0001* |
| Full | 681.93806 | | | |
| Reduced | 782.03633 | | | |

2.97e-36

| | |
|---|---|
| RSquare (U) | 0.1280 |
| AICc | 1390.12 |
| BIC | 1459.11 |
| Observations (or Sum Wgts) | 1519 |

### Lack Of Fit

| Source | DF | -LogLikelihood | ChiSquare |
|---|---|---|---|
| Lack Of Fit | 197 | 109.06721 | 218.1344 |
| Saturated | 209 | 572.87085 | **Prob>ChiSq** |
| Fitted | 12 | 681.93806 | 0.1441 |

### Parameter Estimates

| Term | Estimate | Std Error | ChiSquare | Prob>ChiSq |
|---|---|---|---|---|
| Intercept | -39.576123 | 19.957461 | 3.93 | 0.0474* |
| C4T.x | 11.3043464 | 5.401229 | 4.38 | 0.0364* |
| C4L.x | -11.161115 | 5.4125976 | 4.25 | 0.0392* |
| C4S.x | -11.542437 | 5.3936781 | 4.58 | 0.0324* |
| C4S/C4T | 42.0468455 | 19.962438 | 4.44 | 0.0352* |
| C4L/C4T | 39.894952 | 19.995756 | 3.98 | 0.0460* |
| DR3, Y/N.x[N] | -1.1836399 | 0.2081769 | 32.33 | <.0001* |
| DR4, Y/N.x[N] | 0.27691334 | 0.1083691 | 6.53 | 0.0106* |
| DQA1-01, 012.x | -1.4436538 | 0.191943 | 56.57 | <.0001* |
| DQB1-0201.x | -2.2623721 | 0.4291822 | 27.79 | <.0001* |
| DQB1-0202.x | -1.784089 | 0.2651107 | 45.29 | <.0001* |
| DQB1-03, Y/N.x[N] | 0.55829455 | 0.1339402 | 17.37 | <.0001* |
| DQB1-0303.x | -1.0295629 | 0.3117423 | 10.91 | 0.0010* |

For log odds of PM/CTL

1.3e-8

1.5.4e-14
1.4e-7
1.7e-11
[illegible]

## Nominal Logistic Fit for DIAGNOSIS-3-PM

### Effect Likelihood Ratio Tests

[illegible]

| Source | Nparm | DF | L-R ChiSquare | Prob>ChiSq |
|---|---|---|---|---|
| C4T.x | 1 | 1 | 5.66288832 | 0.0173* |
| C4L.x | 1 | 1 | 5.48357059 | 0.0192* |
| C4S.x | 1 | 1 | 5.95022439 | 0.0147* |
| C4S/C4T | 1 | 1 | 7.43120096 | 0.0064* |
| C4L/C4T | 1 | 1 | 6.43859332 | 0.0112* |
| DR3, Y/N.x | 1 | 1 | 35.4781079 | <.0001* |
| DR4, Y/N.x | 1 | 1 | 6.44136733 | 0.0111* |
| DQA1-01, 012.x | 1 | 1 | 56.7732304 | <.0001* |
| DQB1-0201.x | 1 | 1 | 30.7097381 | <.0001* |
| DQB1-0202.x | 1 | 1 | 49.711019 | <.0001* |
| DQB1-03, Y/N.x | 1 | 1 | 17.8910252 | <.0001* |
| DQB1-0303.x | 1 | 1 | 12.4881129 | 0.0004* |

# Nominal Logistic Fit for DIAGNOSIS-4-IBM

## Effect Summary

| Source | LogWorth | PValue |
|---|---|---|
| DR7, Y/N.x | 11.783 | 0.00000 |
| DR3, Y/N.y | 9.130 | 0.00000 |
| DQA1-0102.x | 8.541 | 0.00000 |
| DQB1-0201.x | 5.283 | 0.00001 |
| DR4, Y/N.x | 3.604 | 0.00025 |
| DQA1-0101.x | 3.573 | 0.00027 |
| C4L/C4T | 2.976 | 0.00106 |
| DQB1-03, Y/N.x | 2.887 | 0.00130 |
| DQB1-0501.x | 2.713 | 0.00194 |
| C4S/C4T | 2.113 | 0.00771 |

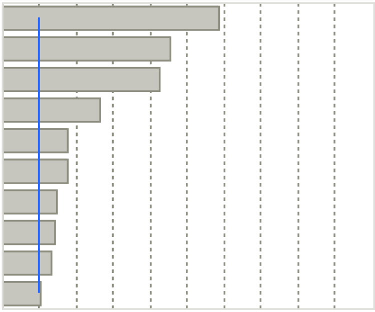

Converged in Gradient, 7 iterations

## Whole Model Test

| Model | -LogLikelihood | DF | ChiSquare | Prob>ChiSq |
|---|---|---|---|---|
| Difference | 109.31073 | 10 | 218.6215 | <.0001* |
| Full | 273.64462 | | | 2.08e-41 |
| Reduced | 382.95535 | | | |

| | |
|---|---|
| RSquare (U) | 0.2854 |
| AICc | 569.492 |
| BIC | 626.287 |
| Observations (or Sum Wgts) | 1315 |

## Lack Of Fit

| Source | DF | -LogLikelihood | ChiSquare |
|---|---|---|---|
| Lack Of Fit | 215 | 67.07677 | 134.1535 |
| Saturated | 225 | 206.56785 | **Prob>ChiSq** |
| Fitted | 10 | 273.64462 | 1.0000 |

## Parameter Estimates

| Term | Estimate | Std Error | ChiSquare | Prob>ChiSq | Lower 95% | Upper 95% |
|---|---|---|---|---|---|---|
| Intercept | 0.87099072 | 1.142016 | 0.58 | 0.4457 | -1.3673196 | 3.109301 |
| DR4, Y/N.x[N] | 0.75818734 | 0.2147325 | 12.47 | 0.0004* | 0.33731943 | 1.17905524 |
| DR7, Y/N.x[N] | 1.56556287 | 0.286289 | 29.90 | <.0001* | 1.0044468 | 2.12667893 |
| DQB1-0201.x | -2.5340616 | 0.6223254 | 16.58 | <.0001* | -3.753797 | -1.3143263 |
| DQB1-03, Y/N.x[N] | 0.57119763 | 0.182154 | 9.83 | 0.0017* | 0.21418232 | 0.92821295 |
| DQB1-0501.x | 2.13004415 | 0.825123 | 6.66 | 0.0098* | 0.51283276 | 3.74725554 |
| C4L/C4T | -3.1676395 | 1.0555334 | 9.01 | 0.0027* | -5.236447 | -1.0988319 |
| DR3, Y/N.y[N] | -1.6833114 | 0.3099714 | 29.49 | <.0001* | -2.2908441 | -1.0757787 |
| DQA1-0101.x | -2.5342215 | 0.8614666 | 8.65 | 0.0033* | -4.2226649 | -0.845778 |
| DQA1-0102.x | -1.9088016 | 0.3401315 | 31.49 | <.0001* | -2.5754471 | -1.2421561 |
| C4S/C4T | -2.6941365 | 1.0790596 | 6.23 | 0.0125* | -4.8090546 | -0.5792185 |

Confidence limits are Wald-based.
For log odds of IBM/CTL

# Nominal Logistic Fit for DIAGNOSIS-4-IBM

## Effect Wald Tests

| Source | Nparm | DF | Wald ChiSquare | Prob>ChiSq |
|---|---|---|---|---|
| DR4, Y/N.x | 1 | 1 | 12.4668752 | 0.0004* |
| DR7, Y/N.x | 1 | 1 | 29.9041733 | <.0001* |
| DQB1-0201.x | 1 | 1 | 16.5805638 | <.0001* |
| DQB1-03, Y/N.x | 1 | 1 | 9.83320928 | 0.0017* |
| DQB1-0501.x | 1 | 1 | 6.664074 | 0.0098* |
| C4L/C4T | 1 | 1 | 9.00590782 | 0.0027* |
| DR3, Y/N.y | 1 | 1 | 29.4907471 | <.0001* |
| DQA1-0101.x | 1 | 1 | 8.65390791 | 0.0033* |
| DQA1-0102.x | 1 | 1 | 31.4940009 | <.0001* |
| C4S/C4T | 1 | 1 | 6.23373451 | 0.0125* |

## Effect Likelihood Ratio Tests

| Source | Nparm | DF | L-R ChiSquare | Prob>ChiSq |
|---|---|---|---|---|
| DR4, Y/N.x | 1 | 1 | 13.419904 | 0.0002* |
| DR7, Y/N.x | 1 | 1 | 49.8632292 | <.0001* |
| DQB1-0201.x | 1 | 1 | 20.7595573 | <.0001* |
| DQB1-03, Y/N.x | 1 | 1 | 10.3475808 | 0.0013* |
| DQB1-0501.x | 1 | 1 | 9.60965547 | 0.0019* |
| C4L/C4T | 1 | 1 | 10.7257669 | 0.0011* |
| DR3, Y/N.y | 1 | 1 | 37.9103039 | <.0001* |
| DQA1-0101.x | 1 | 1 | 13.284815 | 0.0003* |
| DQA1-0102.x | 1 | 1 | 35.2663865 | <.0001* |
| C4S/C4T | 1 | 1 | 7.10017751 | 0.0077* |

1.7e-12
5.2e-6

## Odds Ratios

7.4e-10

For DIAGNOSIS-4-IBM odds of IBM versus CTL

### Unit Odds Ratios

Per unit change in regressor

2.9e-9

| Term | Odds Ratio | Lower 95% | Upper 95% | Reciprocal |
|---|---|---|---|---|
| DQB1-0201.x | 0.079336 | 0.023429 | 0.268655 | 12.604597 |
| DQB1-0501.x | 8.415238 | 1.670015 | 42.40454 | 0.118832 |
| C4L/C4T | 0.042103 | 0.005319 | 0.33326 | 23.751353 |
| DQA1-0101.x | 0.079323 | 0.01466 | 0.429223 | 12.606612 |
| DQA1-0102.x | 0.148258 | 0.07612 | 0.288761 | 6.7450007 |
| C4S/C4T | 0.067601 | 0.008156 | 0.560336 | 14.79274 |

### Range Odds Ratios

Per change in regressor over entire range

| Term | Odds Ratio | Lower 95% | Upper 95% | Reciprocal |
|---|---|---|---|---|
| DQB1-0201.x | 0.006294 | 0.000549 | 0.072176 | 158.87588 |
| DQB1-0501.x | 70.81624 | 2.788951 | 1798.145 | 0.0141211 |
| C4L/C4T | 0.042103 | 0.005319 | 0.33326 | 23.751353 |
| DQA1-0101.x | 0.006292 | 0.000215 | 0.184233 | 158.92667 |
| DQA1-0102.x | 0.02198 | 0.005794 | 0.083383 | 45.495035 |
| C4S/C4T | 0.067601 | 0.008156 | 0.560336 | 14.79274 |

## Nominal Logistic Fit for DIAGNOSIS-4-IBM

### Odds Ratios

#### Odds Ratios for DR4, Y/N.x

| Level1 | /Level2 ^ | Odds Ratio | Prob>Chisq | Lower 95% | Upper 95% |
|---|---|---|---|---|---|
| Y | N | 0.2195062 | 0.0004* | 0.0945988 | 0.5093403 |
| N | Y | 4.5556794 | 0.0004* | 1.9633238 | 10.570959 |

#### Odds Ratios for DR7, Y/N.x

| Level1 | /Level2 ^ | Odds Ratio | Prob>Chisq | Lower 95% | Upper 95% |
|---|---|---|---|---|---|
| Y | N | 0.0436686 | <.0001* | 0.0142164 | 0.134137 |
| N | Y | 22.899744 | <.0001* | 7.4550645 | 70.341213 |

#### Odds Ratios for DQB1-03, Y/N.x

| Level1 | /Level2 ^ | Odds Ratio | Prob>Chisq | Lower 95% | Upper 95% |
|---|---|---|---|---|---|
| Y | N | 0.3190539 | 0.0017* | 0.15623 | 0.6515738 |
| N | Y | 3.1342668 | 0.0017* | 1.5347456 | 6.4008187 |

#### Odds Ratios for DR3, Y/N.y

| Level1 | /Level2 ^ | Odds Ratio | Prob>Chisq | Lower 95% | Upper 95% |
|---|---|---|---|---|---|
| Y | N | 28.980489 | <.0001* | 8.5982387 | 97.679161 |
| N | Y | 0.034506 | <.0001* | 0.0102376 | 0.1163029 |

Normal approximations used for ratio confidence limits effects:
DR4, Y/N.x DR7, Y/N.x DQB1-03, Y/N.x DR3, Y/N.y
Tests and confidence intervals on odds ratios are Wald based.

### Receiver Operating Characteristic

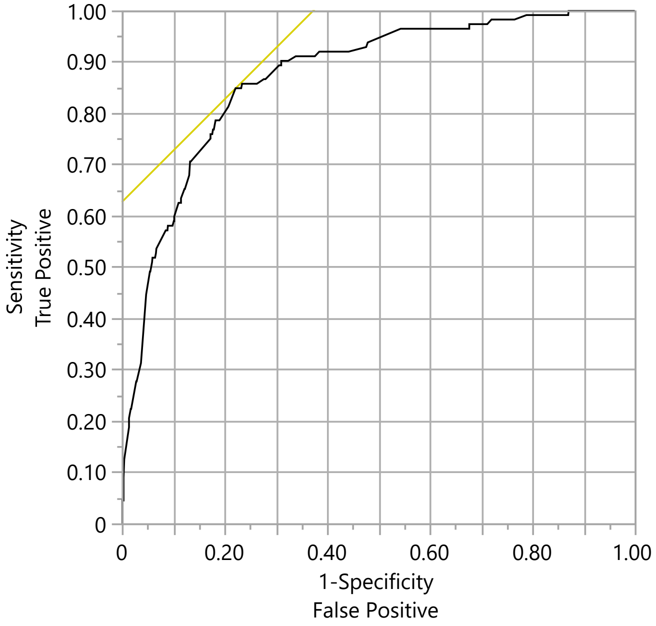


Using DIAGNOSIS-4-IBM='IBM' to be the positive level

| AUC |
|---|
| 0.87019 |

# Nominal Logistic Fit for MSA-status

## Effect Summary

| Source | LogWorth | PValue |
|---|---|---|
| C4B.x | 2.698 | 0.00200 |
| DQB1-0201.x | 2.291 | 0.00512 |
| DQA1-0103.x | 2.282 | 0.00523 |
| DQB1-0303.x | 1.747 | 0.01792 |
| DR1, Y/N.x | 1.628 | 0.02354 |

Converged in Gradient, 4 iterations

## Whole Model Test

| Model | -LogLikelihood | DF | ChiSquare | Prob>ChiSq |
|---|---|---|---|---|
| Difference | 18.57833 | 5 | 37.15665 | <.0001* |
| Full | 476.35145 | | | 5.6e-7 |
| Reduced | 494.92978 | | | |

| | |
|---|---|
| RSquare (U) | 0.0375 |
| AICc | 964.818 |
| BIC | 992.327 |
| Observations (or Sum Wgts) | 738 |

## Lack Of Fit

| Source | DF | -LogLikelihood | ChiSquare |
|---|---|---|---|
| Lack Of Fit | 36 | 24.45735 | 48.91469 |
| Saturated | 41 | 451.89411 | **Prob>ChiSq** |
| Fitted | 5 | 476.35145 | 0.0739 |

## Parameter Estimates

| Term | Estimate | Std Error | ChiSquare | Prob>ChiSq | Lower 95% | Upper 95% |
|---|---|---|---|---|---|---|
| Intercept | -0.9999985 | 0.2636746 | 14.38 | 0.0001* | -1.5167911 | -0.4832058 |
| C4B.x | 0.41356434 | 0.1358524 | 9.27 | 0.0023* | 0.14729852 | 0.67983015 |
| DR1, Y/N.x[N] | 0.20988763 | 0.0936181 | 5.03 | 0.0250* | 0.0263995 | 0.39337576 |
| DQA1-0103.x | -0.7366395 | 0.2755219 | 7.15 | 0.0075* | -1.2766524 | -0.1966265 |
| DQB1-0201.x | -0.3948919 | 0.1419779 | 7.74 | 0.0054* | -0.6731635 | -0.1166202 |
| DQB1-0303.x | 0.77169417 | 0.3328647 | 5.37 | 0.0204* | 0.11929142 | 1.42409691 |

Confidence limits are Wald-based.
For log odds of MSA-pos/MSA-neg

## Effect Wald Tests

| Source | Nparm | DF | Wald ChiSquare | Prob>ChiSq |
|---|---|---|---|---|
| C4B.x | 1 | 1 | 9.26726374 | 0.0023* |
| DR1, Y/N.x | 1 | 1 | 5.02636387 | 0.0250* |
| DQA1-0103.x | 1 | 1 | 7.14821775 | 0.0075* |
| DQB1-0201.x | 1 | 1 | 7.73596858 | 0.0054* |
| DQB1-0303.x | 1 | 1 | 5.37471031 | 0.0204* |

# Nominal Logistic Fit for MSA-status

## Effect Likelihood Ratio Tests

| Source | Nparm | DF | L-R ChiSquare | Prob>ChiSq |
|---|---|---|---|---|
| C4B.x | 1 | 1 | 9.54685192 | 0.0020* |
| DR1, Y/N.x | 1 | 1 | 5.12849816 | 0.0235* |
| DQA1-0103.x | 1 | 1 | 7.79855968 | 0.0052* |
| DQB1-0201.x | 1 | 1 | 7.8372347 | 0.0051* |
| DQB1-0303.x | 1 | 1 | 5.60400749 | 0.0179* |

## Odds Ratios

For MSA-status odds of MSA-pos versus MSA-neg

### Unit Odds Ratios

Per unit change in regressor

| Term | Odds Ratio | Lower 95% | Upper 95% | Reciprocal |
|---|---|---|---|---|
| C4B.x | 1.512198 | 1.1587 | 1.973542 | 0.661289 |
| DQA1-0103.x | 0.47872 | 0.27897 | 0.821497 | 2.0889039 |
| DQB1-0201.x | 0.673753 | 0.510092 | 0.889923 | 1.4842237 |
| DQB1-0303.x | 2.163428 | 1.126698 | 4.154105 | 0.4622293 |

### Range Odds Ratios

Per change in regressor over entire range

| Term | Odds Ratio | Lower 95% | Upper 95% | Reciprocal |
|---|---|---|---|---|
| C4B.x | 5.229195 | 1.802535 | 15.17001 | 0.191234 |
| DQA1-0103.x | 0.229173 | 0.077824 | 0.674858 | 4.3635193 |
| DQB1-0201.x | 0.453943 | 0.260194 | 0.791963 | 2.20292 |
| DQB1-0303.x | 4.680422 | 1.269449 | 17.25659 | 0.2136559 |

### Odds Ratios for DR1, Y/N.x

| Level1 | /Level2 ^ | Odds Ratio | Prob>Chisq | Lower 95% | Upper 95% |
|---|---|---|---|---|---|
| Y | N | 0.6571945 | 0.0250* | 0.4553215 | 0.9485707 |
| N | Y | 1.5216195 | 0.0250* | 1.0542177 | 2.1962503 |

Normal approximations used for ratio confidence limits effects:
DR1, Y/N.x
Tests and confidence intervals on odds ratios are Wald based.

## Nominal Logistic Fit for MSA-status

### Receiver Operating Characteristic

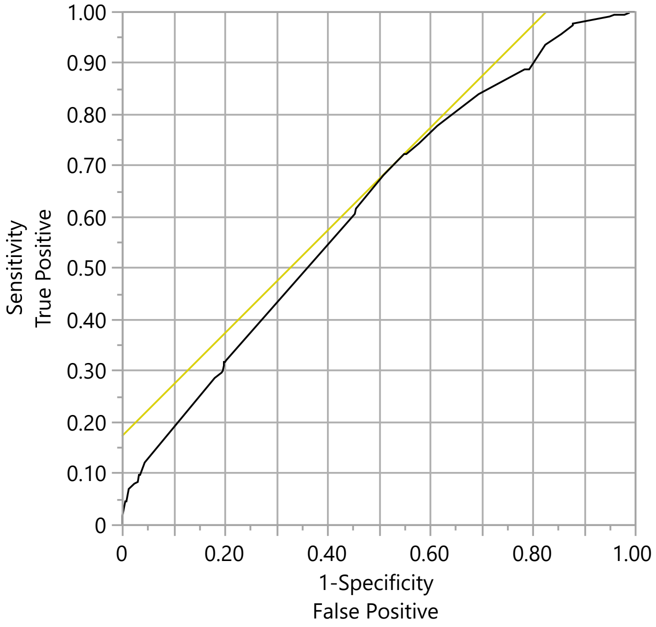


Using MSA-status='MSA-pos' to be the positive level

| AUC |
|---|
| 0.61538 |

Jo1-model-0819-2022-IIM-5183 - Fit Nominal Logistic



# Nominal Logistic Fit for MSA-Jo-1 status...19

## Effect Summary

| Source | LogWorth | PValue |
|---|---|---|
| DR3, Y/N.y | 2.637 | 0.00231 |
| C4T=2+3.x | 2.570 | 0.00269 |
| C4A=0+1.x | 2.394 | 0.00403 |
| C4L=0+1+2.x | 2.251 | 0.00562 |
| C4 Protein (mg/L) | 2.096 | 0.00801 |
| DQB1-0501.x | 1.679 | 0.02096 |
| DQB1-0201.x | 1.370 | 0.04268 |

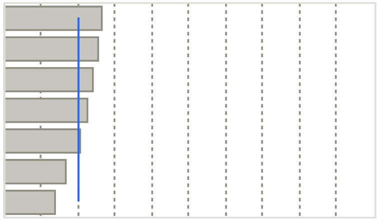

Converged in Gradient, 5 iterations

## Whole Model Test

| Model | -LogLikelihood | DF | ChiSquare | Prob>ChiSq |
|---|---|---|---|---|
| Difference | 33.40289 | 7 | 66.80579 | <.0001* |
| Full | 264.92723 | | | |
| Reduced | 298.33012 | | | |

6.5e-12

| | |
|---|---|
| RSquare (U) | 0.1120 |
| AICc | 546.083 |
| BIC | 581.546 |
| Observations (or Sum Wgts) | 640 |

## Lack Of Fit

| Source | DF | -LogLikelihood | ChiSquare |
|---|---|---|---|
| Lack Of Fit | 574 | 239.26723 | 478.5345 |
| Saturated | 581 | 25.66000 | **Prob>ChiSq** |
| Fitted | 7 | 264.92723 | 0.9985 |

## Parameter Estimates

| Term | Estimate | Std Error | ChiSquare | Prob>ChiSq | Lower 95% | Upper 95% |
|---|---|---|---|---|---|---|
| Intercept | -0.2781314 | 0.4745631 | 0.34 | 0.5578 | -1.2072233 | 0.65639437 |
| C4 Protein (mg/L) | -0.0032407 | 0.0012587 | 6.63 | 0.0100* | -0.0057665 | -0.0008276 |
| DR3, Y/N.y[N] | -0.8111884 | 0.2731475 | 8.82 | 0.0030* | -1.3595206 | -0.2853102 |
| DQB1-0201.x | -0.8459837 | 0.4333342 | 3.81 | 0.0509 | -1.7384887 | -0.0270539 |
| DQB1-0501.x | -0.6485848 | 0.2955753 | 4.82 | 0.0282* | -1.258384 | -0.0940762 |
| C4T=2+3.x[N] | 0.64736674 | 0.2194972 | 8.70 | 0.0032* | 0.22310028 | 1.08810788 |
| C4A=0+1.x[N] | -0.4784785 | 0.1750103 | 7.47 | 0.0063* | -0.8361527 | -0.1475709 |
| C4L=0+1+2.x[N] | -0.5962761 | 0.2130985 | 7.83 | 0.0051* | -1.0161477 | -0.1763987 |

Confidence limits are likelihood-based.

For log odds of Jo-1-positive/Jo1-neg

## Effect Wald Tests

| Source | Nparm | DF | Wald ChiSquare | Prob>ChiSq |
|---|---|---|---|---|
| C4 Protein (mg/L) | 1 | 1 | 6.62850167 | 0.0100* |
| DR3, Y/N.y | 1 | 1 | 8.81960474 | 0.0030* |
| DQB1-0201.x | 1 | 1 | 3.81134313 | 0.0509 |
| DQB1-0501.x | 1 | 1 | 4.81501048 | 0.0282* |
| C4T=2+3.x | 1 | 1 | 8.69846742 | 0.0032* |
| C4A=0+1.x | 1 | 1 | 7.47476685 | 0.0063* |
| C4L=0+1+2.x | 1 | 1 | 7.82950398 | 0.0051* |

## Nominal Logistic Fit for MSA-Jo-1 status...19

### Effect Likelihood Ratio Tests

| Source | Nparm | DF | L-R ChiSquare | Prob>ChiSq |
|---|---|---|---|---|
| C4 Protein (mg/L) | 1 | 1 | 7.03076864 | 0.0080* |
| DR3, Y/N.y | 1 | 1 | 9.28743856 | 0.0023* |
| DQB1-0201.x | 1 | 1 | 4.10784242 | 0.0427* |
| DQB1-0501.x | 1 | 1 | 5.32991786 | 0.0210* |
| C4T=2+3.x | 1 | 1 | 9.00648199 | 0.0027* |
| C4A=0+1.x | 1 | 1 | 8.26816256 | 0.0040* |
| C4L=0+1+2.x | 1 | 1 | 7.66978975 | 0.0056* |

### Odds Ratios

For MSA-Jo-1 status...19 odds of Jo-1-positive versus Jo1-neg

#### Unit Odds Ratios

Per unit change in regressor

| Term | Odds Ratio | Lower 95% | Upper 95% | Reciprocal |
|---|---|---|---|---|
| C4 Protein (mg/L) | 0.996765 | 0.99425 | 0.999173 | 1.003246 |
| DQB1-0201.x | 0.429135 | 0.175786 | 0.973309 | 2.330269 |
| DQB1-0501.x | 0.522785 | 0.284113 | 0.910213 | 1.9128319 |

#### Range Odds Ratios

Per change in regressor over entire range

| Term | Odds Ratio | Lower 95% | Upper 95% | Reciprocal |
|---|---|---|---|---|
| C4 Protein (mg/L) | 0.075434 | 0.010064 | 0.516855 | 13.256576 |
| DQB1-0201.x | 0.184157 | 0.030901 | 0.94733 | 5.4301538 |
| DQB1-0501.x | 0.273304 | 0.08072 | 0.828488 | 3.6589258 |

#### Odds Ratios for DR3, Y/N.y

| Level1 | /Level2 ^ | Odds Ratio | Prob>Chisq | Lower 95% | Upper 95% |
|---|---|---|---|---|---|
| Y | N | 5.0651146 | 0.0030* | 1.7361298 | 14.777343 |
| N | Y | 0.1974289 | 0.0030* | 0.0676712 | 0.5759938 |

#### Odds Ratios for C4T=2+3.x

| Level1 | /Level2 ^ | Odds Ratio | Prob>Chisq | Lower 95% | Upper 95% |
|---|---|---|---|---|---|
| Y | N | 0.2739709 | 0.0032* | 0.1158862 | 0.6477048 |
| N | Y | 3.650023 | 0.0032* | 1.5439132 | 8.6291563 |

#### Odds Ratios for C4A=0+1.x

| Level1 | /Level2 ^ | Odds Ratio | Prob>Chisq | Lower 95% | Upper 95% |
|---|---|---|---|---|---|
| Y | N | 2.6037612 | 0.0063* | 1.3111823 | 5.1705797 |
| N | Y | 0.3840598 | 0.0063* | 0.1934019 | 0.7626705 |

#### Odds Ratios for C4L=0+1+2.x

| Level1 | /Level2 ^ | Odds Ratio | Prob>Chisq | Lower 95% | Upper 95% |
|---|---|---|---|---|---|
| Y | N | 3.2954815 | 0.0051* | 1.4293527 | 7.5979835 |
| N | Y | 0.3034458 | 0.0051* | 0.1316139 | 0.6996174 |

Normal approximations used for ratio confidence limits effects:
DR3, Y/N.y C4T=2+3.x C4A=0+1.x C4L=0+1+2.x
Tests and confidence intervals on odds ratios are Wald based.

## Nominal Logistic Fit for MSA-Jo-1 status...19

### Receiver Operating Characteristic

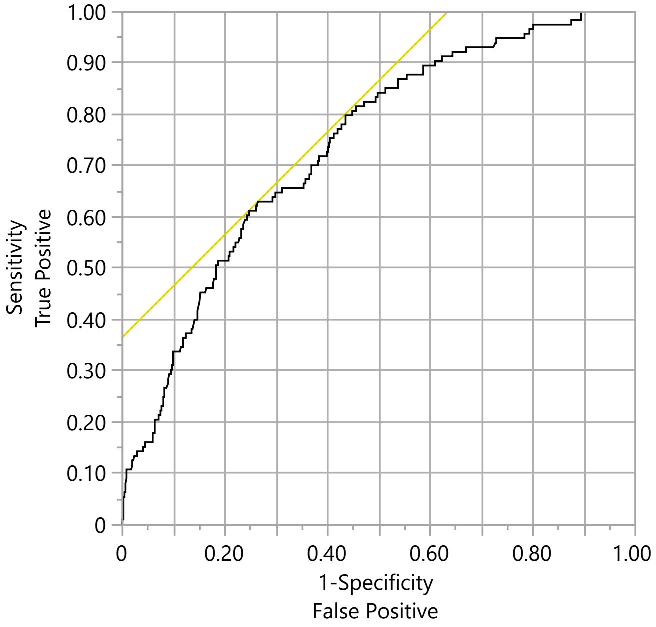


Using MSA-Jo-1 status...19='Jo-1-positive' to be the positive level

| AUC |
|---|
| 0.73445 |

# Nominal Logistic Fit for MAA-status...21

## Effect Summary

| Source | LogWorth | PValue |
|---|---|---|
| C4 Protein (mg/L) | 7.933 | 0.00000 |
| DQA1-05, Y/N.x | 3.415 | 0.00038 |
| DR15, Y/N.x | 3.225 | 0.00060 |
| C4A.x | 2.016 | 0.00964 |
| DQB1-0604.x | 1.774 | 0.01681 |
| C4B/C4T.x | 1.662 | 0.02179 |
| C4L.x | 1.371 | 0.04251 |
| C4S/C4T | 1.363 | 0.04334 |

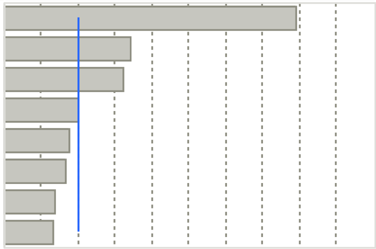

Converged in Gradient, 5 iterations

## Whole Model Test

| Model | -LogLikelihood | DF | ChiSquare | Prob>ChiSq |
|---|---|---|---|---|
| Difference | 34.93889 | 8 | 69.87777 | <.0001* |
| Full | 315.62559 | | | 5.2e-12 |
| Reduced | 350.56448 | | | |

| | |
|---|---|
| RSquare (U) | 0.0997 |
| AICc | 649.537 |
| BIC | 689.39 |
| Observations (or Sum Wgts) | 639 |

## Lack Of Fit

| Source | DF | -LogLikelihood | ChiSquare |
|---|---|---|---|
| Lack Of Fit | 612 | 312.85300 | 625.706 |
| Saturated | 620 | 2.77259 | **Prob>ChiSq** |
| Fitted | 8 | 315.62559 | 0.3417 |

## Parameter Estimates

| Term | Estimate | Std Error | ChiSquare | Prob>ChiSq | Lower 95% | Upper 95% | |
|---|---|---|---|---|---|---|---|
| Intercept | -1.3403032 | 1.1621351 | 1.33 | 0.2488 | -3.6180461 | 0.93743972 | |
| C4 Protein (mg/L) | -0.0064158 | 0.0012118 | 28.03 | <.0001* | -0.0087909 | -0.0040406 | |
| C4A.x | 1.31395732 | 0.5092112 | 6.66 | 0.0099* | 0.3159218 | 2.31199285 | |
| C4L.x | -0.6296967 | 0.3085892 | 4.16 | 0.0413* | -1.2345204 | -0.0248729 | |
| C4S/C4T | -2.168935 | 1.0583616 | 4.20 | 0.0404* | -4.2432856 | -0.0945844 | |
| C4B/C4T.x | 3.90671909 | 1.7207322 | 5.15 | 0.0232* | 0.53414595 | 7.27929223 | 1.2e-7 |
| DR15, Y/N.x[N] | -0.4314403 | 0.1242463 | 12.06 | 0.0005* | -0.6749586 | -0.187922 | |
| DQA1-05, Y/N.x[N] | -0.4698848 | 0.1369336 | 11.78 | 0.0006* | -0.7382697 | -0.2014999 | |
| DQB1-0604.x | 1.04618729 | 0.4265861 | 6.01 | 0.0142* | 0.2100938 | 1.88228078 | |

Confidence limits are Wald-based.

For log odds of MAA-pos/MAA-neg

## Effect Wald Tests

| Source | Nparm | DF | Wald ChiSquare | Prob>ChiSq |
|---|---|---|---|---|
| C4 Protein (mg/L) | 1 | 1 | 28.0294271 | <.0001* |
| C4A.x | 1 | 1 | 6.65835122 | 0.0099* |
| C4L.x | 1 | 1 | 4.1639098 | 0.0413* |
| C4S/C4T | 1 | 1 | 4.19976436 | 0.0404* |
| C4B/C4T.x | 1 | 1 | 5.15463357 | 0.0232* |
| DR15, Y/N.x | 1 | 1 | 12.0579756 | 0.0005* |

# Nominal Logistic Fit for MAA-status...21

## Effect Wald Tests

| Source | Nparm | DF | Wald ChiSquare | Prob>ChiSq |
|---|---|---|---|---|
| DQA1-05, Y/N.x | 1 | 1 | 11.7750542 | 0.0006* |
| DQB1-0604.x | 1 | 1 | 6.01458101 | 0.0142* |

## Effect Likelihood Ratio Tests

| Source | Nparm | DF | L-R ChiSquare | Prob>ChiSq |
|---|---|---|---|---|
| C4 Protein (mg/L) | 1 | 1 | 32.5411491 | <.0001* |
| C4A.x | 1 | 1 | 6.70083218 | 0.0096* |
| C4L.x | 1 | 1 | 4.11461238 | 0.0425* |
| C4S/C4T | 1 | 1 | 4.08225583 | 0.0433* |
| C4B/C4T.x | 1 | 1 | 5.26262918 | 0.0218* |
| DR15, Y/N.x | 1 | 1 | 11.7906886 | 0.0006* |
| DQA1-05, Y/N.x | 1 | 1 | 12.6059219 | 0.0004* |
| DQB1-0604.x | 1 | 1 | 5.71618117 | 0.0168* |

## Odds Ratios

For MAA-status...21 odds of MAA-pos versus MAA-neg

### Unit Odds Ratios

Per unit change in regressor

| Term | Odds Ratio | Lower 95% | Upper 95% | Reciprocal |
|---|---|---|---|---|
| C4 Protein (mg/L) | 0.993605 | 0.991248 | 0.995968 | 1.0064364 |
| C4A.x | 3.720869 | 1.371523 | 10.09452 | 0.2687544 |
| C4L.x | 0.532753 | 0.290974 | 0.975434 | 1.8770412 |
| C4S/C4T | 0.114299 | 0.01436 | 0.909751 | 8.7489612 |
| C4B/C4T.x | 49.73551 | 1.705991 | 1449.961 | 0.0201064 |
| DQB1-0604.x | 2.846776 | 1.233794 | 6.568469 | 0.3512745 |

### Range Odds Ratios

Per change in regressor over entire range

| Term | Odds Ratio | Lower 95% | Upper 95% | Reciprocal |
|---|---|---|---|---|
| C4 Protein (mg/L) | 0.005996 | 0.000902 | 0.039859 | 166.76402 |
| C4A.x | 2653.79 | 6.65608 | 1058070 | 0.0003768 |
| C4L.x | 0.022864 | 0.000607 | 0.861364 | 43.736374 |
| C4S/C4T | 0.114299 | 0.01436 | 0.909751 | 8.7489612 |
| C4B/C4T.x | 49.73551 | 1.705991 | 1449.961 | 0.0201064 |
| DQB1-0604.x | 2.846776 | 1.233794 | 6.568469 | 0.3512745 |

### Odds Ratios for DR15, Y/N.x

| Level1 | /Level2 ^ | Odds Ratio | Prob>Chisq | Lower 95% | Upper 95% |
|---|---|---|---|---|---|
| Y | N | 2.369978 | 0.0005* | 1.45622 | 3.8571065 |
| N | Y | 0.4219449 | 0.0005* | 0.2592617 | 0.6867094 |

### Odds Ratios for DQA1-05, Y/N.x

| Level1 | /Level2 ^ | Odds Ratio | Prob>Chisq | Lower 95% | Upper 95% |
|---|---|---|---|---|---|
| Y | N | 2.5593919 | 0.0006* | 1.4963067 | 4.3777701 |
| N | Y | 0.3907178 | 0.0006* | 0.2284268 | 0.6683122 |

Normal approximations used for ratio confidence limits effects:
DR15, Y/N.x DQA1-05, Y/N.x
Tests and confidence intervals on odds ratios are Wald based.

## Nominal Logistic Fit for MAA-status...21

### Receiver Operating Characteristic

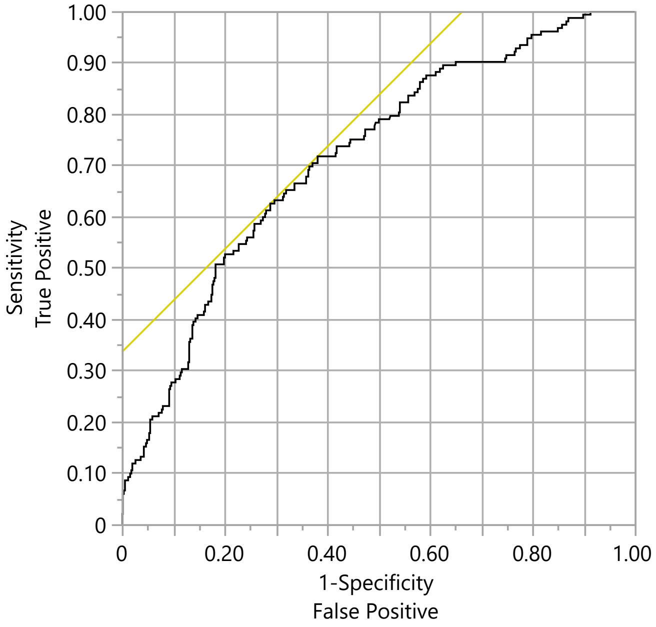


Using MAA-status...21='MAA-pos' to be the positive level

| AUC |
| --- |
| 0.71458 |

# Nominal Logistic Fit for MAA-PMSCl status

## Effect Summary

| Source | LogWorth | PValue |
|---|---|---|
| DQA1-05, Y/N.x | 5.911 | 0.00000 |
| C4 Protein (mg/L) | 3.943 | 0.00011 |
| C4A.x | 2.270 | 0.00538 |
| DQA1-01, 012.x | 1.845 | 0.01430 |
| DQB1-0603.x | 1.701 | 0.01992 |
| C4L/C4T | 1.670 | 0.02139 |
| DQB1-0601.x | 1.622 | 0.02388 |
| DQB1-0604.x | 1.382 | 0.04151 |

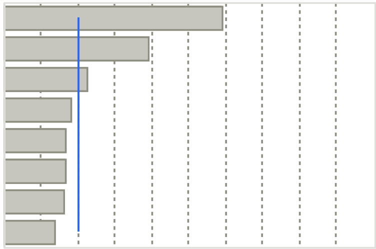

Converged in Gradient, 7 iterations

## Whole Model Test

| Model | -LogLikelihood | DF | ChiSquare | Prob>ChiSq |
|---|---|---|---|---|
| Difference | 32.67676 | 8 | 65.35352 | <.0001* |
| Full | 154.09262 | | | 4.11E-11 |
| Reduced | 186.76937 | | | |

| | |
|---|---|
| RSquare (U) | 0.1750 |
| AICc | 326.451 |
| BIC | 366.989 |
| Observations (or Sum Wgts) | 688 |

## Lack Of Fit

| Source | DF | -LogLikelihood | ChiSquare |
|---|---|---|---|
| Lack Of Fit | 668 | 154.09262 | 308.1852 |
| Saturated | 676 | 0.00000 | **Prob>ChiSq** |
| Fitted | 8 | 154.09262 | 1.0000 |

## Parameter Estimates

| Term | Estimate | Std Error | ChiSquare | Prob>ChiSq | Lower 95% | Upper 95% |
|---|---|---|---|---|---|---|
| Intercept | -1.3453188 | 0.7447974 | 3.26 | 0.0709 | -2.8050949 | 0.11445729 |
| C4 Protein (mg/L) | -0.0071625 | 0.0020205 | 12.57 | 0.0004* | -0.0111227 | -0.0032024 |
| DQA1-01, 012.x | -0.811197 | 0.3413587 | 5.65 | 0.0175* | -1.4802478 | -0.1421462 |
| DQA1-05, Y/N.x[N] | -1.4013123 | 0.3918215 | 12.79 | 0.0003* | -2.1692684 | -0.6333561 |
| DQB1-0601.x | 2.14431899 | 0.8796605 | 5.94 | 0.0148* | 0.42021601 | 3.86842197 |
| DQB1-0603.x | -1.8853124 | 1.0570387 | 3.18 | 0.0745 | -3.9570703 | 0.18644537 |
| DQB1-0604.x | 1.31608532 | 0.6038991 | 4.75 | 0.0293* | 0.13246484 | 2.49970579 |
| C4A.x | 0.70212339 | 0.2483901 | 7.99 | 0.0047* | 0.21528778 | 1.188959 |
| C4L/C4T | -1.5243778 | 0.6495141 | 5.51 | 0.0189* | -2.797402 | -0.2513537 |

Confidence limits are Wald-based.
For log odds of PM-Scl-pos/PM-Scl-neg

## Effect Wald Tests

| Source | Nparm | DF | Wald ChiSquare | Prob>ChiSq |
|---|---|---|---|---|
| C4 Protein (mg/L) | 1 | 1 | 12.5664799 | 0.0004* |
| DQA1-01, 012.x | 1 | 1 | 5.64716711 | 0.0175* |
| DQA1-05, Y/N.x | 1 | 1 | 12.7906675 | 0.0003* |
| DQB1-0601.x | 1 | 1 | 5.94221772 | 0.0148* |
| DQB1-0603.x | 1 | 1 | 3.18115534 | 0.0745 |
| DQB1-0604.x | 1 | 1 | 4.7494064 | 0.0293* |

## Nominal Logistic Fit for MAA-PMSCl status

### Effect Wald Tests

| Source | Nparm | DF | Wald ChiSquare | Prob>ChiSq |
|---|---|---|---|---|
| C4A.x | 1 | 1 | 7.99021351 | 0.0047* |
| C4L/C4T | 1 | 1 | 5.50818032 | 0.0189* |

### Effect Likelihood Ratio Tests

| Source | Nparm | DF | L-R ChiSquare | Prob>ChiSq |
|---|---|---|---|---|
| C4 Protein (mg/L) | 1 | 1 | 14.8893446 | 0.0001* |
| DQA1-01, 012.x | 1 | 1 | 6.00051727 | 0.0143* |
| DQA1-05, Y/N.x | 1 | 1 | 23.53339 | <.0001* |
| DQB1-0601.x | 1 | 1 | 5.10366677 | 0.0239* |
| DQB1-0603.x | 1 | 1 | 5.41850402 | 0.0199* |
| DQB1-0604.x | 1 | 1 | 4.15495278 | 0.0415* |
| C4A.x | 1 | 1 | 7.74863819 | 0.0054* |
| C4L/C4T | 1 | 1 | 5.29442913 | 0.0214* |

1.23e-6

### Odds Ratios

For MAA-PMSCl status odds of PM-Scl-pos versus PM-Scl-neg

#### Unit Odds Ratios

Per unit change in regressor

| Term | Odds Ratio | Lower 95% | Upper 95% | Reciprocal |
|---|---|---|---|---|
| C4 Protein (mg/L) | 0.992863 | 0.988939 | 0.996803 | 1.0071883 |
| DQA1-01, 012.x | 0.444326 | 0.227581 | 0.867494 | 2.2506004 |
| DQB1-0601.x | 8.536226 | 1.52229 | 47.86679 | 0.1171478 |
| DQB1-0603.x | 0.151782 | 0.019119 | 1.204959 | 6.5884126 |
| DQB1-0604.x | 3.728796 | 1.141639 | 12.17891 | 0.2681831 |
| C4A.x | 2.018033 | 1.240219 | 3.283661 | 0.495532 |
| C4L/C4T | 0.217756 | 0.060968 | 0.777747 | 4.5922856 |

#### Range Odds Ratios

Per change in regressor over entire range

| Term | Odds Ratio | Lower 95% | Upper 95% | Reciprocal |
|---|---|---|---|---|
| C4 Protein (mg/L) | 0.003306 | 0.00014 | 0.077775 | 302.51555 |
| DQA1-01, 012.x | 0.197425 | 0.051793 | 0.752547 | 5.065202 |
| DQB1-0601.x | 8.536226 | 1.52229 | 47.86679 | 0.1171478 |
| DQB1-0603.x | 0.023038 | 0.000366 | 1.451926 | 43.407181 |
| DQB1-0604.x | 3.728796 | 1.141639 | 12.17891 | 0.2681831 |
| C4A.x | 67.54137 | 3.639065 | 1253.574 | 0.0148057 |
| C4L/C4T | 0.217756 | 0.060968 | 0.777747 | 4.5922856 |

#### Odds Ratios for DQA1-05, Y/N.x

| Level1 | /Level2 ^ | Odds Ratio | Prob>Chisq | Lower 95% | Upper 95% |
|---|---|---|---|---|---|
| Y | N | 16.487863 | 0.0003* | 3.5491647 | 76.595381 |
| N | Y | 0.0606507 | 0.0003* | 0.0130556 | 0.2817564 |

Normal approximations used for ratio confidence limits effects:
DQA1-05, Y/N.x
Tests and confidence intervals on odds ratios are Wald based.

## Nominal Logistic Fit for MAA-PMSCl status

### Receiver Operating Characteristic

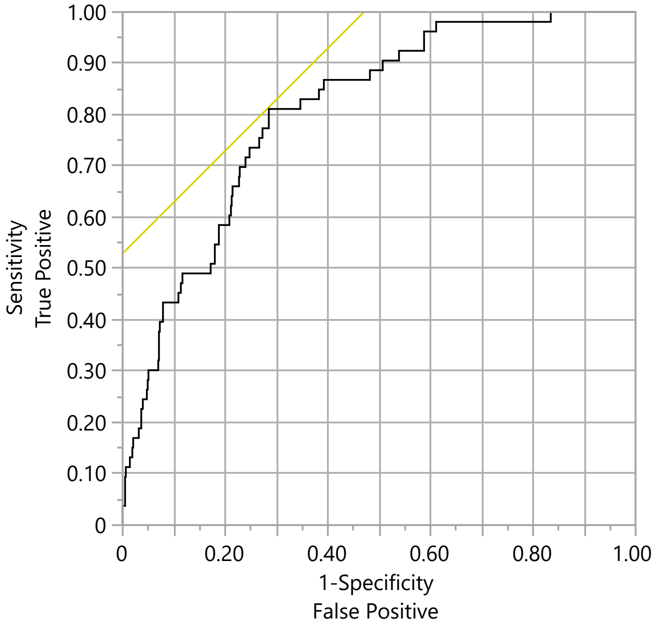


Using MAA-PMSCl status='PM-Scl-pos' to be the positive level

| AUC |
|---|
| 0.80594 |

0818-2022-IIM-5183 2 - Fit Nominal Logistic



# Nominal Logistic Fit for MAA-Ro-status

## Effect Summary

| Source | LogWorth | PValue |
|---|---|---|
| DQA1-03, Y/N.x | 4.935 | 0.00001 |
| DQA1-0101.x | 3.368 | 0.00043 |
| DQA1-02, Y/N.x | 2.872 | 0.00134 |
| C4S.x | 2.385 | 0.00412 |
| C4S/C4T | 2.188 | 0.00649 |
| C4 P/G | 1.769 | 0.01702 |
| C4T.x | 1.512 | 0.03077 |

Converged in Gradient, 6 iterations

## Whole Model Test

| Model | -LogLikelihood | DF | ChiSquare | Prob>ChiSq |
|---|---|---|---|---|
| Difference | 23.24819 | 7 | 46.49638 | <.0001* |
| Full | 171.89380 | | | 7.0e-8 |
| Reduced | 195.14199 | | | |

| | |
|---|---|
| RSquare (U) | 0.1191 |
| AICc | 360.005 |
| BIC | 395.87 |
| Observations (or Sum Wgts) | 672 |

## Lack Of Fit

| Source | DF | -LogLikelihood | ChiSquare |
|---|---|---|---|
| Lack Of Fit | 633 | 164.96233 | 329.9247 |
| Saturated | 640 | 6.93147 | **Prob>ChiSq** |
| Fitted | 7 | 171.89380 | 1.0000 |

## Parameter Estimates

| Term | Estimate | Std Error | ChiSquare | Prob>ChiSq | Lower 95% | Upper 95% |
|---|---|---|---|---|---|---|
| Intercept | 0.61637263 | 1.5932094 | 0.15 | 0.6988 | -2.5062603 | 3.73900559 |
| C4 P/G | -0.0128263 | 0.0056224 | 5.20 | 0.0225* | -0.0238459 | -0.0018067 |
| DQA1-0101.x | -1.1255709 | 0.3577133 | 9.90 | 0.0017* | -1.8266761 | -0.4244657 |
| DQA1-02, Y/N.x[N] | 0.76600024 | 0.2817044 | 7.39 | 0.0065* | 0.21386981 | 1.31813066 |
| DQA1-03, Y/N.x[N] | 0.8884321 | 0.244077 | 13.25 | 0.0003* | 0.41005007 | 1.36681414 |
| C4T.x | -0.8055032 | 0.3939399 | 4.18 | 0.0409* | -1.5776113 | -0.0333951 |
| C4S.x | 2.32970365 | 0.8896217 | 6.86 | 0.0088* | 0.58607715 | 4.07333015 |
| C4S/C4T | -7.4013753 | 3.0326967 | 5.96 | 0.0147* | -13.345352 | -1.4573989 |

Confidence limits are Wald-based.
For log odds of Ro-pos/Ro-neg

## Effect Wald Tests

| Source | Nparm | DF | Wald ChiSquare | Prob>ChiSq |
|---|---|---|---|---|
| C4 P/G | 1 | 1 | 5.20437283 | 0.0225* |
| DQA1-0101.x | 1 | 1 | 9.90091904 | 0.0017* |
| DQA1-02, Y/N.x | 1 | 1 | 7.39384987 | 0.0065* |
| DQA1-03, Y/N.x | 1 | 1 | 13.2493613 | 0.0003* |
| C4T.x | 1 | 1 | 4.18094593 | 0.0409* |
| C4S.x | 1 | 1 | 6.85789167 | 0.0088* |
| C4S/C4T | 1 | 1 | 5.95616716 | 0.0147* |

## Nominal Logistic Fit for MAA-Ro-status

### Effect Likelihood Ratio Tests

| Source | Nparm | DF | L-R ChiSquare | Prob>ChiSq |
|---|---|---|---|---|
| C4 P/G | 1 | 1 | 5.69433988 | 0.0170* |
| DQA1-0101.x | 1 | 1 | 12.4030444 | 0.0004* |
| DQA1-02, Y/N.x | 1 | 1 | 10.2814382 | 0.0013* |
| DQA1-03, Y/N.x | 1 | 1 | 19.223727 | <.0001* |
| C4T.x | 1 | 1 | 4.66550183 | 0.0308* |
| C4S.x | 1 | 1 | 8.229337 | 0.0041* |
| C4S/C4T | 1 | 1 | 7.40853647 | 0.0065* |

### Odds Ratios

For MAA-Ro-status odds of Ro-pos versus Ro-neg

#### Unit Odds Ratios

Per unit change in regressor

| Term | Odds Ratio | Lower 95% | Upper 95% | Reciprocal |
|---|---|---|---|---|
| C4 P/G | 0.987256 | 0.976436 | 0.998195 | 1.0129089 |
| DQA1-0101.x | 0.324467 | 0.160948 | 0.654119 | 3.0819759 |
| C4T.x | 0.446863 | 0.206468 | 0.967156 | 2.2378223 |
| C4S.x | 10.2749 | 1.796926 | 58.75229 | 0.0973246 |
| C4S/C4T | 0.00061 | 1.6e-6 | 0.232841 | 1638.2359 |

#### Range Odds Ratios

Per change in regressor over entire range

| Term | Odds Ratio | Lower 95% | Upper 95% | Reciprocal |
|---|---|---|---|---|
| C4 P/G | 0.070639 | 0.007248 | 0.688456 | 14.156543 |
| DQA1-0101.x | 0.105279 | 0.025904 | 0.427872 | 9.4985755 |
| C4T.x | 0.039875 | 0.001817 | 0.874957 | 25.078547 |
| C4S.x | 11145.76 | 10.42606 | 11915142 | 8.972e-5 |
| C4S/C4T | 0.00061 | 1.6e-6 | 0.232841 | 1638.2359 |

#### Odds Ratios for DQA1-02, Y/N.x

| Level1 | /Level2 ^ | Odds Ratio | Prob>Chisq | Lower 95% | Upper 95% |
|---|---|---|---|---|---|
| Y | N | 0.2161029 | 0.0065* | 0.0716286 | 0.6519812 |
| N | Y | 4.6274246 | 0.0065* | 1.5337867 | 13.960911 |

#### Odds Ratios for DQA1-03, Y/N.x

| Level1 | /Level2 ^ | Odds Ratio | Prob>Chisq | Lower 95% | Upper 95% |
|---|---|---|---|---|---|
| Y | N | 0.1691678 | 0.0003* | 0.0649831 | 0.4403876 |
| N | Y | 5.9112907 | 0.0003* | 2.2707272 | 15.38862 |

Normal approximations used for ratio confidence limits effects:
DQA1-02, Y/N.x DQA1-03, Y/N.x
Tests and confidence intervals on odds ratios are Wald based.

## Nominal Logistic Fit for MAA-Ro-status

### Receiver Operating Characteristic

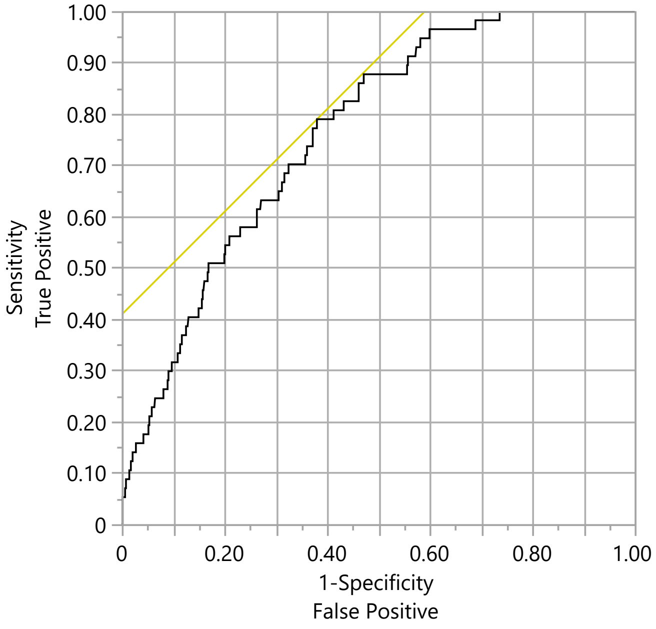


Using MAA-Ro-status='Ro-pos' to be the positive level

| AUC |
|---|
| 0.76202 |
